# The Impact of Health Information System Interventions on Maternal and Child Health Service Utilizations in Ethiopia: A Quasi-Experimental Study

**DOI:** 10.9745/GHSP-D-24-00145

**Published:** 2024-12-20

**Authors:** Abebaw Gebeyehu Worku, Wubshet Denboba Midekssa, Hibret Alemu Tilahun, Hiwot Tadesse Belay, Zeleke Abebaw, Afrah Mohammedsanni, Naod Wendrad, Mesoud Mohammed, Shemsedin Omer Mohammed, Amanuel Biru, Benti Ejeta Futassa

**Affiliations:** a JSI Research & Training Institute, Inc., Ethiopia Data Use Partnership, Addis Ababa, Ethiopia.; b Ethiopia Ministry of Health, Addis Ababa, Ethiopia.

## Abstract

**Background:**

Health information systems (HIS) are vital in supporting all aspects of managing health systems, financing, policymaking, and service delivery. A package of priority HIS interventions was piloted in selected woredas across all regions in Ethiopia. This study examined the impact of HIS interventions on maternal and child health (MCH) service utilization.

**Methods:**

A 2-arm quasi-experimental study was implemented in intervention and control woredas. Baseline and endline household and health facility surveys were conducted for both arms in 2020 and 2022, respectively. At baseline, 3,016 mothers and 167 health facilities were surveyed. At endline, 3,076 mothers and 160 health facilities were surveyed. The study used modified Performance of Routine Information System Management tools for the facility survey and a structured questionnaire for the household survey. Difference-in-difference (DID) analysis using mixed effect modeling was employed to measure changes and to account for clustering and control for likely confounders.

**Results:**

Intervention sites showed greater improvements in 75% of key HIS performance indicators. The changes in 90% of the MCH service utilization indicators were higher in the intervention sites. Significant (DID: *P*<.05) changes were observed in indicators including quality of antenatal care, skilled birth attendance, delivery at a health facility, family planning met need and unmet need, measles and second dose of rotavirus vaccination, and Vitamin A supplementation. BCG vaccination showed significantly higher improvement in the control sites. Other key indicators did not show significant changes.

**Conclusions:**

In many of the MCH service utilization indicators, the changes in the intervention sites were significantly higher compared to the control sites, but it was not universal. Scale-up of performance monitoring teams is crucial because it is one of the key pathways that links HIS performance with MCH service utilization. Outcome indicators that showed no or lower improvement require in-depth investigation.

## Abstract (precis)

Implementing health information system interventions to improve data quality improved the use of data for evidence-based decision-making at different health system levels and increased maternal and child health service utilization.

## Teaser

**Key Findings:**

- A package of interventions was implemented across all regions in Ethiopia to address the technical, behavioral, and organizational determinants of health information system (HIS) performance to improve the use of routine health data for evidence-based decision-making at all health system levels.
- To assess the impact of these interventions on HIS performance and maternal care and immunization service utilization, a facility and household survey were conducted at baseline and endline, showing that, compared to control sites, interventions sites showed greater improvements in 27 of 36 HIS performance outcomes, including reporting completeness, timeliness, accuracy, and information use.
- Compared to control sites, intervention sites showed greater improvements in 17 of 19 maternal and child health outcomes, including antenatal care, skilled birth attendance, postnatal care, and family planning, as well as immunization service indicators.

**Key Implication:**

- Investing in HIS interventions is one of the key strategies to strengthen HIS performance and improve maternal and child health service utilization.

## BACKGROUND

A health information system (HIS) is an integral part of a functioning health system.^1^^–^^3^ It is essential for generating information for planning, monitoring, and evaluation of public health initiatives and programs.^4^^,^^5^ It is assumed that improved HIS performance (data quality and use/data-driven decision-making) is a major factor for improvement in health service quality and uptake, which ultimately leads to better health outcomes. The 2 major maternal and child health (MCH) initiatives, Ending Preventable Maternal Mortality and Every Newborn Action Plan, have identified strategies to achieve the goals of reducing the ratios for maternal mortality (average global target of less than 70/100,000 live births by 2030), newborn mortality (target of 10 or less newborn deaths per 1,000 live births by 2035), and stillbirth (target of 10 or fewer stillbirths per 1,000 total births). Key indicators have been identified for these initiatives to monitor progress, with the assumption of mainly using routine health information.^6^^–^^8^ Evidence from low- and middle-income countries (LMICs) indicated that several priority MCH indicators could be tracked if the routine HIS is working well.^9^^–^^11^ Unlike national survey data, HIS data are more frequently collected and available at the district, subdistrict, and facility levels.^12^^,^^13^

Several studies, including from Ethiopia, indicated that the quality of routine data gathered in the health system is often poor and the use of routine health information for decision-making is low.^14^^,^^15^ A weak HIS is a major roadblock to reaching the health-related Sustainable Development Goals because the performance of health systems cannot be properly reviewed or monitored if HIS data are missing, incorrect, or delayed.^16^^–^^18^ HIS data provide information on what is happening in specific facilities in near real-time so that decision-makers (providers and facility managers all the way up to national decision-makers) can target and tailor their work for maximum impact and also quickly identify trends to make adjustments.^19^

Technical, behavioral, and organizational factors affect HIS processes and interventions, which, in turn, affect HIS performance (in terms of data quality and use) and, ultimately, health systems performance and health outcomes.^20^^,^^21^

To strengthen the HIS, the Ministry of Health (MOH) of Ethiopia has been implementing an Information Revolution (IR) strategy since 2016 in all woredas (about 1,160), although the intensity and scale of the implementation have varied across woredas. The implementation of IR within a health institution or an administrative level mainly targets progressively building a culture of using data for decision-making at all levels, where data management, analysis, and visualization practices are fully supported with digital technologies. Health institutions go through incremental stages of performance that include emerging, candidate, model, digital model, and demonstration site levels (center of excellence for IR).

The Data Use Partnership (DUP) project supports the MOH in addressing current HIS gaps based on the IR roadmap and HIS strategic plan.^22^^,^^23^ DUP is implemented by John Snow, Inc., the main MOH HIS partner. This project follows an embedment approach in which most of the technical staff are assigned to key departments and work together to plan, execute, and monitor HIS activities. Of the 191 MOH priority woredas, 19 (at least 1 woreda per region) were assigned to the DUP to make them IR model sites through the provision of a package of HIS interventions. We aimed to evaluate the effect of these HIS interventions in these woredas. Previous studies have indicated that improving HIS performance plays a key role in monitoring program performance, such as MCH, and improves service uptake and quality.^3^^,^^24^^–^^26^

Baseline data analysis showed that woredas with better HIS performance also had better maternal and immunization service utilization.^25^^,^^27^ Until recently, no evidence exists to prove that better health data lead to better health outcomes in LMICs.^28^ Hence, the relationship between HIS interventions and service utilization has not been investigated using rigorous methods. This study aimed to show whether increasing HIS performance had a causal effect on improved MCH outcomes.

The relationship between HIS interventions and service utilization has not been investigated using rigorous methods.

## METHODS

### Study Sites

**FIGURE 1:**
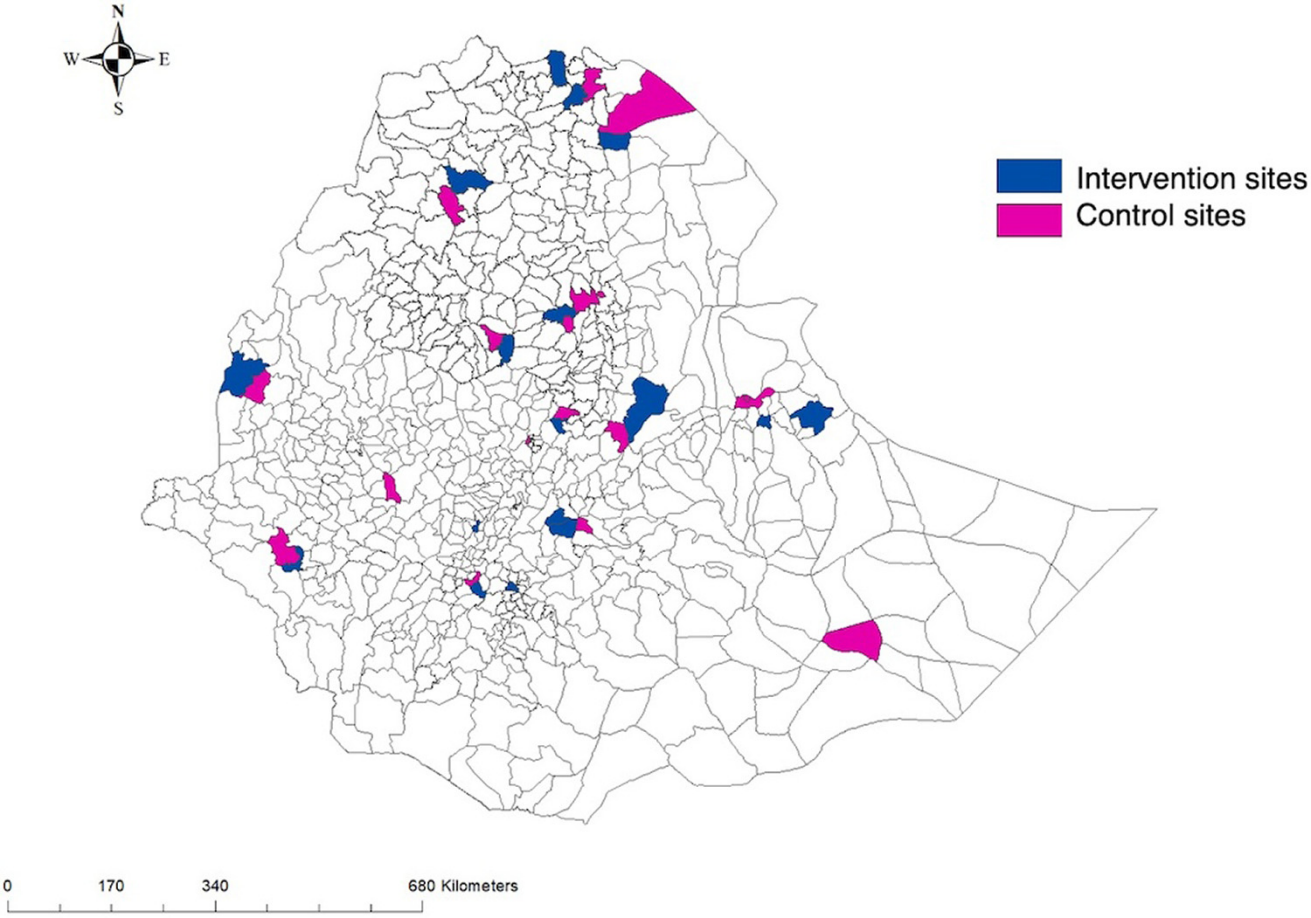
Map of the Locations of the Health Information System Intervention and Control Woredas, Ethiopia

The study covers all regions of Ethiopia (at least 1 woreda per region is included). The 19 intervention woredas of DUP were selected by regional-level health administrators with the assumption that they could be model sites. The main selection criteria were based on better performance in health service provision using key performance indicators. A total of 14 control woredas were purposively selected for comparison (Figure 1) in each region. The control woredas were nearby and did not have intensive HIS interventions implemented, including similar interventions by other implementing partners.

### Study Design

A 2-arm quasi-experimental study was implemented in intervention and control woredas. Linked health facility and population-based household surveys were conducted at both baseline and endline. Although the project started earlier, the preparation, initial assessments, and capacity-building activities took longer. Key facility-level interventions, like mentorship and performance monitoring team (PMT) activities with quality improvement interventions, were started after 2019. Accordingly, the baseline survey was conducted from September 2020 to October 2020, and the endline survey was conducted in May 2022. Alignment of the baseline survey and initiation of key interventions helped to minimize the time gaps in their implementation. The health facility survey focused on HIS performance (HIS intervention implementation, data quality, and use) in facilities that serve the selected kebele populations. A kebele is the lowest administrative unit, having about 1,000 households; a woreda is an administrative unit comprising 25–30 kebeles, 5–6 health centers, and 1 district hospital. Similar communities, not necessarily similar individuals, as that of the baseline were used at endline to evaluate the effectiveness of HIS interventions.

### Intervention Description

A combination of multiple approaches and tailored interventions were designed and implemented to create model HIS sites based on the IR objective of transforming and enhancing the culture of data use to positively impact population health and health system performance through evidence-based decision-making at all levels of the health system. Interventions were adapted to the site-specific context, based on continuous monitoring through needs assessments and feedback from quarterly supportive supervisions.

The interventions were designed to address the technical, behavioral, and organizational determinants of the HIS (Table 1). Updating data collection tools and data quality checklists, revising the health management information system (HMIS) indicators and reporting procedures, preparing HIS governance documents and the IR implementation guideline, and ensuring functional DHIS2 and electronic community health information systems were among the interventions for the technical factors.

**TABLE 1.** Summary of Interventions by Categories of HIS Determinants, Ethiopia

**HIS Determinants**	**Interventions**	**Target Audience**
Technical: Data collection forms, processes, systems, and methods for accomplishing HIS tasks	Update data collection tools and data quality checklists Revise HMIS Prepare reporting procedure manual, mentorship manual, HIS governance documents, IR implementation guideline Ensure functional DHIS2 and eCHIS systems	Health professionals, HIS staff, and managers doing HIS activities
Behavioral: The knowledge, skills, attitudes, values, and motivation of the people who produce and use data	Conduct hands-on capacity-building trainings Provide regular mentorship and coaching Provide supportive supervision Use nonfinancial incentives	Health professionals, HIS staff, and managers doing HIS activities
Organizational: Information use culture, structure, resources, and roles, and responsibilities of key contributors at each level of the health system	Produce quarterly analytic reports Conduct HIS-specific review meetings Monitor HIS performance (IR status of health institutions) Conduct quality improvement projects in health facilities Support resource mobilization Renovate medical record units Strengthen HIS governance structures Establish functional PMT	Health institutions (all levels – MOH administrative structures and health facilities), managers, health program leaders, and experts.

Abbreviations: eCHIS, electronic community health information system; HIS, health information system; HMIS, health management information system; IR, Information Revolution; MOH, Ministry of Health; PMT, performance monitoring team.

Behavioral factors (knowledge, skills, attitudes, values, and motivation of the people who produce and use data) were addressed through capacity-building trainings, regular mentorship and coaching, supportive supervision, and, in some facilities, nonfinancial incentives. The experts from the health system and universities provided hands-on capacity-building trainings and mentorship using the prepared training and mentorship manuals. Both data producers and users (health professionals, HIS staff, program experts, and health managers or decision-makers) were the target audiences (Table 1). Training topics included key HIS systems, HIS leadership, strategic problem-solving and analysis, revised HMIS indicators, quality improvement projects, IR implementation guideline, clinical audit, data recording and reporting, data analysis and report write-up, operationalizing PMT functionality, practice-based learning approach, and basic computer maintenance and troubleshooting. Regular mentorship and coaching sessions (i.e., every 2 months) were provided, and the activities included assigning trained mentors to each site and conducting baseline assessments to identify problems and their root causes. From there, joint action plans were created to solve these problems at the facility level. All activities were documented and shared with the local leadership to ensure accountability and sustain the changes made.

To address organizational factors, several activities were implemented (Table 1), including activities that promoted information use culture, HIS structure, resources (like renovation of medical units and resource mobilization), HIS governance systems, and establishing a functional PMT. Activities that promoted an information use culture included supporting intervention woredas and health facilities to produce quarterly analytic reports, conducting HIS-specific review meetings, monitoring HIS performance (IR status of health institutions), and conducting quality improvement projects in health facilities. HIS performance was monitored quarterly based on 3 criteria: HIS structure and resources (30%), data quality (30%), and data use (40%), using a detailed checklist prepared for woreda and health facilities (health post, health center, and hospital) (Supplement 1). The HIS staff/HIS focal person collected data and reported it as 1 HMIS indicator. Accordingly, IR progress of health institutions was defined as emerging if the health unit HIS performance score was less than 65%, candidate if the performance was between 65% and 90%, Model’ if the health unit HIS performance is >90%, ‘Digital Model’ or end state if the health unit HIS performance score is >90% and implementing electronic patient level and aggregate level data management systems.

In the intervention sites, regular support was provided to establish a functional PMT, a team of multidisciplinary health personnel at each level of the system that included members of the management committee (at health facilities, team leads and facility heads). Heads of health administrative units/health facilities served as chairpersons, and monitoring and evaluation unit heads or HMIS focal persons served as secretaries. The MOH recommended a tiered PMT establishment starting from the institution down to the team level. The PMT met monthly at all levels before a report was submitted to the next level. The team discussed coverage, equity, and quality of service indicators, as well as indicators of local importance and findings from any data sources. The main purpose was to make evidence-based decisions for health system performance (equity, quality, coverage, and efficiency). The PMT reviewed the MCH service utilization monthly and took immediate actions based on identified gaps (quality improvement projects, deployment of staff/rearrangements, and resource allocations). HIS performance and service uptake improvements were monitored quarterly by the team.

**FIGURE 2:**
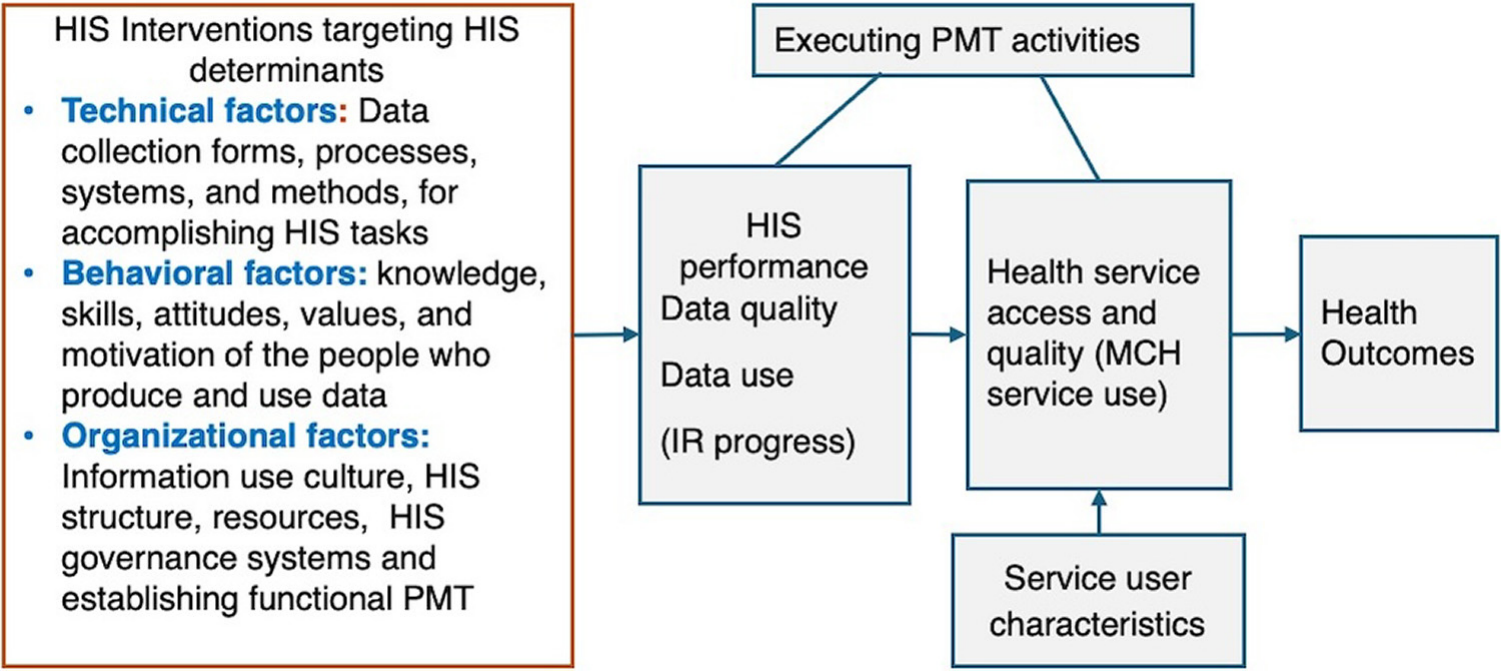
Conceptual Framework and Theory of Change for Health Information System Interventions and Impact on Service Delivery and Health Outcomes, Ethiopia Abbreviations: HIS, health information system; IR, Information Revolution; MCH, maternal and child health; PMT, performance monitoring team.

Figure 2 shows the framework/theory of change illustrating the pathway from increased utilization of quality data to improved service delivery and health outcomes. The PMT played an important mediator role between HIS performance and health service utilization and outcomes. Ensuring data quality and continuous use of information would result in improvement in access, utilization, coverage, and quality of health services.

The PMT played an important mediator role between HIS performance and health service utilization and outcomes.

### Facility Survey and Household Survey Sampling

#### Household Survey

The target population for the household survey was women with children aged 12–23 months. A 2-stage sampling technique was applied to select the sample population. In the first stage, kebeles were selected from the intervention and from the control woredas randomly. The selection was done from the list of kebeles per woreda. The number of selected kebeles was based on the population size of the regions. In the second stage, 29 targeted households (with 1 target mother per household) were selected randomly from each kebele. The sampling frame of targeted households was prepared either using the health post data or census.

The sample size calculation was based on a comparison of 2 independent proportions observed at 2 time periods (2 groups in 2 periods). In the first period, both groups were exposed to the control condition. In the second period, the intervention was rolled out in group 1 but not in group 2. Therefore, sample size calculation considered DID analysis and effect of clustering. We assumed a 10% change because of the intervention, a confidence level of 95%, a power of 80%, and design effect of 2. From the key MCH indicators in the 2019 Ethiopia Demographic Health Survey, the maximum sample size using contraceptive prevalence rate was taken. Because we needed to cover all targets in the selected clusters, the actual sample we collected was more than our estimate. At baseline, 3,016 mothers (1,740 intervention and 1,276 control) were selected from 102 kebeles (58 intervention and 44 control). Similarly, at endline, 3,077 mothers (1,743 intervention and 1,334 control) were selected from 106 kebeles (60 intervention and 46 control).

#### Health Facility Survey

For the health facility survey, health posts and health centers providing services for the catchment population in selected kebeles were included in the sample, along with a hospital (if available) in the selected woredas. At baseline, 167 health facilities (81 health posts, 71 health centers, and 15 hospitals) were included. Among the surveyed facilities, 96 (57.5%) received intervention. Similarly, at endline, 160 health facilities (80 health posts, 67 health centers, and 13 hospitals) were included. Of the surveyed facilities at endline, 84 (52.5%) received intervention.

### Data Collection and Procedures

A structured data collection tool was adapted from the Demographic and Health Survey questionnaire^29^ and Performance Routine Information System Management tools,^30^ designed on SurveyCTO software to collect the population and facility-based data (Supplement 2). The household questionnaire was translated into 3 local languages, Amharic, Oromiffa, and Tigrigna, and data collectors used the translated version during the interviews. The survey team received an intensive 5-day training using a structured training manual. Before data collection began, the questionnaires and the SurveyCTO program were pretested. All data collectors and supervisors were engaged in the pretesting translated tools. The data collection was done by 18 data collection teams (each team included 1 supervisor, 2 household data collectors, 1 health facility data collector, 1 guider, and 1 driver with a field car) and 1 coordinator, which took an average of 20 days.

### Data Analysis

Different data sets produced from the household and health facility surveys were carefully cleaned and linked to perform further analysis. Findings were presented in tables and graphs. Interpretation of changes was provided by comparing baseline and endline surveys and by intervention and control sites.

To evaluate the changes in HIS performance, scores of data quality, data use, and PMT functions were generated from health facility data. Similarly, the changes in the rates of MCH service utilizations were calculated based on the population-based data. To examine the impact of HIS interventions, MCH service utilization indicators were selected, including the presence of 4 or more ANC (ANC4) visits during pregnancy, delivery service by a skilled birth attendant (SBA), delivery at a health facility, postnatal care (PNC) by a skilled provider, maternal care composite indicator (consisting of maternal care services), immunization coverages, and met need for FP.

Using Stata version 14.0, DID analysis using mixed effect modeling was employed to measure statistical significance of the changes and account for clustering and control for likely confounders. We used 2 types of mixed effect (multilevel) models for the 2 types of outcome variables: (1) multilevel mixed effect logistic regression (melogit) for the binary outcomes and (2) mixed effects linear regression (mixed) for continuous outcome variables. Potential explanatory variables at the individual level included residence, religion, marital status of the mother, educational status of the mother and partner/husband, main occupation of the mother/partner, wealth index (computed from 10 asset variables), and household distance from health facilities (in kilometers). Similarly, 4 categories of facility-level HIS variables (from the facility survey), including HIS infrastructure, data quality, data use, and data management, were used for explanatory variables (Supplement 3).

### Ethical Approval

Ethical clearance was obtained from the Institutional Review Board of the Ethiopian Public Health Association. Support letters from the MOH, regional health bureaus, and woreda health offices were secured. A written consent form explaining the purpose and benefit of the study, participants’ right to withdraw at any point of the data collection process, confidentiality, and other associated issues was given to study participants to document their agreement to participate in the study.

## RESULTS

Overall, 55 key outcomes (36 under HIS performance and 19 under MCH) were measured in this study. Intervention sites showed greater improvements in 44 (80%) of the outcomes. Higher improvements were seen in 75% (27 of 36) of key HIS performance outcomes and 89.5% (17 of 19) of MCH outcomes.

Intervention sites showed greater improvements in 44 (80%) of the outcomes.

### Changes in Health Information System Performance

#### Reporting Completeness

At endline, all service, outpatient department, and quarterly reports that were supposed to be submitted to the woreda by the intervention facilities were submitted, but the control facilities did not reach 100% completeness. At baseline, none of the report types in the intervention facilities met the 90% reporting completeness threshold held by the MOH. Reporting completeness for inpatient department reports significantly improved in both intervention facilities (from 43% to 85%) and control facilities (65% to 100%). The average score increased by 21% (75.3% to 96.3%) in the intervention site and by 7.2% (91.3% to 98.5%) in the control sites (Supplement 4).

#### Reporting Timeliness

In intervention health facilities, reporting timeliness significantly increased for service reports (by 31%), outpatient department reports (by 33%), and quarterly reports (by 32%) but declined in inpatient department reports (by 10%). The control facilities decreased reporting timeliness for service, outpatient department, and quarterly reports but improved for inpatient department reports. The average score increased by 21.5% (56.8% to 78.3%) in the intervention sites but decreased by 17.7% (79% to 61.3%) in the control sites (Supplement 4).

#### Source Document Completeness

The source document completeness (a verification of primary source documents like registers and patient records) showed improvements in intervention sites, especially in SBA (67% to 79%), FP (79% to 95%), HIV (61% to 75%), and pneumonia (73% to 88%). Small changes were observed in third dose of the pentavalent vaccine (Penta3), malaria, and TB in both intervention and control facilities (Supplement 4).

#### Report Accuracy

Report accuracy within the acceptable range (between 90% and 110%) showed improvements in 5 indicators, including SBA, Penta3, FP, pneumonia, and TB in both intervention and control health facilities. However, it decreased for HIV and malaria in both intervention and control facilities (Table 2). Compared to baseline, the issue of underreporting (report accuracy above 110%) considerably decreased in SBA, Penta3, FP, and pneumonia in the intervention facilities and in the case of Penta3, FP, and pneumonia in control facilities. However, overreporting **(**report accuracy under 90%) was a persistent problem in many indicators, especially in control facilities (over 20%) (Table 2).

**TABLE 2.** Report Accuracy of the Key Indicators in the Intervention and Control Health Facilities According to the Baseline and Endline Surveys, Ethiopia

	Control				Intervention				Change in AR	
Indicators	No.	AR, %	OR, %	UR, %	No.	AR, %	OR, %	UR, %	Control	Inter
**Baseline**										
SBA	37	68	30	3	48	83	10	6		
Penta	32	56	22	22	41	73	12	15		
FP	36	36	56	8	48	63	31	6		
HIV	37	86	5	8	49	82	10	8		
Malaria	34	59	21	21	46	70	22	9		
Pneumonia	37	30	51	19	49	43	29	29		
TB	34	71	15	15	49	71	16	12		
**Endline**										
SBA	38	82	11	8	42	98	2	0	+14	+15
Penta	35	69	23	9	40	75	15	10	+13	+2
FP	38	76	21	3	42	81	14	5	+40	+18
HIV	22	82	14	5	27	78	7	15	−4	−4
Malaria	30	53	27	20	28	50	29	21	−6	−20
Pneumonia	38	66	21	13	42	76	17	7	+36	+33
TB	27	78	4	19	34	82	6	12	+7	+11

Abbreviations: AR, acceptable range; FP, family planning; OR, overreporting; SBA, skilled birth attendance; UR, underreporting.

### Information Use

On average, there were improvements in all 7 elements of data use in intervention sites and improvements for some elements in control sites. For 5 of the elements, the positive change was larger in intervention sites. For 2 of the elements (use of data for quality improvement and use of data for performance review), the positive change was larger in control sites than intervention sites. The changes in the use of data for HMIS quality improvement were 27% to 70% in the control and 53% to 80% in the intervention facilities. The changes in the average score of quality control practice improved from 78% to 94% in the intervention sites and from 56% to 67% in the control sites. The scores for use of routine data for performance review increased in both intervention (60% to 82%) and control (31% to 69%) health facilities. The average score for the level of data analysis practice for both intervention and control sites reached 100% (all facilities are engaged in data analytic practices). Moreover, the proportion of intervention facilities having evidence of displays was increased from 86% to 95% and it was from 78% to 82% in the control facilities. The proportion of intervention facilities that produced any report or bulletin based on HMIS data increased from 37% to 43% but decreased in the control sites from 35% to 21%. The overall information dissemination score for facilities was low for both groups and did not show significant changes: no change in the control sites and 28% to 30% in the intervention sites (Table 3).

**TABLE 3.** Proportion of Intervention and Control Health Facilities Use of Routine Data and Dissemination of Information According to Key Indicators of Data Use, Ethiopia

	**Baseline (2020), %**		**Endline (2022), %**		**Changes, %**		
	**Control** **(n=37)**	**Intervention** **(n=49)**	**Control** **(n=38)**	**Intervention** **(n=42)**	**Control**	**Intervention**	**DID**
Use of routine data for quality improvement (max 5)	27	53	70	80	+43	+27	−16
Data use for quality control practice score (max 6)	56	78	67	94	+11	+16	+5
Use of routine data for performance review and evidence-based decision making (max 6)	31	60	69	82	+38	+22	−16
Average score for level of data analysis practice (max 7)	100	63	100	100	0	+37	+37
HFs prepare data visuals/displays	78	86	82	95	+4	+9	+5
HFs produce any report or bulletin based on HMIS	35	37	21	43	−14	+6	+20
Average score for disseminating RHIS information to stakeholders outside of the health sector	21	28	21	30	0	+2	+2

Abbreviations: DID, difference-in-difference; HF, health facility; HMIS, health management information system; RHIS, routine health information system.

#### Performance Monitoring Team Functionality

Health facilities in the intervention sites show higher improvement in 7 of 8 PMT activities compared to the control sites. The control health facilities showed better improvement only in 1 of the 8 functions. Both groups showed improvement in presence of PMT structure, availability of at least 1 PMT meeting minutes, and discussed performance using HMIS data. This indicator was assessed by reviewing whether annual plans reflected the use of HMIS data for problem identification and target setting (Table 4).

**TABLE 4.** Changes in Performance Monitoring Team Functions at Health Facility Level in Intervention and Control Sites, Ethiopia

	**Baseline (2020), %**		**Endline (2022), %**		**Change, %**		
Indicators	Control(n=37)	Intervention(n=49)	Control(n=38)	Intervention(n=42)	Control	Intervention	DID
HFs with PMT	95	98	92	98	−3	0	+3
HFs having regular PMT (≥3 in assessed 3 months)	54	60	66	90	+12	+30	+18
Availability of at least 1 PMT meeting minutes	81	80	89	93	+8	+13	+5
PMT meetings chaired by facility director (all 3 months)	63	68	53	85	−10	+17	+27
HFs discussed performance using HMIS data	54	57	89	93	+35	+36	+1
HFs identified performance- related problems	63	67	74	86	+11	+19	+8
HFs conducted root cause analysis	47	49	66	79	+19	+30	+11
HFs developed action plan to improve performance	47	61	71	81	+24	+20	−4

Abbreviations: DID, difference-in-difference; HF, health facility; HMIS, health management information system; PMT, performance monitoring team.

### Changes in Maternal Care Services Quality and Utilization

The changes in the key ANC indicators, ANC4 and quality of ANC, showed higher improvement in the intervention sites compared to control sites. The proportion of mothers receiving at least 4 ANC visits from a skilled health provider during their pregnancy increased from 55% at baseline to 66% at endline in the intervention sites and from 52% to 60% in the control sites. The odds of change in the intervention sites were higher but not significant compared to control sites (DID_melogit_; adjusted odds ratio [AOR]= 1.06; 95% confidence interval [CI]=0.82, 1.36). The change was high in receiving essential ANC service components (quality of ANC services) in the intervention sites (from 37% to 57%) compared to the control sites (from 37% to 38%). It is significantly improved in the intervention sites compared with the control sites (DID_mixed_; coef=1.17; 95% CI=0.79, 1.55) (Table 5).

**TABLE 5.** Changes in Maternal Service Quality and Utilizations in Intervention and Control Sites Across Baseline and Endline Surveys, Ethiopia

	**Baseline**	**Intervention, No. (%)**	**Endline**	**Intervention, No. (%)**	**DID_melogit_ (Time**^a^ **Group)**		
**Variables**	**Control, No. (%)**		**Control, No. (%)**		**Coef/AOR (95% CI)**	***P* Value**	**Statistical Model**
ANC4+	655 (52)	948 (55)	803 (60)	1,144 (66)	1.1 (0.8, 1.4)	.67	Melogit
Quality of ANC	466 (37)	641 (37)	501 (38)	988 (57)	1.8 (0.8, 1.6)	<.01	Mixed
SBA	974 (77)	1,274 (75)	997 (75)	1,413 (81)	2.0 (1., 2.7)	<.01	Melogit
HF delivery	976 (78)	1,273 (74)	988 (74)	1,395 (80)	2.0 (1.4, 2.7)	<.01	Melogit
PNC	903 (72)	1,191 (70)	1,004 (75)	1,353 (78)	1.3 (0.9, 1.7)	.12	Melogit
Early PNC	784 (62)	1,057 (62)	881 (66)	1,199 (69)	1.1 (0.8, 1.5)	.42	Melogit
^a^ Overall maternal service, mean	71	71	72	77	4.0 (1.2, 6.8)	<.01	Mixed
Total, No.	1,257	1,710	1,333	1,743			

Abbreviations: ANC, antenatal care, ANC+4, 4 antenatal care visits; AOR, adjusted odds ratio; DID, difference-in-difference; HF, health facility; PNC, postnatal care; SBA, skilled birth attendance.

^a^ The mean maternal care (composite indicator) is computed from key indicators of prenatal, intrapartum, and postnatal maternal service utilizations and includes ANC4+, early ANC, tetanus toxoid vaccine, HIV test (as part of prevention of mother-to-child transmission), iron folate supplement, SBA, health facility delivery, PNC, and early PNC (in the first 24 hours).

The changes in the key ANC indicators showed higher improvement in the intervention sites compared to control sites.

The proportion of births attended by skilled health personnel (SBA) significantly improved in the intervention sites (75% to 81%) but decreased slightly in the control sites (77% to 75%). The odds of changes of SBA were 2 times higher in the intervention sites (DID_melogit_; AOR= 2; 95% CI=1.4, 2.7) compared to control sites. A similar change was also observed in delivery at health facilities (Table 6). Like the delivery service, the change in PNC service utilization was higher in the intervention sites, increasing from 70% to 78%, compared to control sites, which increased from 72% to 75%, but the difference was not statistically significant (DID_melogit_; AOR= 1.3; 95% CI=0.9, 1.7). The change in early PNC service utilization was also not significantly different in the 2 groups (DID_melogit_; AOR= 1.1; 95% CI=0.8, 1.5) (Table 5).

**TABLE 6.** Changes in Child Immunization Service in Intervention and Control Sites Across Baseline and Endline Surveys, Ethiopia

	**Baseline**		**Endline**		**DID_melogit_**^a^ **(Time**^a^ **Group)**	
	**Control**	**Intervention**	**Control**	**Intervention**	**AOR (95% CI)**	***P* Value**
OPV3, %	85	88	80	89		
Penta3, %	85	87	81	88	1.4 (0.9, 2.3)	.144
PCV3, %	83	87	80	89		
MCV, %	75	75	63	79	1.9 (1.3, 2.9)	.002
Rota2, %	88	92	86	92	1.5 (1.1, 2.2)	.016
Total, No.	785	1,115	610	960		
BCG, %	89	92	93	93	0.6 (0.4, 0.9)	.018
Total, No.	1,133	1,603	1,130	1,552		
Vitamin A, %	85	77	80	86	2.7 (2.0, 3.6)	<.001
Full vacc, %	44	48	49	52	1.1 (0.8, 1.4)	.690
Total, No.	1,276	1,740	1,334	1,743		

Abbreviations: AOR, adjusted odds ratio; CI, confidence interval; DID, difference-in-difference; MCV, meningococcal vaccine; OPV, oral polio vaccine; Penta3, third dose of the pentavalent vaccine; Rota2, second dose of the rotavirus vaccine.

^a^ melogit: The statistical significance of health information system intervention using DID was done by controlling the effect of health facilities clustering and key potential confounders like wealth index, maternal education, and religion using multilevel (mixed effect) logistic regression.

The improvement in overall maternal service utilization based on the maternal composite indicator (score) was higher in the intervention sites (71 to 77) compared to control sites (71 to 72). The change was significantly higher in the intervention sites compared to the control sites (DID_mixed_; coef=4.0; 95% CI=1.2, 6.8) (Table 5).

Among controlled variables, wealth index and maternal education were significantly associated in all fitted models of ANC4, quality of ANC, SBA, health facility delivery, PNC, early PNC, and maternal composite indicator. Details of mothers and household background information are provided in Supplement 4.

### Changes in Child Immunization Service Utilization

A statistically significant impact of HIS interventions on child immunization service was observed in many indicators, including vitamin A supplementation (DID_melogit_; AOR=2.7, 2.0–3.6), measles vaccination (DID_melogit_; AOR=1.9, 1.3–2.9), and second dose of rotavirus vaccination (DID_melogit_; AOR=1.5, 1.1- 2.2). Measles vaccination increased from 75% to 79% in the intervention sites but decreased from 75% to 63% in the control sites. Similarly, vitamin A supplementation increased from 77% to 86% in the intervention sites but decreased from 85% to 80% in the control sites. Although improvements were observed in both groups, the change in BCG immunization was significantly higher in the control sites (89% to 93%) compared to intervention sites (92% to 93%), but there was a wide confidence interval (DID_melogit_; AOR=0.6, 0.4–0.9). Penta3 immunization also slightly increased in the intervention sites (87% to 88%) but decreased in the control sites (85% to 81%). Full immunization (receiving all required doses of all 6 vaccines) increased from 48% to 52% in the intervention sites and increased from 44% to 49% in the control sites. There was no significant change in both full and penta3 vaccinations (Table 6).

### Changes in Family Planning Service Utilization

The contraceptive prevalence rate (met need) increased from 58% to 64% in the intervention sites but decreased from 59% to 56% in the control sites. Hence, the impact of HIS intervention on contraceptive use was significant (P<.03); the change was 30% higher in the intervention sites compared to control (DID_melogit_; AOR=1.3, 1.1–1.7). The intervention sites reduced the unmet need for FP significantly from 33% to 24%, but this increased from 29% to 34% in the control sites ((DID_melogit_; AOR=0.6, 0.5 – 0.8) (Table 7). Significant association was also observed among controlled variables, including maternal education, religion, FP knowledge score, and wealth index in both met need and unmet need models.

**TABLE 7.** Changes in FP Service Use in Intervention and Control Sites Across Baseline and Endline Surveys

	**Baseline**		**Endline**		**DID**_melogit_^a^ **(Time**^a^ **Group)**	
	**Control**	**Intervention**	**Control**	**Intervention**	**AOR (95% CI)**	***P* Value**
No need, %	12.57	9.53	9.6	9.58		
Unmet need, %	28.72	32.63	34.28	25.93	0.6 (0.5, 0.8)	<.01
Met need, %	58.71	57.84	56.11	64.49	1.3 (1.1, 1.7)	.03
No.	1,257	1,710	1,333	1,743		

Abbreviations: AOR, adjusted odds ratio; CI, confidence interval; FP, family planning.

^a^ melogit: The statistical significance of HIS intervention using DID was done by controlling the effect of health facilities clustering and key potential confounders maternal education, religion, FP knowledge score, and wealth index using multilevel mixed effect logistic regression.

## DISCUSSION

This evaluation study measured the effectiveness of a package of priority HIS interventions to improve HIS performance as well as its impact on MCH service utilization. The study findings revealed that higher improvement was observed in 75% of HIS performance indicators in the intervention sites compared to control sites. Consequently, the change in 9 of 10 key MCH service utilization indicators in the intervention sites was higher compared to the control sites.

The HIS interventions significantly contributed to greater improvement of data quality indicators, such as reporting completeness, timeliness, and source document completeness, in intervention sites compared to control sites. Previous study reports showed that most of such data quality indicators have suboptimal performance, usually below the national target of 90%.^25^^,^^27^^,^^31^ Improvement in data quality control in intervention sites is also an indication of the commitments to improve data quality, which has been recommended in previous studies.^32^^,^^33^ However, there were data quality inconsistencies in some of the assessed indicators, especially data accuracy. Both overreporting **(**report accuracy under 90%) and underreporting (report accuracy above 110%) were persistent issues even in the intervention sites. For example, malaria and HIV data have such problems. This finding implies that an in-depth investigation into the reasons and tailored interventions are needed to address data accuracy issues during the implementation of programs. Several studies indicated that quality data is crucial in designing, monitoring, and evaluating public health programs or policies.^32^^,^^34^^–^^38^

The HIS interventions significantly contributed to greater improvement of data quality indicators.

The changes in data use indicators also showed the contribution of HIS interventions. In the intervention sites, the score for the use of routine data quality improvement, performance review and evidence-based decision-making, data analysis practice, and data visuals/displays increased. One of the promising findings was that the majority of the intervention facilities improved PMT functions compared with the control groups for 7 of 8 PMT functions. In the Ethiopian health sector, PMTs are considered critical platforms for information use and several decision-making activities at the health facility and woreda levels. Previous studies in Ethiopia have indicated that the use of routine health information by health workers and health managers is low and recommended to have HIS interventions implemented.^15^^,^^31^^,^^39^ The finding implies that the scale-up of these interventions in other woredas is critical for better use of routine health data for decision-making at different levels of the health system.

Key MCH indicators were monitored to measure the impact of HIS interventions on program performance, service uptake, and quality. Maternal care service indicators, including ANC4, quality of ANC, SBA, and PNC, showed higher or significant changes in the intervention sites compared with the control sites. The overall maternal service utilization based on the maternal composite indicator (score) is significantly higher in the intervention sites compared to control sites. Among child health services, the immunization program was monitored based on key service coverage indicators. Accordingly, significant changes were observed in immunization services indicators like measles, second dose of rotavirus, and vitamin A supplementation in the intervention sites compared to control sites. Improvements were also seen in other immunization indicators like BCG, Penta3, and full immunization in the intervention sites but control sites had a significant change in BCG immunization. Overall, the findings indicated that HIS interventions are key factors for immunization program performance. Previous studies suggested the theoretical frameworks about how routine HIS can improve health systems functioning, especially in LMICs.^3^^,^^24^^,^^25^^,^^27^ However, evidence is limited on the pathways that connect HIS performance with health service utilization and outcomes. For example, a scoping review of LMICs reflected the lack of sufficient evidence on data’s direct impact on health service delivery, behaviors, and outcomes.^28^ PMT is an important intermediate factor that can link HIS performance with the use of health services and health outcomes. The findings of our study also suggest that establishing a functional routine HIS is a priority intervention for monitoring and improving MCH program performances, service uptake, or quality of care. The continuous use of information helps to guide day-to-day operations, track performance, learn from past results, and improve service delivery.

Another interesting finding of this study is the significant contribution of HIS interventions to the FP program. The growing intention to space/limit childbirth was observed in both intervention and control sites. Similar to maternal health services utilization, HIS interventions brought a significant change in addressing the FP needs of mothers. The improvement in contraceptive prevalence rate (met need for FP) was significantly higher in the intervention sites compared to the control sites. Consequently, intervention woredas were able to achieve a significant reduction of unmet need for FP compared to the control woredas. The most preferred contraceptive methods were injectables (about 60%) and implants (about 30%) in both surveys. The finding is similar to study reports of different community-based surveys in Ethiopia.^40^^–^^42^

### Limitations

The findings of the study should be interpreted by considering the following limitations. Even though the starting time of the interventions is known, the study used cross-sectional studies at 2 time points. The temporal relationship of events occurring in the period may not be clear; MCH outcomes can be improved before HIS performance outcomes. Moreover, some maternal service utilization, like ANC, occurred sometime before the date of the survey (at least 1 year back). However, this problem is not like that of a single cross-sectional study, which is affected by the well-known “chicken-and-egg” dilemma. Second, the provision of “equal weight” may not necessarily reflect the contributions in the calculation of composite scores. In most of the composite indicators, calculations were done based on a series of questions with binary outcomes. These questions have usually provided equal weight with “0” for “no/absent” responses and “1” for “yes/present” responses. Third, study sites were not randomized to receive intervention or not. The intervention woredas were better performers in health service indicators. HIS or MCH performance improvement will be easier at those sites and could overestimate the intervention effects. Fourth, spillover effects or contamination of interventions can happen if trained staff are transferred or clients move from 1 site to another site and receive services there. Finally, although all regions were included, the study findings can be generalizable only to the project sites.

## CONCLUSIONS

Overall, intervention sites showed greater improvements in 80% of the outcome indicators. The finding indicates that there are visible improvements in some dimensions of data quality and data use (75% of HIS performance outcomes). However, the intervention showed no or lower contribution in some aspects of HIS performance measures (25%).

With all the potential limitations, the changes in most (about 90%) of the MCH service utilization indicators were higher in the intervention sites compared with the control sites. The study indicated that investing in HIS interventions, including scale-up of PMT, is one of the key strategies to strengthen HIS performance and improve MCH service utilization. Outcome indicators that showed no or lower improvement require in-depth investigation.

## Supplementary Material

GHSP-D-24-00145-Supplement2.pdf

GHSP-D-24-00145-Supplement3.pdf

GHSP-D-24-00145-Supplement1.pdf

GHSP-D-24-00145-Supplement4.pdf

## Notes

**Peer Reviewed**
