# Supplementary material for: The Impact of Health Information System Interventions on Maternal and Child Health Service Utilizations in Ethiopia: A Quasi-Experimental Study
Source: Glob Health Sci Pract. 2024 Dec 20;12(6):2400145. doi: 10.9745/GHSP-D-24-00145 (PMC11666088; doi:10.9745/GHSP-D-24-00145)
Supplement: GHSP-D-24-00145-Supplement2.pdf [file GHSP-D-24-00145-Supplement2.pdf]

Supplement to: Worku AG, Midekssa WD, Tilahun HA, et al. The impact of health information system interventions on maternal and child health service utilizations in Ethiopia: quasi-experimental study. *Glob Health Sci Pract*. 2024;12(6):2400145. https://doi.org/10.9745/GHSP-D-24-00145

Supplement 2. MOH and Ethiopia Data Use Partnership Project

Population-based (HH) survey Household, Women and Child Questionnaire

CONSENT				
Consent will be asked for eligible households which are selected by the systematic random sampling for the survey. Eligibility is defined as a household with woman with live birth in last 12-23 months and households with a child aged 12-23 months.				
	Note for data collector: Ask head of household if you can speak with the woman who has given birth within the last 12-23 months.			
	Hello. My name is ----------From ----------responsibility-----------. We are here to conduct a survey about women and children health services. We would very much appreciate your participation in this survey. Your household is selected randomly for this study. The information collected here today will be used to improve health services. I will ask some questions about household members, and then I will ask to interview certain household members to talk about health and about issues related to maternal and child health services. There are no risks involved in participating in the study. Whatever information you provide will be kept strictly confidential and will not be shown to other persons. The interview requires about 30-40 minutes for completing. Participation in this survey is voluntary.<br>Do you want to ask me anything about the survey?			
**HH_01**	May I begin the interview?	1. Yes ☐	0. No ☐	If "No" proceed to end.

Household and Member Information			
**HH_01**	Date of interview (today's date)		
**HH_02**	Interviewer's name		
**HH_03**	Please select region	Addis Ababa<br>Afar<br>Amhara<br>Beneshangul Gumuz<br>Dire Dawa<br>Gambella<br>Harari<br>Oromiya	

Supplement to: Worku AG, Midekssa WD, Tilahun HA, et al. The impact of health information system interventions on maternal and child health service utilizations in Ethiopia: quasi-experimental study. *Glob Health Sci Pract*. 2024;12(6):2400145. https://doi.org/10.9745/GHSP-D-24-00145

		SNNPR<br>Somali<br>Tigray	
**HH_04**	Select Zone/Sub-city		
**HH_05**	Woreda		
**HH_06**	Enter Kebele name(code)		
**HH_07**	House number		
**HH_08**	Name of household head		
**HH_09**	Household ownership	1. Own<br>2. Rental<br>96. Others ___________	
**HH_09o**	If other, please specify		
**HH_10**	Main material of the floor	1. Natural floor; earth/sand<br>2. Dung<br>3. Finished floor ; cement/bricks<br>96. Others	
**HH_10o**	If other, please specify		
**HH_11**	Main material of the roof<br><br>Record observation	1. Natural roofing<br>2. Thatch/leaf<br>3. Finished roofing<br>4. Corrugated iron<br>96. Others	
**HH_11o**	If other, please specify		

Supplement to: Worku AG, Midekssa WD, Tilahun HA, et al. The impact of health information system interventions on maternal and child health service utilizations in Ethiopia: quasi-experimental study. *Glob Health Sci Pract*. 2024;12(6):2400145. https://doi.org/10.9745/GHSP-D-24-00145

**HH_12**	What is the main source of drinking water for members of your household?	1. Piped water<br>2. Open well<br>3. Covered well/borehole<br>4. Spring<br>5. River/stream<br>96. Other		
**HH_12o**	If other, please specify			
**HH_13**	What kind of toilet facility do members of your household usually use?	0. No facility/bush/field<br>1. Flush toilet<br>2. Traditional pit toilet<br>3. Ventilated improved pit (VIP) Latrine<br>96. Other		
**HH_13o**	If other, please specify			
**HH_14**	Does your household have the following?	1. Yes	0. No	
**A**	Electricity	1. Yes	2. No	
**B**	Radio	1. Yes	2. No	
**C**	Television	1. Yes	2. No	
**D**	Landline telephone	1. Yes	2. No	
**E**	Cell phone	1. Yes	2. No	
**F**	Refrigerator	1. Yes	2. No	
**HH_15**	Does any member of this household own land that can be used for agriculture?	0. No<br>1. Yes		
**HH_16**	Does any member of this household have an account with a bank/credit association/micro finance?	0. No<br>1. Yes		
**HH_17**	How many family members live in this household?			
**HH_18**	You stated there is a child in your household who is between 12-23 months of age (1-2 years old). Is that correct?	0. No<br>1. Yes		If No End

Supplement to: Worku AG, Midekssa WD, Tilahun HA, et al. The impact of health information system interventions on maternal and child health service utilizations in Ethiopia: quasi-experimental study. *Glob Health Sci Pract*. 2024;12(6):2400145. https://doi.org/10.9745/GHSP-D-24-00145

**HH_19**	What is this child's name?		
**HH_20**	Are you the mother for {child name}?<br><br>*If "No" ask to speak to the mother of the child.*	Yes……………………………………..1<br>No………………………………………0	If "yes" skip to HH_22
**HH_21**	Are you the primary caregiver for (child name), e.g., you care for the child and take them to clinic when sick?<br>*If "No" ask to speak to caregiver.*	Yes……………………………………..1<br>No………………………………………0	
**HH_22**	Enter child's date of birth:		
**HH_23**	Enter child's age (in months):<br><br>*If child is below 12 months or above 23 months skip to end of survey.*		
**HH_24**	Enter sex of child:	1. Male<br>2. Female	

**Mother/Caregiver Background**			
**HH_25**	How long have you been living continuously in this Kebele?	Years . . . .<br>Always	
**HH_26**	How old were you at your last birthday?	AGE IN COMPLETED YEARS: ____/______	
**HH_27**	What is your current marital status?	1. Married/ living together<br>2. Single<br>3. Divorced / separated<br>4. Widowed	
**HH_28**	Have you ever attended school?	0. No<br>1. Yes	
**HH_29**	What is your educational level?	1. Elementary(1-8) /read and write<br>2. Secondary(8-10/12)<br>3. Tech./voc. level<br>4. Higher level	
**HH_30**	What is the educational level your partner/husband?	0. No education<br>1. Elementary(1-8) /read and write<br>2. Secondary(8-10/12)<br>3. Tech./voc. Level<br>4. Higher level	

Supplement to: Worku AG, Midekssa WD, Tilahun HA, et al. The impact of health information system interventions on maternal and child health service utilizations in Ethiopia: quasi-experimental study. *Glob Health Sci Pract*. 2024;12(6):2400145. https://doi.org/10.9745/GHSP-D-24-00145

**HH_31**	What is your religion?	1. Orthodox<br>2. Catholic<br>3. Protestant<br>4. Muslim<br>5. Traditional<br>96. Other	
**HH_31o**	If other, please specify		
**HH_32**	What is your occupation, that is, what kind of work do you mainly do?	Employed in private sector……………….1<br>Government employee/civil service…….2<br>Agriculture - works on other farm than own…………………………………………….3<br>Agriculture - own farm………………………4<br>Self-employed/run own business…………5<br>Petty trade……………………………………6<br>Unemployed…………………………………7<br>Contributing family workers……………….8<br>Attends to home chores…………………..9<br>Student………………………………………10<br>Retired……………………………………….11<br>Too young to work……………………..….12<br>Other………………………..……………96	
**HH_32o**	If other, please specify		
**HH_33**	What is your partner/husband occupation?	Employed in private sector……………….1<br>Government employee/civil service…….2<br>Agriculture - works on other farm than own…………………………………………….3<br>Agriculture - own farm………………………4<br>Self-employed/run own business…………5<br>Petty trade……………………………………6<br>Unemployed…………………………………7<br>Contributing family workers……………….8<br>Attends to home chores…………………..9<br>Student………………………………………10<br>Retired……………………………………….11<br>Too young to work……………………..….12<br>Other………………………..……………96	
**HH_33o**	If other, please specify		

Supplement to: Worku AG, Midekssa WD, Tilahun HA, et al. The impact of health information system interventions on maternal and child health service utilizations in Ethiopia: quasi-experimental study. *Glob Health Sci Pract*. 2024;12(6):2400145. https://doi.org/10.9745/GHSP-D-24-00145

**Pregnancy, Delivery and Postpartum Care Experience**			
Now I would like to ask you some questions about the time before, during, and after delivery.			
M_01	At the time you became pregnant with (NAME), was the pregnancy, wanted, mistimed or unwanted?	1. Wanted<br>2. Wanted but mistimed<br>3. Unwanted	
**Focused antenatal care**			
M_02	When you were pregnant with (BABY'S NAME), did you attend antenatal care?	0. No<br>1. Yes	If Yes to M_04
M_03	Why did you not attend antenatal care during this pregnancy?<br><br>**Probe and record all reasons mentioned**	Respondent didn't think necessary…1<br>Husband/family didn't think necessary……………………..……………2<br>Facility too far…………………………..3<br>No transport……………………………4<br>No childcare……………………………5<br>Too expensive…………………………6<br>Services are poor………………………7<br>Used home remedy……………………8<br>Did not know where to go…………9<br>No time to go…………………………10<br>Did not have money…………………11<br>Other……………………………………96<br>Don't know……………………………..98	
M_03o	If other, please specify		
M_04	How many times in total did you receive antenatal care during this pregnancy?<br><br>(Enter 98 if unknown)		

Supplement to: Worku AG, Mideksa WD, Tilahun HA, et al. The impact of health information system interventions on maternal and child health service utilizations in Ethiopia: quasi-experimental study. *Glob Health Sci Pract*. 2024;12(6):2400145. https://doi.org/10.9745/GHSP-D-24-00145

M_05	Where did you receive antenatal care for this pregnancy?	1. Home<br>2. Gvt. Hospital<br>3. Gvt. Health center<br>4. Gvt. Health post<br>5. Private clinic<br>96. Other			
M_05o	If other, please specify				
M_06	How many months pregnant were you when you went for your first antenatal care visit?<br><br>(Enter 98 if unknown)				
M_07	Whom did you see on your first visit?	1. Doctor<br>2. Health officer<br>3. Nurse/midwife<br>4. Health extension workers<br>Other(specify)_____________<br>98. Don't know			
M_08	How many months pregnant were you when you last received antenatal care for this pregnancy?<br><br>(Enter 98 if unknown)				
M_09	Whom did you see on your last visit?	1. Doctor<br>2. Health officer<br>3. Nurse/midwife<br>4. Health extension workers<br>Other(specify)_____________<br>98. Don't know			
M_09o	If other, please specify				
I would now like you to think about ALL of your visits when you were pregnant with (BABY'S NAME). During any of these visits, did the staff...					
		**No(0)**	**Yes(1)**	**DN(98)**	
M_10	Measure blood pressure				
M_11	Measure weight				
M_12	Take a blood sample?				

Supplement to: Worku AG, Midekssa WD, Tilahun HA, et al. The impact of health information system interventions on maternal and child health service utilizations in Ethiopia: quasi-experimental study. *Glob Health Sci Pract*. 2024;12(6):2400145. https://doi.org/10.9745/GHSP-D-24-00145

M_13	Take a urine sample?				
M_14	Give you a TT (tetanus) immunization?				
M_15	Give you information or advice about diet and nutrition during pregnancy?				
M_16	Tell your expected due date?				
M_17	Give you an update on how the baby is growing?				
M_18	Discuss the importance of planning or preparing for delivery?				
M_19	Advise you to give birth in a health facility?				
M_20	Inform you of danger signs during pregnancy?				
M_21	I don't want to know the results, but were you tested for the AIDS virus as part of your antenatal care?				
M_22	I don't want to know the results, but did you get the results of the test?				
M_23	During this pregnancy, were you given or did you buy any iron tablets or iron syrup?<br>Note: SHOW sample of TABLETS/SYRUP for the mother	0. No<br>1. Yes<br>98. Don't know			
M_24	During the whole pregnancy, for approximately how many days did you take the tablets or syrup?				
**Delivery care**					
M_25	How many months of gestation were you when (child's NAME) was born?<br>*(Enter 98 if unknown)*				
M_26	Was the birth weight of (BABY'S NAME) recorded?	0. No<br>1. Yes<br>98. Don't know			

Supplement to: Worku AG, Midekssa WD, Tilahun HA, et al. The impact of health information system interventions on maternal and child health service utilizations in Ethiopia: quasi-experimental study. *Glob Health Sci Pract*. 2024;12(6):2400145. https://doi.org/10.9745/GHSP-D-24-00145

M_27	Who assisted with the delivery of (BABY'S NAME)?<br><br>Anyone else?	1. Doctor<br>2. Health officer<br>3. Nurse/midwife<br>4. TBA<br>5. Health extension worker<br>6. Relative/friend<br>96. Others (specify)______________<br><br>98. Don't know/can't remember	
M_27o	If other, please specify		
M_28	Where did you delivered (BABY'S NAME)?<br><br>NOTE: if delivery occurred on the way to the health facility, consider as "delivered at home or in community".	1. Delivered at a health facility<br>2. Delivered at home or in community	
M_29	Why did you deliver at home/community?<br><br>**Probe for the reasons and record all mentioned.**	0. Respondent didn't think necessary<br>1. Husband/family didn't think necessary<br>2. Facility too far<br>3. No transport<br>4. No childcare<br>5. Too expensive<br>6. Services are poor<br>7. Used home remedy<br>8. Did not know where to go<br>9. No time to go<br>10. Did not have any money<br>96. Other<br><br>98. Don't know	
M_29o	If other, please specify		
M_30	Was (NAME) delivered by caesarean, that is, did they cut your belly open to take the baby out?	0. No<br>1. Yes	
M_31	What type of transport did you mainly use to get to the facility?<br><br>RECORD ONE RESPONSE ONLY.	1. Car<br>2. Motorbike<br>3. Public bus<br>4. Cart<br>5. Ambulance<br>6. On foot<br>96. Other<br><br>98. Don't know	
M_31o	If other, please specify		
M_32	Is there any payment for your delivery at health facility?	0. No, it is free<br>1. Yes, there was payment	

Supplement to: Worku AG, Midekssa WD, Tilahun HA, et al. The impact of health information system interventions on maternal and child health service utilizations in Ethiopia: quasi-experimental study. *Glob Health Sci Pract*. 2024;12(6):2400145. https://doi.org/10.9745/GHSP-D-24-00145

M_33	Expenses for delivery care at health facility		Amount	Don't know(98)	
		Transport cost			
		To purchase supplies			
		Payment for service			
		Payment for laboratory			
		Bed stay			
		Others			
		Total			
M_34	Do you think that the total amount you paid for the delivery was expensive, an appropriate amount, or inexpensive?		1. Expensive<br>2. Appropriate<br>3. Inexpensive		
**Postnatal check up**					
M_35	After (BABY'S NAME) was born, did skilled health workers check on yours and your baby's health?<br><br>*Tell the respondent about the meaning of skilled health workers*		0. No<br>1. Yes<br>98.Don't know		
M_36	If 'YES' , How long after delivery did the first check take place?<br><br>If less than one day, record hours. If less than one week, record days		1. In first 1-2 hours of delivery<br>2. Within first 24 hours of delivery<br>3. Within 72 hours of delivery<br>4. Within one week of delivery<br>5. In 1-2 weeks<br>6. Within 2-4 weeks<br>7. More than 1 month after delivery<br>8. Never checked<br>98. Don't know		
M_37	Where did this first check take place?		1. Home<br>2. Gvt. Hospital<br>3. Gvt. Health center<br>4. Gvt. Health post<br>5. Private clinic<br>Other (specify)_______________		
M_37o	If other, please specify				

M_38	Have you faced any complication related to this pregnancy and delivery?	0. No complication<br>1. Had a complication	
M_39	What complications did you experience?	1. Bleeding<br>2. Swelling of body<br>3. Convulsion<br>4. Prolonged labor<br>5. Fever (sepsis)<br>6. Leakage of urine or stool (fistula)<br>96. Others	
M_39o	If other, please specify		
M_40	Did you seek any assistance for this problem?	0. No<br>1. Yes	
M_41	IF YES: Which facility did you go to first?	1. Gvt. Hospital<br>2. Gvt. Health center<br>3. Gvt. Health post<br>4. Private clinic<br>96 Other	
M_41o	If other, please specify		
M_42	What kind of transport did you mainly use to get to the facility?<br><br>**Record one response only.**	1. Car<br>2. Motorbike<br>3. Public bus<br>4. Cart<br>5. Ambulance<br>6. On foot<br>96 Other	
M-42o	If other, please specify		

CHILD IMMUNIZATION CARD			
	The following questions related to child health and immunization. Eligible participants are women/caregivers with children between the ages or 12-23 months. Prior to beginning this section, ask respondent to fetch the child's immunization card, if available.<br><br>**Now I will ask you questions regarding vaccination practices and the health of your child.**		
**IMM_01**	Do you have a card where the child's vaccinations are written down? This could be their {name of card in Ethiopia} or a notebook with your child's vaccinations written on it.	Yes, card/notebook available ……….1<br>Yes, but card/notebook not available…………………………………2<br>No card/ notebook……………………0	If "No card" or "card not unavailable" skip to IMM_03
**IMM_02**	What is the vaccination source document presented?<br>*(Do not read aloud, observe which document is presented)*	Children's Clinic Card……………………1<br>Vaccination Exercise book…..…………..2<br>Other………………………………………..96	
**IMM_02o**	If other, please specify		
**IMM_03**	Why don't you have the vaccination card?	Misplaced/lost card…………..….….……1	

Supplement to: Worku AG, Mideksa WD, Tilahun HA, et al. The impact of health information system interventions on maternal and child health service utilizations in Ethiopia: quasi-experimental study. *Glob Health Sci Pract*. 2024;12(6):2400145. https://doi.org/10.9745/GHSP-D-24-00145

		Destroyed card……………….…...………2<br>Never received card…………………..….3<br>Card in home, but inaccessible (i.e. locked away) ……………………….……4<br>Card not available or outside of home……………………………….…….5<br>Card kept at health facility……………....6<br>Other……………………………….……….96	
**IMM_03o**	If other, please specify		
**IMM_04**	Has {childname} ever received any vaccinations, drops, or injections in the past?	Yes……………………..…………………..1<br>No……………………….……………………0<br>Don't know……………..…………………98	If no card available to IMM_01 and "No" or "don't know" to IMM_04 skip to end
**note**	Ask respondent for the vaccination card or exercise book and explain you would like to take a photo of it. Take a photo of the Immunization Record page of the Vaccination card or vaccination exercise book where all vaccinations are recorded.		
**IMM_05**	Take photo of vaccination card		
**BCG VACCINE**			
**IMM_06**	According to the vaccination card/exercise book did the child receive the BCG vaccine?<br>*(Enumerator do not read aloud, refer to card; receipt of vaccination can be indicated by date, tick, etc.)*	Yes…………………………………………..1<br>No……………………………………………0	If "No" skip to IMM_08
**IMM_07**	Is there a date written for BCG vaccination on the card/book?	Yes…………………………………………..1<br>No……………………………………………0	If "No" skip to IMM_08
**IMM_07a**	Enter date from vaccination card/book for BCG vaccine:		
**IMM_08**	Has {childname} ever received an injection in the left forearm that usually causes a scar?	Yes…………………………………………..1<br>No……………………………………………0<br>Don't know……………………….…..98	
**IMM_09**	If the child is present, check for evidence of a scar and record:<br>*(Look at child's left forearm)*	Scar present……………………………1<br>No scar present………………………..2<br>Child not available to check…………3	
**OPV (POLIO) VACCINE**			
**IMM_10**	According to the vaccination card/exercise book, did the child receive a vaccination for OPV 0?	Yes…………………………………………..1<br>No……………………………………………0	If "No" skip to IMM_12

	(Enumerator do not read aloud, refer to card; receipt of vaccination can be indicated by date, tick, etc.)		
**IMM_11**	Is there a date written for OPV 0 vaccination on the card/book?	Yes……………………………………………1<br>No……………………………………………0	If "No" skip to IMM_12
**IMM_11a**	Enter date from vaccination card/book for OPV 0:		
**IMM_12**	According to the vaccination card/exercise book, did the child receive a vaccination for OPV 1?<br>*(Enumerator do not read aloud, refer to card; receipt of vaccination can be indicated by date, tick, etc.)*	Yes……………………………………………1<br>No……………………………………………0	If "No" skip to IMM_14
**IMM_13**	Is there a date written for OPV 1 vaccination on the card/book?	Yes……………………………………………1<br>No……………………………………………0	If "No" skip to IMM_14
**IMM_13a**	Enter date from vaccination card/book for OPV 1:		
**IMM_14**	According to the vaccination card/exercise book, did the child receive a vaccination for OPV 2?<br>*(Enumerator do not read aloud, refer to card; receipt of vaccination can be indicated by date, tick, etc.)*	Yes……………………………………………1<br>No……………………………………………0	If "No" skip to IMM_16
**IMM_15**	Is there a date written for OPV 2 vaccination on the card/book?	Yes……………………………………………1<br>No……………………………………………0	If "No" skip to IMM_16
**IMM_15a**	Enter date from vaccination card/book for OPV 2:		
**IMM_16**	According to the vaccination card/exercise book, did the child receive a vaccination for OPV 3?<br>*(Enumerator do not read aloud, refer to card; receipt of vaccination can be indicated by date, tick, etc.)*	Yes……………………………………………1<br>No……………………………………………0	If "No" skip to IMM_18
**IMM_17**	Is there a date written for OPV 3 vaccination on the card/book?	Yes……………………………………………1<br>No……………………………………………0	If "No" skip to IMM_18
**IMM_17a**	Enter date from vaccination card/book for OPV 3:		
**IMM_18**	According to the vaccination card/exercise book, did the child receive a vaccination for OPV 4?<br>*(Enumerator do not read aloud, refer to card; receipt of vaccination can be indicated by date, tick, etc.)*	Yes……………………………………………1<br>No……………………………………………0	If "No" skip to IMM_20<br><br>Relevant if IMM_10 is "no".

**IMM_19**	Is there a date written for OPV 4 vaccination on the card/book?	Yes…………………………………………..1<br>No……………………………………………0	If "No" skip to IMM_20
**IMM_19a**	Enter date from vaccination card/book for OPV 4:		
**IMM_20**	Has {childname} ever received oral polio drops in the mouth?	Yes…………………………………………..1<br>No……………………………………………0<br>Don't know…………………………..….98	If "No" or "don't know" skip to IMM_24
**IMM_21**	Was the first Polio vaccine given to the child within two weeks of birth?	Yes…………………………………………..1<br>No……………………………………………0<br>Don't know…………………………..….98	
**IMM_22**	How many times in total did {childname} receive the Polio vaccine at the health facility or as part of their normal schedule of vaccines?	Zero………………………………………..0<br>One…………………………………………1<br>Two…………………………………………2<br>Three……………………………………...3<br>Four…………………………………..……..4<br>More than four…………………………..5<br>Don't know……………………………..98	
**IMM_23**	How many times was the Polio vaccine given during a large campaign, usually involving a large group of children where the child's finger is painted after receiving the vaccine?	Zero………………………………………..0<br>One…………………………………………1<br>Two…………………………………………2<br>Three……………………………………...3<br>Four…………………………………..……..4<br>More than four…………………………..5<br>Don't know……………………………..98	
**DPT-HepB-Hib VACCINE (Pentavalent Vaccine)**			
**IMM_24**	According to the vaccination card/exercise book, did the child receive a vaccination for DPT-HepB-Hib1?<br>*(Enumerator do not read aloud, refer to card; receipt of vaccination can be indicated by date, tick, etc.)*	Yes…………………………………………..1<br>No……………………………………………0	If "No" skip to IMM_26
**IMM_25**	Is there a date written for DPT-HepB-Hib1 vaccination on the card/book?	Yes…………………………………………..1<br>No……………………………………………0	If "No" skip to IMM_26
**IMM_25a**	Enter date from vaccination card/book for DPT-HepB-Hib1:		
**IMM_26**	According to the vaccination card/exercise book, did the child receive a vaccination for DPT-HepB-Hib2?	Yes…………………………………………..1<br>No……………………………………………0	If "No" skip to IMM_28

Supplement to: Worku AG, Midekssa WD, Tilahun HA, et al. The impact of health information system interventions on maternal and child health service utilizations in Ethiopia: quasi-experimental study. *Glob Health Sci Pract*. 2024;12(6):2400145. https://doi.org/10.9745/GHSP-D-24-00145

	(Enumerator do not read aloud, refer to card; receipt of vaccination can be indicated by date, tick, etc.)		
**IMM_27**	Is there a date written for DPT-HepB-Hib2 vaccination on the card/book?	Yes……………………………………………1<br>No……………………………………………0	If "No" skip to IMM_28
**IMM_27a**	Enter date from vaccination card/book for DPT-HepB-Hib2:		
**IMM_28**	According to the vaccination card/exercise book, did the child receive a vaccination for DPT-HepB-Hib3?<br>*(Enumerator do not read aloud, refer to card; receipt of vaccination can be indicated by date, tick, etc.)*	Yes……………………………………………1<br>No……………………………………………0	If "No" skip to IMM_30
**IMM_29**	Is there a date written for DPT-HepB-Hib3 vaccination on the card/book?	Yes……………………………………………1<br>No……………………………………………0	If "No" skip to IMM_30
**IMM_29a**	Enter date from vaccination card/book for DPT-HepB-Hib3:		
**IMM_30**	Has {childname} ever received an injection on the left upper outer thigh?<br>*Inform caregiver this is the DPT or Penta vaccination.*	Yes……………………………………………1<br>No……………………………………………0<br>Don't know………………………………98	If "No" or "Don't know" skip to IMM_32
**IMM_31**	How many times has the DPT or Penta injection on the left upper outer thigh been given?	One……………………………………………1<br>Two……………………………………………2<br>Three…………………………………………3<br>More than three…………………………4<br>Don't know………………………………98	
**PCV (PNEUMOCOCCAL VACCINE)**			
**IMM_32**	According to the vaccination card/exercise book, did the child receive a vaccination for PCV1?<br>*(Enumerator do not read aloud, refer to card; receipt of vaccination can be indicated by date, tick, etc.)*	Yes……………………………………………1<br>No……………………………………………0	If "No" skip to IMM_34
**IMM_33**	Is there a date written for PCV1 vaccination on the card/book?	Yes……………………………………………1<br>No……………………………………………0	If "No" skip to IMM_34
**IMM_33a**	Enter date from vaccination card/book for PCV1:		
**IMM_34**	According to the vaccination card/exercise book, did the child receive a vaccination for PCV2?<br>*(Enumerator do not read aloud, refer to card; receipt of vaccination can be indicated by date, tick, etc.)*	Yes……………………………………………1<br>No……………………………………………0	If "No" skip to IMM_36

Supplement to: Worku AG, Midekssa WD, Tilahun HA, et al. The impact of health information system interventions on maternal and child health service utilizations in Ethiopia: quasi-experimental study. *Glob Health Sci Pract*. 2024;12(6):2400145. https://doi.org/10.9745/GHSP-D-24-00145

**IMM_35**	Is there a date written for PCV2 vaccination on the card/book?	Yes……………………………………………1<br>No……………………………………………0	If "No" skip to IMM_36
**IMM_35a**	Enter date from vaccination card/book for PCV2:		
**IMM_36**	According to the vaccination card/exercise book, did the child receive a vaccination for PCV3?<br>*(Enumerator do not read aloud, refer to card; receipt of vaccination can be indicated by date, tick, etc.)*	Yes……………………………………………1<br>No……………………………………………0	If "No" skip to IMM_38
**IMM_37**	Is there a date written for PCV3 vaccination on the card/book?	Yes……………………………………………1<br>No……………………………………………0	If "No" skip to IMM_38
**IMM_37a**	Enter date from vaccination card/book for PCV3:		
**IMM_38**	Has {childname} ever received an injection on the right upper outer thigh?<br>*Inform the caregiver this is known as the PCV or Pneumo vaccine*	Yes……………………………………………1<br>No……………………………………………0<br>Don't know…………………………….…..98	If "No" or "Don't know" skip to IMM_40
**IMM_39**	How many times has the PCV injection on the right upper outer thigh been given?	One…………………………………………1<br>Two…………………………………………2<br>Three………………………………………..3<br>More than three…………………………4<br>Don't know……………………………….98	
**MEASLES/MEASLES RUBELLA (MR)**			
**IMM_40**	According to the vaccination card/exercise book, did the child receive a vaccination for Measles or MR 1?<br>*(Enumerator do not read aloud, refer to card; receipt of vaccination can be indicated by date, tick, etc.)*	Yes……………………………………………1<br>No……………………………………………0	If "No" skip to IMM_42
**IMM_41**	Is there a date written for Measles/MR 1 vaccination on the card/book?	Yes……………………………………………1<br>No……………………………………………0	If "No" skip to IMM_42
**IMM_41a**	Enter date from vaccination card/book for Measles/MR 1:		
**IMM_42**	According to the vaccination card/exercise book, did the child receive a vaccination for Measles or MR 2?<br>*(Enumerator do not read aloud, refer to card; receipt of vaccination can be indicated by date, tick, etc.)*	Yes……………………………………………1<br>No……………………………………………0	If "No" skip to IMM_44
**IMM_43**	Is there a date written for Measles/MR 2 vaccination on the card/book?	Yes……………………………………………1<br>No……………………………………………0	If "No" skip to IMM_44

Supplement to: Worku AG, Midekssa WD, Tilahun HA, et al. The impact of health information system interventions on maternal and child health service utilizations in Ethiopia: quasi-experimental study. *Glob Health Sci Pract*. 2024;12(6):2400145. https://doi.org/10.9745/GHSP-D-24-00145

**IMM_43a**	Enter date from vaccination card/book for Measles/MR 2:		
**IMM_44**	Has {childname} ever received an injection on the left upper arm?<br>*Inform caregiver this is the measles vaccine.*	Yes…………………………………………..1<br>No……………………………………………0<br>Don't know……………………………..…98	If "No" or "Don't know" skip to IMM_47
**IMM_45**	How many times has the measles injection on the left upper arm been given?	One……………………………………………1<br>Two…………………………………………..2<br>More than two times……………………..3<br>Don't know………………………………98	
**IMM_46**	How many times was the measles injection given during a large campaign, normally involving a large group of children up to 5 years of age?	Zero……………………………………………0<br>One……………………………………………1<br>Two…………………………………………..2<br>More than two times……………………..3<br>Don't know………………………………98	
**ROTAVIRUS VACCINE**			
**IMM_47**	According to the vaccination card/exercise book, did the child receive a vaccination for Rota vaccine 1?<br>*(Enumerator do not read aloud, refer to card; receipt of vaccination can be indicated by date, tick, etc.)*	Yes…………………………………………..1<br>No……………………………………………0	If "No" skip to IMM_49
**IMM_48**	Is there a date written for Rota vaccine 1 vaccination on the card/book?	Yes…………………………………………..1<br>No……………………………………………0	If "No" skip to IMM_49
**IMM_48a**	Enter date from vaccination card/book for Rota vaccine 1:		
**IMM_49**	According to the vaccination card/exercise book, did the child receive a vaccination for Rota vaccine 2?<br>*(Enumerator do not read aloud, refer to card; receipt of vaccination can be indicated by date, tick, etc.)*	Yes…………………………………………..1<br>No……………………………………………0	If "No" skip to IMM_51
**IMM_50**	Is there a date written for Rota vaccine 2 vaccination on the card/book?	Yes…………………………………………..1<br>No……………………………………………0	If "No" skip to IMM_51
**IMM_50a**	Enter date from vaccination card/book for Rota vaccine 2:		
**IMM_51**	Has {childname} ever been administered a vaccination orally (in mouth) using a syringe to prevent diarrhea?<br>*Inform caregiver this is the Rotavirus vaccine.*	Yes…………………………………………..1<br>No……………………………………………0<br>Don't know……………………………..…98	If "No" or "Don't know" skip to IMM_53
**IMM_52**	How many times has {childname} received the Rotavirus vaccination in the mouth?	One……………………………………………1<br>Two…………………………………………..2	

Supplement to: Worku AG, Midekssa WD, Tilahun HA, et al. The impact of health information system interventions on maternal and child health service utilizations in Ethiopia: quasi-experimental study. *Glob Health Sci Pract*. 2024;12(6):2400145. https://doi.org/10.9745/GHSP-D-24-00145

		More than two……………………………3<br>Don't know………………………………98	
**VITAMIN A**			
**IMM_53**	According to the vaccination card or exercise book, did the child receive vitamin A?<br>*(Enumerator do not read aloud, refer to card; receipt of vaccination can be indicated by date, tick, etc.)*<br><br>*SHOW COMMON TYPES OF MPULES/CAPSULES/SYRUPS*	Yes…………………………………………...1<br>No……………………………………………0	If "No" skip to IMM_56
**IMM_54**	Is there a date written for vitamin A on the card/book?	Yes…………………………………………...1<br>No……………………………………………0	If "No" skip to IMM_56
**IMM_55**	Enter date from vaccination card/book for vitamin A:		
**IMM_56**	Has {childname} ever been administered a vaccination orally (in mouth) using a…?<br>*Inform caregiver this is Vitamin A.*	Yes…………………………………………...1<br>No……………………………………………0<br>Don't know…………………………….…98	

**FAMILY PLANNING**			
FP_01	Are you pregnant now?	0. No<br>1. Yes<br>98. Don't know	No/Don't Know, skip to FP_04
FP_02	How many months pregnant are you?<br>RECORD NUMBER OF COMPLETED MONTHS.	Months: ___/___	
FP_03	Is the pregnancy wanted, mistimed or unwanted?	1. Wanted<br>2. Wanted but mistimed<br>3. Unwanted	
FP_04	Have you ever heard of the various ways or methods that a couple can use to delay or avoid a pregnancy? Mention the methods<br><br>Don't read the lists to the respondent. Select all that apply	Female Sterilization……………1<br>Male Sterilization……………….2<br>IUD……………………………….3<br>Injectables……………………….4<br>Implants………………………….5<br>Pill………………………………….6<br>Condom………………………….7<br>Female condom……………….8<br>Emergency contraception….9<br>Standard days method……..10	

Supplement to: Worku AG, Midekssa WD, Tilahun HA, et al. The impact of health information system interventions on maternal and child health service utilizations in Ethiopia: quasi-experimental study. *Glob Health Sci Pract*. 2024;12(6):2400145. https://doi.org/10.9745/GHSP-D-24-00145

		Lactational amenorrhea method…11<br>Rhythm method………………12<br>Withdrawal……………… ……..13	
FP_05	Are you or your partner currently doing something or using any method to delay or avoid getting pregnant?	0. No<br>1. Yes	*If No, skip to FP_8*
FP_06	What is the main method you are currently using?	1. Female Sterilization<br>2. Male Sterilization<br>3. IUD<br>4. Injectables<br>5. Implants<br>6. Pill<br>7. Condom<br>8. Female condom<br>9. Emergency contraception<br>10. Standard days method<br>11. Lactational amenorrhea method<br>12. Rhythm method<br>13. Withdrawal<br>96. Other modern method<br>98. Other traditional method	
FP_7	Where did you obtain this method the last time?	Home<br>1. Your home<br>2. Other home<br>Government health facility<br>3. Govt hospital<br>4. Govt health center<br>5. Govt health post<br>NGO health facility<br>6. Hospital/clinic<br>Private health facility<br>7. Private Hospital<br>8. Private Clinic<br>Other sources<br>9. Retail store<br>10. Friends/Relatives<br>Other(specify) --------------	
FP_8	What is your future plan regarding having children?	1. Wants to space<br>2. Wants no more children<br>3. Wants children soon	

Supplement to: Worku AG, Midekssa WD, Tilahun HA, et al. The impact of health information system interventions on maternal and child health service utilizations in Ethiopia: quasi-experimental study. *Glob Health Sci Pract*. 2024;12(6):2400145. https://doi.org/10.9745/GHSP-D-24-00145

	***You have reached the end of the survey*. Thank you very much for your valuable information!**

Facility Survey

Data use and data quality assessment tool: Woreda Level

Purpose

1. Identify RHIS data quality, disaggregated data, and information use issues.
2. Quantify the levels of data quality (accuracy, reporting timeliness, and completeness) and information use status (access to RHIS data, existence of analyzed data, and use of RHIS data for monitoring and planning).
3. Identify issues/problems with data processing and processes for information use.

Summary of Information Collected via the RHIS Performance Diagnostic Tool at the Woreda Level

Measuring Data Quality

Supplement to: Worku AG, Midekssa WD, Tilahun HA, et al. The impact of health information system interventions on maternal and child health service utilizations in Ethiopia: quasi-experimental study. *Glob Health Sci Pract*. 2024;12(6):2400145. https://doi.org/10.9745/GHSP-D-24-00145

Through an analysis of program indicators, the RHIS Performance Diagnostic Tool quantifies the status of data completeness, timeliness, and accuracy, and thus provides valuable information on the adequacy of health facility data to support planning and monitoring. The data quality assessment section of this tool is aligned with the data verification aspect of the Data Quality Review (DQR) Tool. At the Woreda level, the Tool compares reported data and the value entered in Woreda database for the same indicators and reporting period examined at the facility level. The RHIS Performance Diagnostic Tool has the following core recommended indicators to assess data quality:

S.N	Category	Indicators (data elements)	Government HC and above	HP	Private HF	Remark
1	Maternal health	Number of births attended by skilled attendants	√		√	
2	Family planning	Total number of family planning (new and repeat) acceptors by age	√	√	√	
3	Immunization	Number of children under one year of age who have received third dose of pentavalent (Penta3) vaccine	√	√	√	
4	HIV service	Number of clients tested HIV positive	√		√	
5	Disease	Number of under-five children with pneumonia	√	√	√	
		Confirmed malaria cases (microscopy or RDT)	√	√	√	
7	Service quality	Number of inpatient deaths	√		√*	
8	Tuberculosis	TB cases (all types)	√		√	

***Private Hospitals**

Measuring Information Use

The RHIS Performance Diagnostic Tool also measures the continuous use of information to guide day-to-day operations, track performance, learn from past results, and improve service delivery. The tool focuses on the use of RHIS data for analytic report production, discussion, decision/actions, target setting, planning, and monitoring.

Assessing RHIS Data Management Processes

This section assesses various aspects of RHIS data management processes, including:

- Data processing, analysis, and presentation: the availability of a copy of RHIS data management guidelines; use of standardized RHIS data collection and reporting tools; evidence of data analysis; and visual representation of data.
- Data quality check: presence of data quality assurance guidelines and tools; clearly assigned roles and responsibilities for data review; and regular internal data quality checks conducted by the health facility.

- Feedback: existence of formal feedback loops to the staff collecting the data; health facilities receive regular written feedback on their performance; and quality of reported data.
- Supervision quality: supervision frequency; checking data quality; using data for discussion; helping in decision making; and supervisory feedback.

Data Collection Method

- Key informant interviews (health facility in-charge and data manager, or those responsible for the compilation, reporting, and analysis of data)
- Document review and observation (HMIS reports, electronic database, planning documents, meeting minutes, feedback reports/notes, guidelines)

Supplement to: Worku AG, Midekssa WD, Tilahun HA, et al. The impact of health information system interventions on maternal and child health service utilizations in Ethiopia: quasi-experimental study. *Glob Health Sci Pract*. 2024;12(6):2400145. https://doi.org/10.9745/GHSP-D-24-00145

Part 1. Data Quality Assessment: Woreda Health Office Form

Number	Question	Result	Skip
**INTERVIEWER VISITS**			
**DQ_101**	Interview date		
**DQ_102**	Interviewer name		
**DQ_103**	Interviewer code<br>Please enter your 3-character identifier.		
**WOREDA LEVEL UNIT IDENTIFICATION**			
**DQ_104**	Please select the region	Addis Ababa<br>Afar<br>Amhara<br>Beneshangul Gumuz<br>Dire Dawa<br>Gambella<br>Harari<br>Oromiya<br>SNNPR<br>Somali<br>Tigray	
**DQ_105**	Zone/Sub-city<br>Please enter the alphanumeric code that identifies this level (up to 11 alphanumeric digits).		
**DQ_106**	Woreda<br>Please enter the alphanumeric code that identifies this level (up to 11 alphanumeric digits).		
**DQ_108**	Name of Woreda office(s) visited<br>**(NOTE: IT COULD BE ONE OR MORE OFFICES FROM WHICH INFORMATION IS COLLECTED. PLEASE LIST THEM HERE.)**	______<br>______<br>______	

**GENERAL INFORMATION**
**READ THE FOLLOWING STATEMENT TO THE MANAGER, THE HEAD OF THE WOREDA UNIT:**

Supplement to: Worku AG, Mideksa WD, Tilahun HA, et al. The impact of health information system interventions on maternal and child health service utilizations in Ethiopia: quasi-experimental study. *Glob Health Sci Pract*. 2024;12(6):2400145. https://doi.org/10.9745/GHSP-D-24-00145

Good day! My name is ____________________. We are here on behalf of [IMPLEMENTING AGENCY] conducting a survey of Woreda health offices to assist the government in knowing more about the performance of routine health information system [COUNTRY].

Your Woreda was randomly selected to participate in this study. We will be asking you questions about various health services and routine reporting. Information about your unit may be used by the MOH, organizations supporting health services, and researchers for planning service improvements or for conducting further studies on health services.

Neither your name nor those of any other respondents participating in this study will be included in the dataset or in any report; however, there is a small chance that any of the respondents may be identified later. We are asking for your help to ensure that the information we collect is accurate.

You may refuse to answer any question or choose to stop the interview at any time. However, we hope you will answer the questions, which will benefit the clients you serve, the nation, and yourself.

If there are questions for which someone else is the most appropriate person to provide the information, we would appreciate it if you would introduce us to that person to help us collect the information.

At this point, do you have any questions about the study? Do I have your agreement to proceed?

**DQ_109**	May I begin the interview?	YES ☐ 2. NO ☐

PLEASE ENTER THE 3 REVIEW MONTHS THAT WILL BE USED DURING THIS ASSESSMENT		
	Month_1	Meskerem 2012……1<br>Tikemet 2012……2<br>Hidar 2012……3<br>Tahesas 2012……4<br>Tir 2012……5<br>Yekatit 2012……6<br>Megabit 2012……7<br>Miyazia 2012……8<br>Ginbot 2012……9<br>Sene 2012……10<br>Hamle 2012……11<br>Nehase 2012……12
	Month_2	Meskerem 2012……1<br>Tikemet 2012……2<br>Hidar 2012……3<br>Tahesas 2012……4<br>Tir 2012……5<br>Yekatit 2012……6<br>Megabit 2012……7<br>Miyazia 2012……8<br>Ginbot 2012……9<br>Sene 2012……10<br>Hamle 2012……11<br>Nehase 2012……12
	Month_3	Meskerem 2012……1<br>Tikemet 2012……2

Supplement to: Worku AG, Midekssa WD, Tilahun HA, et al. The impact of health information system interventions on maternal and child health service utilizations in Ethiopia: quasi-experimental study. *Glob Health Sci Pract*. 2024;12(6):2400145. https://doi.org/10.9745/GHSP-D-24-00145

		Hidar 2012……………………………………………3<br>Tahesas 2012……………………………………….4<br>Tir 2012…………………………………………………5<br>Yekatit 2012……………………………………….6<br>Megabit 2012……………………………………..7<br>Miyazia 2012………………………………………8<br>Ginbot 2012……………………………………….9<br>Sene 2012…………………………………………..10<br>Hamle 2012………………………………………..11<br>Nehase 2012……………………………………….12

RESOURCES FOR DATA ASSESSMENT			
DQ_010	Does the Woreda have a designated person responsible for entering data/compiling reports from health facilities?	1. Yes ☐	0. No ☐
DQ_011	Does the Woreda have a designated person or group of people (e.g. PMT)to review the quality of compiled data prior to submission to the next level?	Yes ________________________1<br>No__________________________0	
DQ_012	Does the Woreda have the following guidelines:<br>(Please observe.)		
	A. Data entry/compilation (DHIS2 user manual)	1. Yes ☐	0. No ☐
	B. Data quality and Information use guide (for data quality review and control)	1. Yes ☐	0. No ☐
DQ_013	If yes to DQ_011, are the members of PMT trained on:		
	A. HMIS related data entry/compilation	Yes (all staff have received training in the past 12 months)______________________1<br>Mostly (all staff have received training but not in the past 12 months)_______________2<br>Partly (some staff have received training in the past 12 months)____3<br>Not at all_____________________0	
	B. HMIS related data review and quality control?	Yes (All staff have received training in the past 12 months) ______________________1<br>Mostly (all staff have received training but not in the past 12 months)_______________2<br>Partly (some staff have received training in the past 12 months)____3<br>Not at all_____________________0	

COMPLETENESS OF HEALTH FACILITIES REPORTING TO WOREDA

Supplement to: Worku AG, Midekssa WD, Tilahun HA, et al. The impact of health information system interventions on maternal and child health service utilizations in Ethiopia: quasi-experimental study. *Glob Health Sci Pract*. 2024;12(6):2400145. https://doi.org/10.9745/GHSP-D-24-00145

DQ_014a	Does the woreda keep log book/electronic system to track monthly HMIS reports sent by the health facilities? (observe)	Yes, paper-based only........1<br>Yes, electronic only........2<br>Yes, both paper-based and electronic systems...3<br>No........0
DQ_014b	Does the Woreda keep copies of monthly HMIS reports (paper-based or electronic) sent by the health facilities? (observe)<br>**(Check the reports from month 1 to month 3)**	Yes, paper-based only........1<br>Yes, electronic only........2<br>Yes, both paper-based and electronic copies (all health facilities submit both types of reports)…3<br>Yes, mixed (some health facilities submit paper-based reports; others submit electronic reports)..4<br>No........0

DQ_015

How many health facilities in the Woreda are supposed to submit the monthly HMIS report (service and disease) to the Woreda and by what method?

**Health facility type*** **(*Specify the facility type according to the structure of the country's health system.)**	**A. Paper-based report only**				**B. Electronic report only**				**C. Both paper and electronic reports**			
	**Service**	**IPD**	**OPD**	**Quarterly**	**Service**	**IPD**	**OPD**	**Quarterly**	**Service**	**IPD**	**OPD**	**Quarterly**
01. Primary Hospital (												
02. Health centers												
03. Health posts												
04. Private/NGO/faith based health facilities												

DQ_016

CHECK THE MONTHLY HMIS REPORTS SUBMITTED BY THE HEALTH FACILITIES DURING THE REVIEW PERIOD

How many health facilities in the Woreda actually submitted monthly HMIS (service and disease) reports for:

A. Month 1 __________ year____________?

**Health facility type*** **(*Specify the facility type according to the structure of the country's health system.)**	**A. Paper-based report only**			**B. Electronic report only**			**C. Both paper and electronic reports**		
	**Service**	**IPD**	**OPD**	**Service**	**IPD**	**OPD**	**Service**	**IPD**	**OPD**
01. Primary Hospital									
02. Health centers									
03. Health posts									
04. Private/NGO/faith based health facilities									

Supplement to: Worku AG, Midekssa WD, Tilahun HA, et al. The impact of health information system interventions on maternal and child health service utilizations in Ethiopia: quasi-experimental study. *Glob Health Sci Pract*. 2024;12(6):2400145. https://doi.org/10.9745/GHSP-D-24-00145

B. Month 2 __________ year____________?

Health facility type* (*Specify the facility type according to the structure of the country's health system.)	A. Paper-based report only			B. Electronic report only			C. Both paper and electronic reports		
	Service	IPD	OPD	Service	IPD	OPD	Service	IPD	OPD
01. Primary Hospital									
02. Health centers									
03. Health posts									
04. Private/NGO/faith based health facilities									

C. Month 3 __________ year____________?

Health facility type* (*Specify the facility type according to the structure of the country's health system.)	A. Paper-based report only				B. Electronic report only				C. Both paper and electronic reports			
	Service	IPD	OPD	Quarter	Service	IPD	OPD	Quarter	Service	IPD	OPD	Quarter
01. Primary Hospital												
02. Health centers												
03. Health posts												
04. Private/NGO/faith based health facilities												

DQ_017	If health facilities are not submitting monthly HMIS reports, what are the possible reasons for this?	Staffing issue(s)........1<br>Lack of reporting supplies........2<br>Transportation issue(s)........3<br>Internet connectivity issue(s)........4<br>Presence of other vertical reporting requirements 5<br>Computer system failure........6<br>Electric supply interruption........7<br><br>Other (specify) ____________96

Supplement to: Worku AG, Mideksssa WD, Tilahun HA, et al. The impact of health information system interventions on maternal and child health service utilizations in Ethiopia: quasi-experimental study. *Glob Health Sci Pract*. 2024;12(6):2400145. https://doi.org/10.9745/GHSP-D-24-00145

REPORTED DATA COMPLETENESS ON SELECTED INDICATORS									
**PLEASE COMPLETE THE FOLLOWING FOR EACH OF THE SELECTED INDICATORS.**		# of skilled births	# of FP acceptors	# of Penta3	# of HIV positive clients	# of conf. malaria cases	# of U5 children pneumonia	# of inpatient deaths	# of TB cases notified
DQ_018a	How many facilities were expected to report on the selected indicators?	**A**	**B**	**C**	**D**	**E**	**F**	**G**	**H**
	01. Month 1								
	02. Month 2								
	03. Month 3								
DQ_018b	How many facilities actually reported on the selected indicators? (Observe)	**A**	**B**	**C**	**D**	**E**	**F**	**G**	**H**
	01. Month 1								
	02. Month 2								
	03. Month 3								
DQ_019	How many reports were complete? (Complete means the report contains the data relevant to the selected indicators including all required data disaggregation.)	**A**	**B**	**C**	**D**	**E**	**F**	**G**	**H**
	01. Month 1								
	02. Month 2								
	03. Month 3								
DQ_020	If any monthly HMIS reports were not complete, what are the possible reasons for the missing data?		Staffing issue(s)(e.g. staff shortage, absence of designated staff, etc)........1<br>Not understanding the data element(s) ....2<br>Presence of other vertical reporting requirements ....3<br>Data burden (too much data elements to be recorded)........4<br>The design of the reporting form is not user friendly........5<br>Other (specify)________96						

Supplement to: Worku AG, Midekssa WD, Tilahun HA, et al. The impact of health information system interventions on maternal and child health service utilizations in Ethiopia: quasi-experimental study. *Glob Health Sci Pract*. 2024;12(6):2400145. https://doi.org/10.9745/GHSP-D-24-00145

REPORT TIMELINESS

DQ_021	A. Is there a deadline for submission of the monthly HMIS report by the health facilities?	1. Yes ☐	0. No ☐	➔DQ_024 (if No)
	B. If yes, what is the deadline (date of month)? (NB:Write the end date of the deadline)	Reporting deadline: ______________________		
DQ_022	Does the Woreda office record receipt dates of monthly HMIS reports (observe logbook/electronic system)?	1. Yes ☐	0. No ☐	➔DQ_024 (if No)
DQ_023	If DQ_022 is yes, check the receipt dates for the three review months. How many reports were received on or before the 26th of the month<br>*Note for enumerators: Refer the reporting timeline for each level from the Interview guide.*			

Health facility type* (*Specify the facility type according to the structure of the country's health system.)	A. Month 1			B. Month 2			C. Month 3			
	Service	IPD	OPD	Service	IPD	OPD	Service	IPD	OPD	Quarter
01. Primary Hospital										
02. Health centers										
03. Health posts										
04. Private/NGO/faith based health facilities										

DQ_024	Does the Woreda office keep a record of its submission of monthly aggregated HMIS reports to Zonal or regional offices (e.g. emails, stamps, receipts, log book, etc.)?	1. Yes ☐	0. No ☐	➔DQ_026 (if No)
DQ_025a	If DQ_024 is yes, check the submission dates of the aggregate HMIS reports for the three review months.			
		**Service**	**IPD**	**OPD**
	Which of the monthly HMIS reports are submitted on time to Zone/Region in **Month 1**?	1. Yes ☐<br>0. No ☐	1. Yes ☐<br>0. No ☐	1. Yes ☐<br>0. No ☐
DQ_025b	Which of the monthly HMIS reports are submitted on time to Zone/Region in **Month 2**?	1. Yes ☐<br>0. No ☐	1. Yes ☐<br>0. No ☐	1. Yes ☐<br>0. No ☐
DQ_025c	Which of the monthly HMIS reports are submitted on time to Zone/Region in **Month 3**?	1. Yes ☐<br>0. No ☐	1. Yes ☐<br>0. No ☐	1. Yes ☐<br>0. No ☐

Supplement to: Worku AG, Mideksssa WD, Tilahun HA, et al. The impact of health information system interventions on maternal and child health service utilizations in Ethiopia: quasi-experimental study. *Glob Health Sci Pract*. 2024;12(6):2400145. https://doi.org/10.9745/GHSP-D-24-00145

DATA ACCURACY

MANUALLY RE-AGGREGATE THE REPORTED FIGURES FOR THE FOLLOWING INDICATORS FROM THE HMIS MONTHLY REPORTS THAT ARE SUBMITTED BY THE VISITED HEALTH FACILITIES FOR THE 3 REVIEW MONTHS. COMPARE THE FIGURES WITH THE DATA IN THE WOREDA HEALTH OFFICE HMIS DATABASE.

Please use the data compilation sheet provided for re-aggregating the value of the selected indicators from those visited health facilities reporting to the Woreda Health Office.

In rear cases, if the Woreda Health Office HMIS database is not accessible during the field survey please complete this section by checking the HMIS database at the Zone Health Department.

	**Indicators**	Re-aggregated value from the monthly/quarterly reports submitted by those facilities visited<br><br>(if missing or not applicable, leave blank)<br><br>(A)	Reported data from Woreda's electronic database or paper-based reports, specific to the visited facilities<br><br>(if missing or not applicable, leave blank)<br><br>(B)	Reason for observed discrepancy (if A ≠ B) (list all that apply)<br>Data entry errors .... 1<br>Arithmetic errors .... 2<br>Information from submitted reports not compiled correctly .... 3<br>Monthly reports not available .... 4<br>Update made in the HF are not captured in the woreda .... 5<br>Asked to make changes by higher level .... 6<br>Other (specify) ________ 96<br><br>(C)
**DQ_026 Month 1: ____________**				
01	# of skilled births			
02	# of FP acceptors			
03	# of Penta3			
04	# of HIV positive clients			
05	# of confirmed malaria cases			
06	# of U5 children pneumonia			
07	# of inpatient deaths			
**DQ_027 Month 2: ____________**				
01	# of skilled births			
02	# of FP acceptors			
03	# of Penta3			
04	# of HIV positive clients			
05	# of confirmed malaria cases			
06	# of U5 children pneumonia			

Supplement to: Worku AG, Mideksa WD, Tilahun HA, et al. The impact of health information system interventions on maternal and child health service utilizations in Ethiopia: quasi-experimental study. *Glob Health Sci Pract*. 2024;12(6):2400145. https://doi.org/10.9745/GHSP-D-24-00145

07	# of inpatient deaths			
**DQ_028 Month 3: ______________________________**				
01	# of skilled births			
02	# of FP acceptors			
03	# of Penta3			
04	# of HIV positive clients			
05	# of confirmed malaria cases			
06	# of U5 children pneumonia			
07	# of inpatient deaths			
08	# of TB cases notified			

DATA ACCURACY

WRITE THE AGGREGATED TOTALS OF THE WOREDA HMIS REPORTS, EITHER ELECTRONIC OR PAPER-BASED, THAT ARE SUBMITTED BY THE WOREDA TO ZONAL/REGIONAL OFFICES.

If missing or not applicable, leave blank

**DQ_029**	**Indicators**	**Month 1**	**Month 2**	**Month 3**
01	# of skilled births			
02	# of FP acceptors			
03	# of Penta3			
04	# of HIV positive clients			
05	# of confirmed malaria cases			
06	# of U5 children pneumonia			
07	# of inpatient deaths			
08	# of TB cases notified			

Supplement to: Worku AG, Midekssa WD, Tilahun HA, et al. The impact of health information system interventions on maternal and child health service utilizations in Ethiopia: quasi-experimental study. *Glob Health Sci Pract*. 2024;12(6):2400145. https://doi.org/10.9745/GHSP-D-24-00145

DATA QUALITY ASSESSMENT MECHANISMS				
DQ_030	Has the Woreda conducted data quality assessments at all health facilities in the review three months? (Please observe)	1. Yes, RDQA has been conducted in all health centers and hospitals, and at least one health post under each health center ☐<br>2. RDQA has been conducted, but only in some facilities (Not conducted in all) ☐<br>0. No RDQA conducted ☐		
DQ_031	Does the Woreda use data quality assessment tools (e.g., RDQA/data verification, in-built electronic data quality validation rules/system)? (Please observe)	1. Yes, observed ☐	2. No ☐	
DQ_032	Does the Woreda maintain a record of health facility data quality assessments conducted in the past three months? (Please observe)	1. Yes, observed ☐	2. No ☐	
DQ_033	Does the Woreda maintain records of feedback to health facilities on data quality assessment findings? (Please observe)	1. Yes, observed ☐	2. No ☐	

DATA PROCESSING AND ANALYSIS				
DQ_034	Does the Woreda use an electronic database/system for routine health data management?	1. Yes ☐	2. No ☐	→DQ_036 (if No)
DQ_035	If yes, indicate the type of electronic system used for routine data management			

Electronic system	A. For data entry		B. For data analysis	
	1. Yes	2. No	1. Yes	2. No
01. National open-source data processing system (e.g., DHIS 2)				
02. National proprietary software (e.g., e-HMIS)				
03. Excel-based spreadsheet				
04. Access-based data processing module				
05. Other (specify)				

DQ_036	**ASK RELEVANT STAFF IN THE WOREDA OFFICE TO SHOW UP-TO-DATE (I.E., NOT MORE THAN ONE YEAR OLD) PAPER or ELECTRONIC REPORTS, DOCUMENTS, AND/OR DISPLAYS THAT CONTAIN THE FOLLOWING INFORMATION. THE ENUMERATOR SHOULD RECORD THE OBSERVATIONS ACCORDINGLY.**		
A	Aggregated/summary HMIS report	1. Yes, observed ☐	2. No ☐

Supplement to: Worku AG, Midekssa WD, Tilahun HA, et al. The impact of health information system interventions on maternal and child health service utilizations in Ethiopia: quasi-experimental study. *Glob Health Sci Pract*. 2024;12(6):2400145. https://doi.org/10.9745/GHSP-D-24-00145

B	Demographic data on the catchment population of the Woreda for calculating coverage.	1. Yes, observed ☐	2. No ☐
C	Indicators (e.g., Penta3 coverage) calculated for the woreda catchment within the review three months.	1. Yes, observed ☐	2. No ☐
D	Comparisons among facilities in the Woreda (e.g., for Penta3 coverage).	1. Yes, observed ☐	2. No ☐
E	Comparisons with Woreda/national targets.	1. Yes, observed ☐	2. No ☐
F	Comparisons of data over time (monitoring trends) (e.g., for ANC, Penta3, etc.).	1. Yes, observed ☐	2. No ☐
G	Comparisons of sex-disaggregated data (e.g., for Penta3, HIV testing, provider-initiated counseling and testing [PICT]).	1. Yes, observed ☐	2. No ☐
H	Comparisons of service coverage (e.g. ANC 4+, PNC within 48 hours, neonatal sepsis treatment).	1. Yes, observed ☐	2. No ☐

Supplement to: Worku AG, Midekssa WD, Tilahun HA, et al. The impact of health information system interventions on maternal and child health service utilizations in Ethiopia: quasi-experimental study. *Glob Health Sci Pract*. 2024;12(6):2400145. https://doi.org/10.9745/GHSP-D-24-00145

Part 2. Use of Information: Woreda Assessment Form

DATA VISUALIZATION					
DU_003	Does the Woreda office prepare minimum data visuals (graphs, tables, maps, etc.) showing achievements toward targets (indicators, geographic and/or temporal trends, and situation data)?	Yes, all, paper or electronic copies of data visuals observed at the Woreda offices ....1<br>Yes, some, paper or electronic copies of data visuals observed at the Woreda offices--------------2<br>No 0			➔DU_005 (if No)
DU_004	If yes, what type of information is captured in the data visuals and is it updated as per the standard? (Refer the annex for minimum wall charts)				
A	Map of catchment area	1. Yes, updated ☐	2. Yes, but not updated ☐	0. No ☐	
B	Catchment population profile	1. Yes, updated ☐	2. Yes, but not updated ☐	0. No ☐	
C	Staffing	1. Yes, updated ☐	2. Yes, but not updated ☐	0. No ☐	
D	Ten Top Causes of Morbidity (Males & Females)	1. Yes, updated ☐	2. Yes, but not updated ☐	0. No ☐	
E	Ten Top Causes of Morbidity In < 5 Children	1. Yes, updated ☐	2. Yes, but not updated ☐	0. No ☐	
E2	Ten Top Causes of Mortality in Hospitals	1. Yes, updated ☐	2. Yes, but not updated ☐	0. No ☐	
F	ANC coverage	1. Yes, updated ☐	2. Yes, but not updated ☐	0. No ☐	
G	Skilled attendant deliveries	1. Yes, updated ☐	2. Yes, but not updated ☐	0. No ☐	
H	Penta-3 immunization coverage	1. Yes, updated ☐	2. Yes, but not updated ☐	0. No ☐	
I	Measles immunization coverage	1. Yes, updated ☐	2. Yes, but not updated ☐	0. No ☐	
J	Malaria, all ages	1. Yes, updated ☐	2. Yes, but not updated ☐	0. No ☐	
K	Pneumonia amongst Under 1s	1. Yes, updated ☐	2. Yes, but not updated ☐	0. No ☐	
L	HIV/AIDS (VCT)	1. Yes, updated ☐	2. Yes, but not updated ☐	0. No ☐	
M	HIV/AIDS (PMTCT)	1. Yes, updated ☐	2. Yes, but not updated ☐	0. No ☐	
N	HIV/AIDS (ART)	1. Yes, updated ☐	2. Yes, but not updated ☐	0. No ☐	
N	HIV/AIDS (ART)	1. Yes, updated ☐	2. Yes, but not updated ☐	0. No ☐	
O	OPD attendance	1. Yes, updated ☐	2. Yes, but not updated ☐	0. No ☐	
P	Inpatient admission	1. Yes, updated ☐	2. Yes, but not updated ☐	0. No ☐	
Q	Average length of stay	1. Yes, updated ☐	2. Yes, but not updated ☐	0. No ☐	
R	Bed occupancy	1. Yes, updated ☐	2. Yes, but not updated ☐	0. No ☐	
DU_004_1	If Yes(1 &/or 2) to DU_004, Are the following data visuals displayed in places that are visible to managers, supervisors, mentors, health workers and the public?				
A	Map of catchment area	1.Yes, displayed	0.No		
B	Catchment population profile	1.Yes, displayed	0.No		
C	Staffing	1.Yes, displayed	0.No		

Supplement to: Worku AG, Midekssa WD, Tilahun HA, et al. The impact of health information system interventions on maternal and child health service utilizations in Ethiopia: quasi-experimental study. *Glob Health Sci Pract*. 2024;12(6):2400145. https://doi.org/10.9745/GHSP-D-24-00145

D	Ten Top Causes of Morbidity (Males & Females)	1.Yes, displayed	0.No		
E	Ten Top Causes of Morbidity In < 5 Children	1.Yes, displayed	0.No		
E2	Ten Top Causes of Mortality in Hospitals	1.Yes, displayed	0.No		
F	ANC coverage	1.Yes, displayed	0.No		
G	Skilled attendant deliveries	1.Yes, displayed	0.No		
H	Penta-3 immunization coverage	1.Yes, displayed	0.No		
I	Measles immunization coverage	1.Yes, displayed	0.No		
J	Malaria, all ages	1.Yes, displayed	0.No		
K	Pneumonia amongst Under 1s	1.Yes, displayed	0.No		
L	HIV/AIDS (VCT)	1.Yes, displayed	0.No		
M	HIV/AIDS (PMTCT)	1.Yes, displayed	0.No		
N	HIV/AIDS (ART)	1.Yes, displayed	0.No		
O	OPD attendance	1.Yes, displayed	0.No		
P	Inpatient admission	1.Yes, displayed	0.No		
Q	Average length of stay	1.Yes, displayed	0.No		
R	Bed occupancy	1.Yes, displayed	0.No		

HMIS ANALYTIC REPORT PRODUCTION			
DU_005	Does the Woreda have analyzed HMIS data (e.g., summary tables, charts, maps)?	Yes, observed paper-based____1<br>Yes, observed electronic_______2<br>No______________________________0	
DU_006	Does the Woreda office produce any report or bulletin (annual, quarterly, etc.) based on an analysis of HMIS data?<br>**(Excluding the monthly summary/aggregate reports submitted to the higher level.)**	Yes, observed.................... 1<br>No ______________________________0	➔DU_009 (if No)
DU_007	If yes, list the reports, indicating the frequency of the reports and number of times the reports were actually issued in the last 12 months.		

	Title of the report **(A)**	Number of times this report is supposed to be issued per year **(B)**	Number of times this report was actually issued in the last 12 months **(C)**	Target audience of the report (e.g., FMOH, RHB, ZHD, civil administration, parliament, community forums, general population) **(D)**
01				
02				
03				

Supplement to: Worku AG, Mideksa WD, Tilahun HA, et al. The impact of health information system interventions on maternal and child health service utilizations in Ethiopia: quasi-experimental study. *Glob Health Sci Pract*. 2024;12(6):2400145. https://doi.org/10.9745/GHSP-D-24-00145

DU_008	Do any of these reports and/or bulletins contain discussions and decisions/recommendations based on key performance targets based on HMIS data, such as:			
	01. Maternal and Child health	1. Yes ☐	0. No ☐	
	02. Health Promotion and Disease prevention	1. Yes ☐	0. No ☐	
	03. Public health emergency	1. Yes ☐	0. No ☐	
	04. Human resource management	1. Yes ☐	0. No ☐	
	05. Pharmaceuticals	1. Yes ☐	0. No ☐	
	06. Finance and Resource	1. Yes ☐	0. No ☐	
	07. Health Information System	1. Yes ☐	0. No ☐	

FEEDBACK TO HEALTH FACILITIES				
DU_009	Did the Woreda send feedback reports using HMIS information to health facilities in the review three months?<br>**(OBSERVE THE REPORT AND TICK ACCORDINGLY.)**	1. Yes, observed ☐	0. No ☐	→DU_011 (if No)
DU_010	If yes, indicate the types of feedback reports:			
A	Feedback on data quality (including data accuracy, reporting timeliness, and/or report completeness).	1. Yes, observed ☐	0. No ☐	
B	Feedback on service performance based on reported HMIS data, e.g., appreciation/acknowledgement of good performance; resource allocation/mobilization.	1. Yes, observed ☐	0. No ☐	

ROUTINE DECISION-MAKING FORUMS AND PROCESSES AT WOREDA OFFICE				
DU_011	Does the Woreda have performance monitoring team (PMT)?	1. Yes ☐	0. No ☐	→DU_023 (if No)
DU_012	Is the PMT membership according to the standard? (Head of the institution, HMIS in charge and representatives from each service or program unit/department)	1. Yes ☐	0. No ☐	
DU_013	How often are the performance monitoring team meetings supposed to take place?	Weekly .... 1<br>Monthly .... 2<br>Quarterly .... 3<br>Biannually .... 4<br>Annually .... 5<br>No schedule 0		
DU_014	How many times did the performance monitoring team meetings take place during the review three months?	More than four times .... 1<br>Four times .... 2<br>Three times .... 3<br>Two times .... 4<br>One time .... 5<br>None 0		→DU_023 (if None)

Supplement to: Worku AG, Midekssa WD, Tilahun HA, et al. The impact of health information system interventions on maternal and child health service utilizations in Ethiopia: quasi-experimental study. *Glob Health Sci Pract*. 2024;12(6):2400145. https://doi.org/10.9745/GHSP-D-24-00145

DU_015	Were minutes of performance monitoring team meetings maintained for the review three months?	Month 1		Month 2		Month 3		→DU_023 (if No to all)
		1. Yes ☐	0. No ☐	1. Yes ☐	0. No ☐	1. Yes ☐	0. No ☐	
DU_016	Is the PMT using a standard PMT minute record/logbook?<br><br>(Standard: Data Quality and Performance Monitoring logbook)	1. Yes, observed ☐				0. No ☐		→DU_018 (if Yes)
DU_017	Please indicate the reason(s) for not using the standard minute record/logbook.<br><br>(select all that apply)	Standard logbook is not available.........1<br>Staff not trained/familiar with the logbook.........2<br>Logbook is not user friendly.........3<br>Other (Specify): ____________96						
	Please check the performance monitoring team minute/records for the previous three months to see who chaired the meetings each month.							
DU018_01	Who chaired the meetings in Month 1?	Head/deputy head of woreda health office.........1<br>HMIS in charge.........2<br>Process owner/department representatives........3						→DU_020 (if "1" selected)
DU018_02	Who chaired the meetings in Month 2?	Head/deputy head of woreda health office.........1<br>HMIS in charge.........2<br>Process owner/department representatives........3						→DU_020 (if "1" selected)
DU018_03	Who chaired the meetings in Month 3?	Head/deputy head of woreda health office.........1<br>HMIS in charge.........2<br>Process owner/department representatives........3						→DU_020 (if "1" selected)
DU_019	Please indicate the reason(s) if the head/deputy head of the woreda health office did not chair the meeting (ask the HMIS focal person)	Not available during the meeting.........1<br>Other competing priorities.........2<br>Lack of familiarity with the subject.........3<br>Other (specify).........4						
DU_020	Please check the performance monitoring team minute/records for the review months and see if the following topics were discussed.							
A	Were there discussions on HMIS management, such as data quality, completeness, or timeliness of reporting?	1. Yes ☐			0. No ☐			→DU_021 (if No)
B	Were HMIS related issues identified and prioritized?	1. Yes ☐			0. No ☐			→DU_021 (if No)
C	Were root cause analyses conducted for the prioritized HMIS related issues?	1. Yes ☐			0. No ☐			
D	Was an action plan developed to address the HMIS related issues?	1. Yes ☐			0. No ☐			
E	Have any follow-up actions taken place based on decisions made during previous meetings on HMIS-related issues? (e.g., referring HMIS-related issues to the higher level)	1. Yes ☐			0. No ☐			

Supplement to: Worku AG, Midekssa WD, Tilahun HA, et al. The impact of health information system interventions on maternal and child health service utilizations in Ethiopia: quasi-experimental study. *Glob Health Sci Pract*. 2024;12(6):2400145. https://doi.org/10.9745/GHSP-D-24-00145

DU_021	Were discussions held to review the following key performance targets (e.g., tracking progress against targets/plan):<br><br>((Please check the performance monitoring team minute/records for the review months and see if the following topics were discussed)			→DU_022 (if all are No)
	01. Maternal and Child health	1. Yes ☐	0. No ☐	
	02. Health Promotion and Disease prevention	1. Yes ☐	0. No ☐	
	03. Public health emergency	1. Yes ☐	0. No ☐	
	04. Human resource management	1. Yes ☐	0. No ☐	
	05. Pharmaceuticals	1. Yes ☐	0. No ☐	
	06. Finance and Resource	1. Yes ☐	0. No ☐	
	07. Health Information System	1. Yes ☐	0. No ☐	
A	Has the PMT identified and prioritized performance issues?	1. Yes ☐	0. No ☐	→DU_021F (if No)
B	Did the PMT conduct root cause analysis for the prioritized performance related issues?	1. Yes ☐	0. No ☐	
C	Has the PMT made any of the following decisions based on discussions of the Woreda and/or health facility's performance?			
	01. Formulation of plans	1. Yes ☐	0. No ☐	
	02. Budget preparation	1. Yes ☐	0. No ☐	
	03. Budget reallocation	1. Yes ☐	0. No ☐	
	04. Medicine supply and drug management	1. Yes ☐	0. No ☐	
	05. Human resource management (training, reallocation, etc.)	1. Yes ☐	0. No ☐	
	06. Advocacy for policy, programmatic, or strategic decisions from the higher level	1. Yes ☐	0. No ☐	
	07. Health services (preventive, promotive, clinical, rehabilitative) planning	1. Yes ☐	0. No ☐	
	08. Promotion of service quality/improvement	1. Yes ☐	0. No ☐	
	09. Reducing the gender gap in the provision of health services	1. Yes ☐	0. No ☐	
	10. Involvement of the community and local government	1. Yes ☐	0. No ☐	
D	Was an action plan developed?	1. Yes ☐	0. No ☐	
E	Has any follow-up action taken place based on decisions made during previous meetings on performance-related issues? (e.g., referring performance-related issues for solution to the higher	1. Yes ☐	0. No ☐	
F	Are any data display tools used during the performance review meetings?	1. Yes ☐	0. No ☐	

Supplement to: Worku AG, Mideksa WD, Tilahun HA, et al. The impact of health information system interventions on maternal and child health service utilizations in Ethiopia: quasi-experimental study. *Glob Health Sci Pract*. 2024;12(6):2400145. https://doi.org/10.9745/GHSP-D-24-00145

G	Which display tools were used during the performance review meetings in the review three months?	Manual/paper-based 1<br>Electronic 2<br>Both 3		
H	Was there any attempt to use other sources of data (other than the HMIS) for triangulation of evidence at the performance review meeting in the review three months?	1. Yes ☐	0. No ☐	→DU_022 (if No)
I	Which sources were used?<br>(Multiple answer is possible)	Census 1<br>Population-based surveys 2<br>Health facility surveys 3<br>Civil registration and vital events 4<br>Operations research 5<br>Other (Specify):__ ____96		
DU_022	Is the PMT tracking key quality and equity indicators from the transformation plan during PMT review?<br><br>(Please check the performance monitoring team minute/records or other documents for the review months and see if the quality and/or equity indicators are addressed)	Yes, both quality and equity indicators are tracked…………...1<br>Either quality or equity indicators are tracked (not both)…......…...2<br>Neither quality or equity indicators are tracked…………..0		
DU_022_1	Were the performance review meeting minutes circulated to/ signed by all members of the PMT?	1. Yes, observed ☐	2. No ☐	

STRATEGIC AND ANNUAL PLANNING				
DU_023	Does the Woreda have a five years strategic plan of the woreda?	1. Yes, observed ☐	0. No ☐	
DU_024	Does the Woreda have current woreda-based annual plan?	1. Yes, observed ☐	0. No ☐	→DU_028 (if No)
DU_025	Does the woreda-based plan reflect use of data from the HMIS for problem identification and/or root cause analysis?	1. Yes ☐	0. No ☐	
DU_026	Does the current woreda-based plan reflect use of HMIS data for target setting?	1. Yes ☐	0. No ☐	

SUPERVISION BY THE ZONE/REGION				
DU_028	How many times did the Zone/region supervisor visit your Woreda health office over the review three months?	More than four times 1<br>Four times 2<br>Three times 3<br>Two times 4<br>One time 5<br>None 0		→DU_034a (if None)
DU_029	Did the supervisor use the integrated supportive supervision or HMIS checklist during the most recent visit?	1. Yes ☐	0. No ☐	
DU_030	Did the supervisor (s) check the data quality?	1. Yes ☐	0. No ☐	

Supplement to: Worku AG, Midekssa WD, Tilahun HA, et al. The impact of health information system interventions on maternal and child health service utilizations in Ethiopia: quasi-experimental study. *Glob Health Sci Pract*. 2024;12(6):2400145. https://doi.org/10.9745/GHSP-D-24-00145

DU_031	During the most recent visit, did the Zone/Region supervisor discuss your Woreda's performance based on HMIS information?	1. Yes ☐	0. No ☐	→DU_033 (if No)
DU_032	If DU_031 is Yes, did the supervisor help you to make a decision or take corrective action based on the discussion?	1. Yes ☐	0. No ☐	
DU_033	Did the supervisor send a report/ written feedback to the woreda health office on the last supervisory visit(s)?	1. Yes ☐	0. No ☐	
**SUPERVISION BY THE WOREDA HEALTH OFFICE**				
DU_034a	Did the Woreda Health Office conduct internal mentorship in review three months?	1. Yes ☐	0. No ☐	→DU_35 (if No)
DU_034b	If yes, please check if there is written feedback on the last internal mentorship?	1. Yes, observed ☐	0. No ☐	
DU_035	Did the woreda health office conduct mentorship to the lower levels in the review three months?	1. Yes, to all facilities<br>2. Yes, to some facilities<br>0. No		

**DATA DISSEMINATION OUTSIDE HEALTH SECTOR**				
DU_036	Did the Woreda submit/present last year's health sector performance reports and action items to a Woreda council in the past 12 months?	1. Yes ☐	0. No ☐	→DU_038 (if No)
DU_037	Do those reports/presentations use data from the HMIS to show the health sector's progress?	1. Yes, observed ☐	0. No ☐	
DU_038	Is there a website updated at least annually for accessing the Woreda's HMIS data by the general public?	1. Yes ☐	0. No ☐	
DU_039	Are woreda performance data shared with general public via printed material (e.g., brochure, newsletter, etc.) in the last six months?	1. Yes ☐	0. No ☐	
DU_040	Did the Woreda Health Office conduct assessment(s) and disseminate findings in the last six months?	1. Yes, assessment is conducted and finding(s)disseminated ☐<br>2. Assessment is conducted, but findings not disseminated ☐<br>0. No assessment conducted ☐		
DU_041	Did the Woreda Health Office hold performance review meeting with community representatives in the last six months?	1. Yes ☐	0. No ☐	

Supplement to: Worku AG, Midekssa WD, Tilahun HA, et al. The impact of health information system interventions on maternal and child health service utilizations in Ethiopia: quasi-experimental study. *Glob Health Sci Pract*. 2024;12(6):2400145. https://doi.org/10.9745/GHSP-D-24-00145

GPS COORDINATES				
**DQ_110**	Please capture the GPS coordinates of health office. **(Note: Enumerator may have to go outside to capture the coordinates)**			

Supplement to: Worku AG, Midekssa WD, Tilahun HA, et al. The impact of health information system interventions on maternal and child health service utilizations in Ethiopia: quasi-experimental study. *Glob Health Sci Pract*. 2024;12(6):2400145. https://doi.org/10.9745/GHSP-D-24-00145

Data use and data quality assessment tool: Health Facility Level

Purpose

4. Identify RHIS data quality, disaggregated data, and information use issues.
5. Quantify the levels of data quality (accuracy, reporting timeliness, and completeness) and information use status (access to RHIS data, existence of analyzed data, and use of RHIS data for monitoring and planning).
6. Identify issues/problems with data processing and processes for information use.

Summary of Information Collected via the RHIS Performance Diagnostic Tool at the Health Facility Level

Measuring Data Quality

Through analysis of program indicators, the RHIS Performance Diagnostic Tool quantifies the status of data completeness, timeliness, and accuracy, and thus provides valuable information on the adequacy of health facility data to support planning and monitoring. The data quality assessment section of this tool is aligned with the data verification aspect of the DQR Tool. The RHIS Performance Diagnostic Tool has the following core recommended indicators to assess data quality:

S.N	Category	Indicators (data elements)	Government HC and above	HP	Private HF	Remark
1	Maternal health	Number of births attended by skilled attendants	√		√	
2	Family planning	Total number of family planning (new and repeat) acceptors by age	√	√	√	
3	Immunization	Number of children under one year of age who have received third dose of pentavalent (Penta3) vaccine	√	√	√	
4	HIV service	Number of clients tested HIV positive	√		√	
5	Disease	Number of under-five children with pneumonia	√	√	√	
		Confirmed malaria cases (microscopy or RDT)	√	√	√	
7	Service quality	Number of inpatient deaths	√		√*	
8	Tuberculosis	TB cases (all types)	√		√	

***Private Hospitals**

Supplement to: Worku AG, Midekssa WD, Tilahun HA, et al. The impact of health information system interventions on maternal and child health service utilizations in Ethiopia: quasi-experimental study. *Glob Health Sci Pract*. 2024;12(6):2400145. https://doi.org/10.9745/GHSP-D-24-00145

At the facility level, the diagnostic tool compares the reported value of an indicator for a selected reporting period to recoded data by reviewing the source document for the same facility and period. The result is an estimate of the accuracy of reporting for the indicators in question for the whole program.

Measuring Information Use

The tool measures the continuous use of information to guide day-to-day operations, track performance, learn from past results, and improve service delivery. The tool focuses on the use of RHIS data for analytic report production, discussion, decision/actions, target setting, planning, and monitoring.

Assessing RHIS Data Management Processes

This section assesses various aspects of RHIS data management processes, including:

- Data processing, analysis, and presentation: the availability of a copy of HMIS data management guidelines; use of standardized RHIS data collection and reporting tools; evidence of data analysis; and visual representation of data.

- Data quality check: presence of data quality assurance guideline and tools; clearly assigned roles and responsibilities for data review; and regular internal data quality checks conducted by the health facility.

- Feedback: existence of formal feedback loops to the staff collecting the data; health facilities receive regular written feedback on their performance; and quality of reported data.

- Supervision quality: supervision frequency; checking data quality; using data for discussion; helping in decision making; and supervisory feedback.

Data Collection Method

- Key informant interviews (health facility in-charge and data manager, or those responsible for compilation, reporting, and analysis of data)

- Document review and observation (HMIS reports, electronic database, planning documents, meeting minutes, feedback reports/notes, guidelines)

Supplement to: Worku AG, Mideksa WD, Tilahun HA, et al. The impact of health information system interventions on maternal and child health service utilizations in Ethiopia: quasi-experimental study. *Glob Health Sci Pract*. 2024;12(6):2400145. https://doi.org/10.9745/GHSP-D-24-00145

Part I. Data Quality Assessment: Health Facility Form

INTERVIEWER VISITS		
FQ_101.	Interview date	
FQ_102.	Interviewer name	
FQ_103.	Interviewer code<br>Please enter your 3-character identifier.	
**FACILITY IDENTIFICATION**		
FQ_104.	Please select the region	Addis Ababa<br>Afar<br>Amhara<br>Beneshangul Gumuz<br>Dire Dawa<br>Gambella<br>Harari<br>Oromiya<br>SNNPR<br>Somali<br>Tigray
FQ_105.	Zone/Sub-city<br>Please enter the alphanumeric code that identifies this level (up to 11 alphanumeric digits).	
FQ_106.	Woreda<br>Please enter the alphanumeric code that identifies this level (up to 11 alphanumeric digits).	
FQ_107.	Health facility number<br>(Please enter the unit number up to 10 digits, include leading zeros.)	
FQ_108.	Health facility name	
FQ_109.	Type of health facility<br>**(If "6" selected exit survey and go to Health Post Diagnostic Tool)**	Referral/Specialized hospital .... 1<br>General hospital .... 2<br>Primary hospital .... 3<br>Health center .... 4<br>Medium clinic .... 5<br>Health post 6
FQ_110.	Urban/rural	Urban .... 1<br>Rural 2
FQ_111.	Managing authority	Government/public 1

		NGO/not-for-profit ....2<br>Private-for-profit ....3<br>Mission/faith-based ....4<br>Other (specify)<br>________________96

GENERAL INFORMATION

READ THE FOLLOWING STATEMENT TO THE MANAGER, THE PERSON IN-CHARGE OF THE FACILITY, OR THE MOST SENIOR HEALTH WORKER RESPONSIBLE FOR OUTPATIENT SERVICES WHO IS PRESENT AT THE FACILITY.

Good day! My name is ____________________. We are here on behalf of [IMPLEMENTING AGENCY] conducting a survey of health facilities to assist the government in knowing more about the performance of routine health information systems in [COUNTRY].

Your facility was randomly selected to participate in this study. We will be asking you questions about various health services and reporting of those services. Information about your facility may be used by the MOH, organizations supporting services in your facility, and researchers for planning service improvements or for conducting further studies of health services.

Neither your name nor that of any other health worker respondents participating in this study will be included in the dataset or in any report; however, there is a small chance that any of these respondents may be identified later. We are asking for your help to ensure that the information we collect is accurate.

You may refuse to answer any question or choose to stop the interview at any time. However, we hope you will answer the questions, which will benefit the clients you serve, the nation, and yourself.

If there are questions for which someone else is the most appropriate person to provide the information, we would appreciate it if you would introduce us to that person to help us collect the information.

At this point, do you have any questions about the study? Do I have your agreement to proceed?

____

INTERVIEWER'S SIGNATURE INDICATING CONSENT OBTAINED

FQ_112	May I begin the interview?	1. YES ☐<br>2. NO ☐	

PLEASE SELECT THE 3 REVIEW MONTHS THAT WILL BE USED DURING THIS ASSESSMENT

Month_1	Meskerem 2012............1<br>Tikemet 2012............2<br>Hidar 2012............3<br>Tahesas 2012............4<br>Tir 2012............5<br>Yekatit 2012............6<br>Megabit 2012............7<br>Miyazia 2012............8<br>Ginbot 2012............9<br>Sene 2012............10<br>Hamle 2012............11<br>Nehase 2012............12
Month_2	

Supplement to: Worku AG, Midekssa WD, Tilahun HA, et al. The impact of health information system interventions on maternal and child health service utilizations in Ethiopia: quasi-experimental study. *Glob Health Sci Pract*. 2024;12(6):2400145. https://doi.org/10.9745/GHSP-D-24-00145

	Meskerem 2012……………………………………1<br>Tikemet 2012……………………………………2<br>Hidar 2012………………………………………3<br>Tahesas 2012……………………………………4<br>Tir 2012…………………………………………5<br>Yekatit 2012……………………………………6<br>Megabit 2012……………………………………7<br>Miyazia 2012……………………………………8<br>Ginbot 2012……………………………………9<br>Sene 2012………………………………………10<br>Hamle 2012……………………………………11<br>Nehase 2012……………………………………12
Month_3	Meskerem 2012……………………………………1<br>Tikemet 2012……………………………………2<br>Hidar 2012………………………………………3<br>Tahesas 2012……………………………………4<br>Tir 2012…………………………………………5<br>Yekatit 2012……………………………………6<br>Megabit 2012……………………………………7<br>Miyazia 2012……………………………………8<br>Ginbot 2012……………………………………9<br>Sene 2012………………………………………10<br>Hamle 2012……………………………………11<br>Nehase 2012……………………………………12

RESOURCES FOR DATA ASSESSMENT			
FQ_010	Is there a designated person(s) to enter data/compile reports from the different units in the health facility?	1. Yes ☐	0. No ☐
FQ_011	Does the PMT review the quality of compiled data prior to submission to the next level, e.g., to Woreda health office, ZHD, RHB, FMOH, etc?	Yes -- 1<br>No--0	
FQ_012	If FQ_010 OR FQ_011 is 1, Are designated staff/PMT members trained in:		
	A. HMIS related data entry/compilation	Yes (all staff have received training in the past 12 months)--1<br>Mostly (all staff have received training but not in the past 12 months)----------------------------2<br>Partly (some staff have received training in the past 12 months----3<br>Not at all--0	
	B. HMIS related data review and quality control?	Yes (all staff have received training in the past 12 months) --1<br>Mostly (all staff have received training but not in the past 12 months)------------------------------2<br>Partly (some staff have received training in the past 12 months)----3	

Supplement to: Worku AG, Midekssa WD, Tilahun HA, et al. The impact of health information system interventions on maternal and child health service utilizations in Ethiopia: quasi-experimental study. *Glob Health Sci Pract*. 2024;12(6):2400145. https://doi.org/10.9745/GHSP-D-24-00145

		Not at all 0

**1. MATERNAL HEALTH INDICATOR**				
**SKILLED BIRTH ATTENDANTS (SBA)**				
**FQ_013**	Does this facility provide delivery services?	1. Yes ☐	0. No ☐	➔FQ_024 (if No)
**SOURCE DOCUMENTS AND REPORTS**				
**FQ_014**	Does this facility report facility delivery/SBA data to a reporting system?	1. Yes ☐	0. No ☐	➔FQ_024
**FQ_015**	To which of the following reporting systems does the facility report SBA data?<br>(please check the reporting form used by the health facility)			
**A**	Health Management Information System (HMIS)	1. Yes ☐	0. No ☐	
**B**	Program specific reporting system for maternal and child health (MCH)	1. Yes ☐	0. No ☐	
**C**	Nongovernmental organizations or institutions	1. Yes ☐	0. No ☐	
**D**	Other reporting system	1. Yes ☐<br>________<br>SPECIFY	0. No ☐	
**FQ_016**	What is the source document used by this facility for monthly reporting of **SBA** services? We are primarily interested in the document that is used for **compiling** the total number of SBA at this facility. Please report if any customized documents are used.	Delivery register .... 1<br>Tally sheets .... 2<br>Patient cards .... 3<br>Other (specify) ____________ 96		
**REVIEW THE SOURCE DOCUMENT USED TO COMPILE AND SUMMARIZE INFORMATION FOR MONTHLY REPORTING (I.E., REGISTER, TALLY SHEET) FOR SBA AND ANSWER THE FOLLOWING QUESTIONS.**				

**FQ_017**	Please confirm the availability of the **source document** for SBA for month 1 to month 3. If available, please **recount** the number of SBA recorded in the **delivery register** for month 1 to month 3.	**(A) Source document available**				**(B) Recount the number of SBA in the delivery register (if none, please enter 0)**
		**Yes, available and complete***	**Yes, available but partly** complete**	**Yes, available but no data recorded**	**No**	
**01**	**Month 1**	1	2	3	0	☐☐☐☐

Supplement to: Worku AG, Midekssa WD, Tilahun HA, et al. The impact of health information system interventions on maternal and child health service utilizations in Ethiopia: quasi-experimental study. *Glob Health Sci Pract*. 2024;12(6):2400145. https://doi.org/10.9745/GHSP-D-24-00145

**02**	**Month 2**	1	2	3	0	☐☐☐☐
**03**	**Month 3**	1	2	3	0	☐☐☐☐

Take the last 15 entries recorded in the source document for each reporting period and check if all the data elements relevant to the selected indicator are filled in.

***COMPLETE means that the source document contains the data elements relevant to the selected indicator.**PARTLY: the source document is available but some information is missing.**

FQ_018	If the source document (delivery register) is not available, what are the possible reasons? (Don't read the choices)	Storage or archiving problems ....1<br>Absence of designated staff....2<br>Stock out of source document ....3<br>Other (specify): ________96
FQ_019	If the source document (delivery register) is not completely filled in, what are the possible reasons for the missing data? (Don't read the choices)	Staffing issue(s)(shortage, absence)....1<br>Not understanding the data element ....2<br>Presence of other vertical reporting requirement ....3<br>Data burden (too much data elements to be recorded) 4<br>The recording tool is not designed as user friendly ....5<br>Other (specify): ________96

REVIEW MONTHLY REPORTS FOR SKILLED BIRTH ATTENDANTS (SBA) AND ANSWER THE FOLLOWING QUESTIONS.

**FQ_020**	Please confirm the availability of the **monthly report** for SBA for month 1 to month 3. If available, please **record** the number of SBA recorded in the **monthly report** for month 1 to month 3.	**(A) Monthly report available**				**(B) Record the number of SBA from the monthly report (if missing, leave blank)**
		**Yes, available and complete***	**Yes, available but partly** complete**	**Yes, available but no data recorded**	**No**	
**01**	**Month 1**	1		3	0	☐☐☐☐
**02**	**Month 2**	1		3	0	☐☐☐☐
**03**	**Month 3**	1		3	0	☐☐☐☐

***COMPLETE means that the monthly report contains the data relevant to the selected indicator.**
****PARTLY: the monthly report is available but some information is missing.**

DATA INCOMPLETENESS

FQ_021	If the monthly report of SBA is not available, what are the possible reasons?	Storage or archiving problems ....1<br>Absence of designated staff....2<br>Stock out of source document ....3<br>Other (specify): ________96
FQ_022	If the monthly report of SBA is partially complete or has no data, what are the possible reasons for the missing data?	Staffing issue(s)(shortage, absence) ....1<br>Not understanding the data element 2

Supplement to: Worku AG, Midekssa WD, Tilahun HA, et al. The impact of health information system interventions on maternal and child health service utilizations in Ethiopia: quasi-experimental study. *Glob Health Sci Pract*. 2024;12(6):2400145. https://doi.org/10.9745/GHSP-D-24-00145

		Presence of other vertical reporting requirement ....3<br>Data burden (too much data elements to be recorded) ....4<br>The recording tool is not designed as user friendly ....5<br>There is no client to be reported ....6<br>Other (specify): ________________________96
**DISCREPANCIES**		
FQ_023	If there was a discrepancy observed between the **source document (delivery register)** and the **monthly report**, what are the reasons for the discrepancy?<br><br>(circle all that apply)	Data entry errors..1<br>Arithmetic errors..2<br>Information from all source documents not compiled correctly ....3<br>Data burden (too much data elements to be reported) ....4<br>Illegible writing on the source document (not readable) ....5<br>Lack of emphasis for data accuracy.....................6<br>Other (specify) ____________________96

**2. IMMUNIZATION INDICATOR**				
**PENTAVALENT THIRD DOSE (PENTA3) IN CHILDREN UNDER 1 YEAR**				
**FQ_024**	Does this facility provide immunization services?	1. Yes ☐	0. No ☐	➔FQ_035 (if No)
**SOURCE DOCUMENTS AND REPORTS**				
**FQ_025**	Does this facility report immunization data to a reporting system?	1. Yes ☐	0. No ☐	➔FQ_035 (if No)
**FQ_026**	To which of the following reporting systems does the facility report immunization data? (Please check the reporting form used by the health facility)			
**A**	Health Management Information System (HMIS)	1. Yes ☐	0. No ☐	
**B**	Immunization/EPI program	1. Yes ☐	0. No ☐	
**C**	Nongovernmental organizations or institutions	1. Yes ☐	0. No ☐	
**D**	Other reporting system	1. Yes ☐<br>__________<br>SPECIFY	0. No ☐	
**FQ_027**	What is the source document used by this facility for monthly reporting of Penta3? We are primarily interested in the source document that is used for compiling monthly summary statistics for Penta3. Please report if any locally developed documents are used.	EPI register ....1<br>Immunization tally sheets ....2<br>Other (specify) ________________________96		

Supplement to: Worku AG, Midekssa WD, Tilahun HA, et al. The impact of health information system interventions on maternal and child health service utilizations in Ethiopia: quasi-experimental study. *Glob Health Sci Pract*. 2024;12(6):2400145. https://doi.org/10.9745/GHSP-D-24-00145

REVIEW THE SOURCE DOCUMENTS USED TO COMPILE AND SUMMARIZE INFORMATION FOR MONTHLY REPORTING FOR PENTA3 AND ANSWER THE FOLLOWING QUESTIONS						
**FQ_028**	Please confirm the availability of **source documents** for Penta3 for month 1 to month 3. If available, please **Recount** the number of DTP3 (Penta3) immunizations recorded in the **source document** for month 1 to month 3	**(A) Source documents available**				**(B) Recount the number of Penta3 immunizations in the source documents (if none, please enter 0)**
		**Yes, available and complete***	**Yes, available but partly** complete**	**Yes, available but no data recorded**	**No**	
**01**	**Month 1**	1	2	3	0	☐☐☐☐
**02**	**Month 2**	1	2	3	0	☐☐☐☐
**03**	**Month 3**	1	2	3	0	☐☐☐☐

Take the last 15 entries recorded in the register for each reporting period and check if all the data elements relevant to the selected indicator are filled in.

***COMPLETE means that the source document contains the data relevant to the selected indicator. **PARTLY: the register is available but some information is missing.**

FQ_029	If the source document (EPI register) is not available, what are the possible reasons?	Storage or archiving problems ....1<br>Absence of designated staff....................2<br>Stock out of source document ....3<br>Other (specify): ____________________96
**FQ_030**	If the source documents (EPI register) are partially complete or has no data, what are the possible reasons for the missing data?	Staffing issue(s)(shortage, absence)....................1<br>Not understanding the data element ....2<br>Presence of other vertical reporting requirement ....3<br>Data burden (too much data elements to be recorded)<br>4<br>The recording tool is not designed as user friendly ....5<br>Other (specify): ____________________96

REVIEW THE MONTHLY REPORTS FOR PENTA3 AND ANSWER THE FOLLOWING QUESTIONS.						
**FQ_031**	Please confirm the availability of **monthly reports** for month 1 to month 3. If available, please **report** the number of Penta3 immunizations recorded in the **Service monthly reports** for month 1 to month 3	**(A) Monthly reports available**				**(B) Record the number of DPT3 immunizations from the monthly reports (if missing, leave blank)**
		**Yes, available and complete***	**Yes, available but partly** complete**	**Yes, available but no data recorded**	**No**	

Supplement to: Worku AG, Midekssa WD, Tilahun HA, et al. The impact of health information system interventions on maternal and child health service utilizations in Ethiopia: quasi-experimental study. *Glob Health Sci Pract*. 2024;12(6):2400145. https://doi.org/10.9745/GHSP-D-24-00145

**01**	**Month 1**	1	[redacted]	3	0	
**02**	**Month 2**	1		3	0	
**03**	**Month 3**	1		3	0	

***COMPLETE means that the monthly report contains the data relevant to the selected indicator.**
****PARTLY: the monthly report is available but some information is missing.**

**DATA COMPLETENESS**		
FQ_032	If the monthly report of EPI is not available, what are the possible reasons?	Storage or archiving problems ....1<br>Absence of designated staff....................2<br>Stock out of source document ....3<br>Other (specify): ________________96
FQ_033	If the monthly report of EPI is partially complete or has no data, what are the possible reasons for the missing data?	Staffing issue(s)(shortage, absence) ....1<br>Not understanding the data element ....2<br>Presence of other vertical reporting requirement ....3<br>Data burden (too much data elements to be recorded) ....4<br>The recording tool is not designed as user friendly ....5<br>Other (specify): ________________96
**DISCREPANCIES**		
**FQ_034**	If there was a discrepancy observed between the **source document** and the **monthly report**, what are the reasons for the discrepancy?	Data entry errors....................1<br>Arithmetic errors....................2<br>Information from all source documents not compiled correctly ....3<br>Data burden (too much data elements to be reported) ....4<br>Illegible writing on the source document (not readable) ....5<br>Lack of emphasis for data accuracy....................6<br>Other (specify) ________________96

**3. FAMILY PLANNING INDICATOR**				
**TOTAL NUMBER OF WOMEN RECEIVED MODERN CONTRACEPTIVE METHODS**				
**FQ_035**	Does this facility provide Family planning services?	1. Yes ☐	0. No ☐	➔FQ_046 (if No)
**SOURCE DOCUMENTS AND REPORTS**				
**FQ_036**	Does this facility report family planning data to a reporting system?	1. Yes ☐	0. No ☐	➔FQ_046 (if No)
**FQ_037**	To which of the following reporting systems does the facility report family planning data? (please check the reporting form used by the health facility)			

Supplement to: Worku AG, Midekssa WD, Tilahun HA, et al. The impact of health information system interventions on maternal and child health service utilizations in Ethiopia: quasi-experimental study. *Glob Health Sci Pract*. 2024;12(6):2400145. https://doi.org/10.9745/GHSP-D-24-00145

**A**	Health Management Information System (HMIS)	1. Yes ☐	0. No ☐	
**B**	family planning department/program	1. Yes ☐	0. No ☐	
**C**	Nongovernmental organizations or institutions	1. Yes ☐	0. No ☐	
**D**	Other reporting system	1. Yes ☐ ________ SPECIFY	0. No ☐	

**FQ_038**	What is the source document used by this facility for monthly reporting of family planning? We are primarily interested in the document that is used for compiling monthly summary statistics for family planning. Please report if any locally developed documents are used.	Family planning register .... 1<br>Family planning tally sheets .... 2<br>Other (specify) ________ 96

REVIEW THE SOURCE DOCUMENTS USED TO COMPILE AND SUMMARIZE INFORMATION FOR MONTHLY REPORTING FOR FAMILY PLANNING AND ANSWER THE FOLLOWING QUESTIONS

**FQ_039**	Please confirm the availability of **source documents** for family planning users for month 1 to month 3. If available, please **Recount** the number of family planning users recorded in the **source document** for month 1 to month 3.	**(A) Source documents available**				**(B) Recount the number of family planning users in the source documents (if none, please enter 0)**
		**Yes, available and complete***	**Yes, available but partly** complete**	**Yes, available but no data recorded**	**No**	
**01**	**Month 1**	1	2	3	0	☐☐☐☐
**02**	**Month 2**	1	2	3	0	☐☐☐☐
**03**	**Month 3**	1	2	3	0	☐☐☐☐

Take the last 15 entries recorded in the FP register for each reporting period and check if all the data elements relevant to the selected indicator are filled in.

***COMPLETE means that the source document contains the data relevant to the selected indicator.**

****PARTLY: the register is available but some information is missing.**

FQ_040	If the source document (family planning register) is not available, what are the possible reasons?	Storage or archiving problems .... 1<br>Absence of designated staff .... 2<br>Stock out of source document .... 3<br>Other (specify): ________ 96

Supplement to: Worku AG, Midekssa WD, Tilahun HA, et al. The impact of health information system interventions on maternal and child health service utilizations in Ethiopia: quasi-experimental study. *Glob Health Sci Pract*. 2024;12(6):2400145. https://doi.org/10.9745/GHSP-D-24-00145

**FQ_041**	If the source documents are partially complete or has no data, what are the possible reasons for the missing data?	Staffing issue(s)(shortage, absence)........1<br>Not understanding the data element ....2<br>Presence of other vertical reporting requirement ....3<br>Data burden (too much data elements to be recorded) 4<br>The recording tool is not designed as user friendly ....5<br>Other (specify): ________96

REVIEW THE MONTHLY REPORTS FOR DPT3 (PENTA3) AND ANSWER THE FOLLOWING QUESTIONS.

**FQ_042**	Please confirm the availability of **monthly reports** for month 1 to month 3. If available, please **report** the number of family planning users recorded in the **monthly reports** for month 1 to month 3.	**(A) Monthly reports available**				**(B) Record the number of FP users from the monthly reports (if missing, leave blank)**
		**Yes, available and complete***	**Yes, available but partly** complete**	**Yes, available but no data recorded**	**No**	
**01**	**Month 2**	1	2	3	0	
**02**	**Month 3**	1	2	3	0	
**03**	**Month 2**	1	2	3	0	

***COMPLETE means that the monthly report contains the data relevant to the selected indicator.**
****PARTLY: the monthly report is available but some information is missing.**

DATA COMPLETENESS

FQ_043	If the monthly report of FP is not available, what are the possible reasons?	Storage or archiving problems ....1<br>Absence of designated staff........2<br>Stock out of source document ....3<br>Other (specify): ________96
FQ_044	If the monthly report of FP is partially complete or has no data, what are the possible reasons for the missing data?	Staffing issue(s)(shortage, absence) ....1<br>Not understanding the data element ....2<br>Presence of other vertical reporting requirement ....3<br>Data burden (too much data elements to be recorded) ....4<br>The recording tool is not designed as user friendly ....5<br>Other (specify): ________96

DISCREPANCIES

Supplement to: Worku AG, Midekssa WD, Tilahun HA, et al. The impact of health information system interventions on maternal and child health service utilizations in Ethiopia: quasi-experimental study. *Glob Health Sci Pract*. 2024;12(6):2400145. https://doi.org/10.9745/GHSP-D-24-00145

**FQ_045**	If there was a discrepancy observed between the **source document** and the **monthly report**, what are the reasons for the discrepancy?	Data entry errors____1<br>Arithmetic errors____2<br>Information from all source documents not compiled correctly ……3<br>Data burden (too much data elements to be reported) ……4<br>Illegible writing on the source document (not readable) ……5<br>Lack of emphasis for data accuracy……6<br>Other (specify) ________96

	**4. HIV INDICATOR**			
	**NUMBER OF CLIENTS WHO TESTED POSITIVE FOR HIV**			
**FQ_046**	Does this facility provide VCT/PIHTC/HIV testing services?	1. Yes ☐	0. No ☐	➔FQ_057 (if No)
**SOURCE DOCUMENTS AND REPORTS**				
**FQ_047**	Does this facility report VCT/PIHTC/HIV testing data to a reporting system?	1. Yes ☐	0. No ☐	➔FQ_057 (if No)
**FQ_048**	To which of the following reporting systems does the facility report VCT/PIHTC/HIV testing data?<br>(Please check the monthly reporting forms used by the health facility)			
**A**	Health Management Information System (HMIS)	1. Yes ☐	0. No ☐	
**B**	HIV process owner/program	1. Yes ☐	0. No ☐	
**C**	Nongovernmental organizations or institutions	1. Yes ☐	0. No ☐	
**D**	Other reporting system	1. Yes ☐<br>________<br>SPECIFY	0. No ☐	
**FQ_049**	What is the source document used by this facility for monthly reporting of HIV testing? We are primarily interested in the source document that is used for compiling monthly summary statistics for HIV test positive clients. Please report if any locally developed documents are used.	OPD register……1<br>IPD register……2<br>VCT tally sheet ……3<br>PIHTC tally sheets ____4<br>PEP register……8<br>Other (specify) ________96		
**REVIEW THE SOURCE DOCUMENTS USED TO COMPILE AND SUMMARIZE INFORMATION FOR MONTHLY REPORTING FOR HIV TESTING AND ANSWER THE FOLLOWING QUESTIONS**				

Supplement to: Worku AG, Midekssa WD, Tilahun HA, et al. The impact of health information system interventions on maternal and child health service utilizations in Ethiopia: quasi-experimental study. *Glob Health Sci Pract*. 2024;12(6):2400145. https://doi.org/10.9745/GHSP-D-24-00145

**FQ_050**	Please confirm the availability of **source documents** for **HIV testing** for month 1 to month 3. If available, please **Recount** the number of HIV tested positive clients recorded in the **source document** for month 1 to month 3	**(A) Source documents available**				**(B) Recount number of patients who tested positive for HIV in source document (if none, please enter 0)**
		**Yes, available and complete***	**Yes, available but partly** complete**	**Yes, available but no data recorded**	**No**	
**01**	**Month 1**	1	2	3	0	☐☐☐☐
**02**	**Month 2**	1	2	3	0	☐☐☐☐
**03**	**Month 3**	1	2	3	0	☐☐☐☐

Take the last 15 entries recorded in the VCT register for each reporting period and check if all the data elements relevant to the selected indicator are filled in.

***COMPLETE means that the source document contains the data relevant to the selected indicator. **PARTLY: the register is available but some information is missing.**

FQ_051	If the source document (VCT register/PIHTC tally sheet) is not available, what are the possible reasons?	Storage or archiving problems ....1<br>Absence of designated staff..............................2<br>Stock out of source document ....3<br>Other (specify): ______________________96
FQ_052	If the source documents (VCT register/PIHTC tally sheet) are partially complete or has no data, what are the possible reasons for the missing data?	Staffing issue(s)(shortage, absence)...........................1<br>Not understanding the data element ....2<br>Presence of other vertical reporting requirement ....3<br>Data burden (too much data elements to be recorded) 4<br>The recording tool is not designed as user friendly ....5<br>Other (specify): ______________________96

REVIEW THE MONTHLY REPORTS FOR HIV TESTING AND ANSWER THE FOLLOWING QUESTIONS.

**FQ_053**	Please confirm the availability of **monthly reports** for month 1 to month 3. If available, please **report** the number of HIV tested positive clients recorded in the **monthly reports** for month 1 to month 3.	**(A) Monthly reports available**				**(B) Record the number of patients who tested positive for HIV from the monthly report (if missing, leave blank)**
		**Yes, available and complete***	**Yes, available but partly** complete**	**Yes, available but no data recorded**	**No**	

Supplement to: Worku AG, Midekssa WD, Tilahun HA, et al. The impact of health information system interventions on maternal and child health service utilizations in Ethiopia: quasi-experimental study. *Glob Health Sci Pract*. 2024;12(6):2400145. https://doi.org/10.9745/GHSP-D-24-00145

**01**	**Month 2**	1	2	3	0	
**02**	**Month 3**	1	2	3	0	
**03**	**Month 2**	1	2	3	0	

***COMPLETE means that the monthly report contains the data relevant to the selected indicator.**
****PARTLY: the monthly report is available but some information is missing.**

DATA COMPLETENESS		
FQ_054	If the monthly report for HIV testing is not available, what are the possible reasons?	Storage or archiving problems ....1<br>Absence of designated staff........................2<br>Stock out of source document ....3<br>Other (specify): ____________________96
FQ_055	If the monthly report of HIV testing is partially complete or has no data, what are the possible reasons for the missing data?	Staffing issue(s)(shortage, absence) ....1<br>Not understanding the data element ....2<br>Presence of other vertical reporting requirement ....3<br>Data burden (too much data elements to be recorded) ....4<br>The recording tool is not designed as user friendly ....5<br>There is no client to be reported ....6<br>Other (specify): ____________________96

DISCREPANCIES		
**FQ_056**	If there was a discrepancy observed between the **source document** and the **monthly report**, what are the reasons for the discrepancy?	Data entry errors.................................1<br>Arithmetic errors.................................2<br>Information from all source documents not compiled correctly ....3<br>Data burden (too much data elements to be reported) ....4<br>Illegible writing on the source document (not readable) ....5<br>Lack of emphasis for data accuracy....................6<br>Other (specify) ______________96

**5. MALARIA INDICATOR**				
**NUMBER OF CONFIRMED MALARIA CASES**				
**FQ_057**	Does this facility diagnose and treat malaria?	1. Yes ☐	0. No ☐	➔FQ_068 (if No)
**SOURCE DOCUMENTS AND REPORTS**				
**FQ_058**	Does this facility report malaria data to a reporting system?	1. Yes ☐	0. No ☐	➔FQ_068 (if No)

Supplement to: Worku AG, Midekssa WD, Tilahun HA, et al. The impact of health information system interventions on maternal and child health service utilizations in Ethiopia: quasi-experimental study. *Glob Health Sci Pract*. 2024;12(6):2400145. https://doi.org/10.9745/GHSP-D-24-00145

**FQ_059**	To which of the following reporting systems does the facility report malaria data? (Please check the monthly reporting forms used by the health facility)			
**A**	Health Management Information System (HMIS)	1. Yes ☐	0. No ☐	
**B**	Malaria program	1. Yes ☐	0. No ☐	
**C**	Nongovernmental organizations or institutions	1. Yes ☐	0. No ☐	
**D**	Other reporting system	1.Yes ☐ ____________ SPECIFY	0. No ☐	

**FQ_060**	What is the source document used by this facility for monthly reporting of malaria? We are primarily interested in the source document that is used for compiling monthly summary statistics for malaria. Please report if any locally developed documents are used.	Laboratory register ....1<br>Other (specify) ____________________96

REVIEW THE SOURCE DOCUMENTS USED TO COMPILE AND SUMMARIZE INFORMATION FOR MONTHLY REPORTING FOR MALARIA AND ANSWER THE FOLLOWING QUESTIONS

**FQ_061**	Please confirm the availability of **source documents** for malaria for month 1 to month 3. If available, please **Recount** the number of conf. malaria cases recorded in the **source document** month 1 to month 3.	**(A) Source documents available**				**(B) Recount the number of conf. malaria cases in the source documents (if none, please enter 0)**
		**Yes, available and complete***	**Yes, available but partly** complete**	**Yes, available but no data recorded**	**No**	
**01**	**Month 1**	1	2	3	0	☐☐☐☐
**02**	**Month 2**	1	2	3	0	☐☐☐☐
**03**	**Month 3**	1	2	3	0	☐☐☐☐

Take the last 15 entries recorded in the Lab register for each reporting period and check if all the data elements relevant to the selected indicator are filled in.

***COMPLETE means that the source document contains the data relevant to the selected indicator. **PARTLY: the register is available but some information is missing.**

FQ_062	If the source document (Lab register) is not available, what are the possible reasons?	Storage or archiving problems ....1<br>Absence of designated staff........................2<br>Stock out of source document ....3<br>Other (specify): ____________________96

Supplement to: Worku AG, Midekssa WD, Tilahun HA, et al. The impact of health information system interventions on maternal and child health service utilizations in Ethiopia: quasi-experimental study. *Glob Health Sci Pract*. 2024;12(6):2400145. https://doi.org/10.9745/GHSP-D-24-00145

**FQ_063**	If the source documents (Lab register/) are partially complete or has no data, what are the possible reasons for the missing data?	Staffing issue(s)(shortage, absence)........1<br>Not understanding the data element ....2<br>Presence of other vertical reporting requirement ....3<br>Data burden (too much data elements to be recorded) 4<br>The recording tool is not designed as user friendly ....5<br>Other (specify): ____________96

REVIEW THE MONTHLY REPORTS FOR CONFIRMED MALARIA AND ANSWER THE FOLLOWING QUESTIONS.

**FQ_064**	Please confirm the availability of **monthly reports** for month 1 to month 3. If available, please **report** the number of # of conf. malaria cases recorded in the **monthly reports** for month 1 to month 3.	**(A) Monthly reports available**				**(B) Record the number of # of conf. malaria cases from the monthly reports (if missing, leave blank)**
		**Yes, available and complete***	**Yes, available but partly** complete**	**Yes, available but no data recorded**	**No**	
**01**	**Month 1**	1	2	3	0	☐☐☐☐
**02**	**Month 2**	1	2	3	0	☐☐☐☐
**03**	**Month 3**	1	2	3	0	☐☐☐☐

***COMPLETE means that the monthly report contains the data relevant to the selected indicator.**

****PARTLY: the monthly report is available but some information is missing.**

DATA COMPLETENESS

FQ_065	If the monthly report for malaria is not available, what are the possible reasons?	Storage or archiving problems ....1<br>Absence of designated staff........2<br>Stock out of source document ....3<br>Other (specify): ____________96
FQ_066	If the monthly report of malaria is partially complete or has no data, what are the possible reasons for the missing data?	Staffing issue(s)(shortage, absence) ....1<br>Not understanding the data element ....2<br>Presence of other vertical reporting requirement ....3<br>Data burden (too much data elements to be recorded) ....4<br>The recording tool is not designed as user friendly ....5<br>There is no client to be reported ....6<br>Other (specify): ____________96

DISCREPANCIES

Supplement to: Worku AG, Midekssa WD, Tilahun HA, et al. The impact of health information system interventions on maternal and child health service utilizations in Ethiopia: quasi-experimental study. *Glob Health Sci Pract*. 2024;12(6):2400145. https://doi.org/10.9745/GHSP-D-24-00145

**FQ_067**	If there was a discrepancy observed between the **source document** and the **monthly report**, what are the reasons for the discrepancy?	Data entry errors_______________________1<br>Arithmetic errors_______________________2<br>Information from all source documents not compiled correctly ……………………………………………………3<br>Data burden (too much data elements to be reported) ……………………………………………………4<br>Illegible writing on the source document (not readable) ……………………………………………………5<br>Lack of emphasis for data accuracy…………………6<br>Other (specify) ______________________96

	**6. DISEASE IN UNDER FIVE CHILDREN INDICATOR**			
	**NUMBER OF UNDER FIVE CHILDREN WITH PNEUMONIA**			
**FQ_068**	Does this facility provide pneumonia treatment service?	1. Yes ☐	0. No ☐	➔FQ_079 (if No)
**SOURCE DOCUMENTS AND REPORTS**				
**FQ_069**	Does this facility report pneumonia data to a reporting system?	1. Yes ☐	0. No ☐	➔FQ_079 (if No)
**FQ_070**	To which of the following reporting systems does the facility report pneumonia data? (please check the monthly reporting forms used by the health facility)			
**A**	Health Management Information System (HMIS)	1. Yes ☐	0. No ☐	
**B**	IMNCI program	1. Yes ☐	0. No ☐	
**C**	Nongovernmental organizations or institutions	1. Yes ☐	0. No ☐	
**D**	Other reporting system	7. Yes ☐<br>__________<br>SPECIFY	0. No ☐	
**FQ-071**	What is the source document used by this facility for monthly reporting of pneumonia? We are primarily interested in the source document that is used for compiling monthly summary statistics for Pneumonia. Please report if any locally developed documents are used.	IMNCI register _____________________1<br>OPD register……………………………2<br>IPD register………………………………3<br>Disease tally sheets ________________…4<br>Other (specify) ______________________96		
**REVIEW THE SOURCE DOCUMENTS USED TO COMPILE AND SUMMARIZE INFORMATION FOR MONTHLY REPORTING FOR PNEUMONIA AND ANSWER THE FOLLOWING QUESTIONS**				

Supplement to: Worku AG, Midekssa WD, Tilahun HA, et al. The impact of health information system interventions on maternal and child health service utilizations in Ethiopia: quasi-experimental study. *Glob Health Sci Pract*. 2024;12(6):2400145. https://doi.org/10.9745/GHSP-D-24-00145

**FQ_072**	Please confirm the availability of **source documents** for pneumonia in under-five for month 1 to month 3. If available, please **Recount** the number of under-five children with pneumonia recorded in the **source document** for month 1 to month 3.	**(A) Source documents available**				**(B) Recount the number of under-five children with pneumonia in the source documents (if none, please enter 0)**
		**Yes, available and complete***	**Yes, available but partly** complete**	**Yes, available but no data recorded**	**No**	
**01**	**Month 1**	1	2	3	0	☐☐☐☐
**02**	**Month 2**	1	2	3	0	☐☐☐☐
**03**	**Month 3**	1	2	3	0	☐☐☐☐

Take the last 15 entries recorded in the two IMNCI registers (for children <2 months and 2 months to 5 years) for each reporting period and check if all the data elements relevant to the selected indicator are filled in. If there is no IMNCI register, use OPD register in under five clinic/pediatric OPD units.

***COMPLETE means that the source document(s) contain the data relevant to the selected indicator. **PARTLY: the register is available but some information is missing.**

FQ_073	If the source document (IMNCI register/ OPD tally sheet) is not available, what are the possible reasons?	Storage or archiving problems ....1<br>Absence of designated staff..........2<br>Stock out of source document ....3<br>Other (specify): ____________96
**FQ_074**	If the source documents (IMNCI register/ OPD tally sheet) are partially complete or has no data, what are the possible reasons for the missing data?	Staffing issue(s)(shortage, absence)..........1<br>Not understanding the data element ....2<br>Presence of other vertical reporting requirement ....3<br>Data burden (too much data elements to be recorded) ....4<br>The recording tool is not designed as user friendly ....5<br>Other (specify): ____________96

REVIEW THE MONTHLY REPORTS FOR PNEUMONIA IN UNDER_FIVE CHILDREN AND ANSWER THE FOLLOWING QUESTIONS.

**FQ_075**	Please confirm the availability of **monthly reports** for mont h1 to month 3. If available, please **report** the number under-five children with pneumonia recorded in the **monthly reports** for month 1 to month 3.<br>.	**(A) Monthly reports available**	**(B) Record the number of under-five children with pneumonia from the monthly reports (if missing, leave blank)**

		Yes, available and complete*	Yes, available but partly** complete	Yes, available but no data recorded	No	
**01**	**Month 2**	1	2	3	4	
**02**	**Month 3**	1	2	3	4	
**03**	**Month 2**	1	2	3	4	

***COMPLETE means that the monthly report contains the data relevant to the selected indicator.**

****PARTLY: the monthly report is available but some information is missing.**

DATA COMPLETENESS		
FQ_076	If the monthly report for pneumonia in under-five children is not available, what are the possible reasons?	Storage or archiving problems ....1<br>Absence of designated staff................................2<br>Stock out of source document ....3<br>Other (specify): ________________________96
FQ_077	If the monthly report of pneumonia in under-five children is partially complete or has no data, what are the possible reasons for the missing data?	Staffing issue(s)(shortage, absence) ....1<br>Not understanding the data element ....2<br>Presence of other vertical reporting requirement ....3<br>Data burden (too much data elements to be recorded) ....4<br>The recording tool is not designed as user friendly ....5<br>There is no client to be reported ....6<br>Other (specify): ________________________96
**DISCREPANCIES**		
**FQ_078**	If there was a discrepancy observed between the **source document** and the **monthly report**, what are the reasons for the discrepancy?	Data entry errors..1<br>Arithmetic errors...2<br>Information from all source documents not compiled correctly ....3<br>Data burden (too much data elements to be reported) ....4<br>Illegible writing on the source document (not readable) ....5<br>Lack of emphasis for data accuracy.....................6<br>Other (specify) ____________________96

7. HOSPITAL/HEALTH CENTER SERVICE QUALITY INDICATOR

NUMBER OF INPATIENT DEATHS

Supplement to: Worku AG, Midekssa WD, Tilahun HA, et al. The impact of health information system interventions on maternal and child health service utilizations in Ethiopia: quasi-experimental study. *Glob Health Sci Pract*. 2024;12(6):2400145. https://doi.org/10.9745/GHSP-D-24-00145

**FQ_079**	Does this facility provide admission services?	1. Yes ☐	0. No ☐	➔FQ_090 (if No)
**SOURCE DOCUMENTS AND REPORTS**				
**FQ_080**	Does this facility report inpatient deaths data to a reporting system?	1. Yes ☐	0. No ☐	➔FQ_090 (if No)
**FQ_081**	To which of the following reporting systems does the facility report inpatient deaths data?<br>(Please check the monthly reporting form used by the health facility)			
**A**	Health Management Information System (HMIS)	1. Yes ☐	0. No ☐	
**B**	Hospital Quality Improvement program	1. Yes ☐	0. No ☐	
**C**	Nongovernmental organizations or institutions	1. Yes ☐	0. No ☐	
**D**	Other reporting system	1. Yes ☐<br>____________<br>SPECIFY	0. No ☐	
**FQ_082**	What is the source document used by this facility for monthly reporting of inpatient deaths? We are primarily interested in the source document that is used for compiling monthly summary statistics for inpatient deaths. Please report if any locally developed documents are used.	IPD register ....1<br>IPD disease tally sheets ....2<br>NICU register ....3<br>Other (specify) ______________96		

REVIEW THE SOURCE DOCUMENTS USED TO COMPILE AND SUMMARIZE INFORMATION FOR MONTHLY REPORTING FOR INPATIENT DEATHS AND ANSWER THE FOLLOWING QUESTIONS

**FQ_083**	Please confirm the availability of **source documents** for inpatient deaths for month 1 to month 3. If available, please **Recount** the number of inpatient deaths recorded in the **main source document** for month 1 to month 3.	**(A) Source documents available**				**(B) Recount the number of inpatient deaths in the source documents (if none, please enter 0)**
		**Yes, available and complete***	**Yes, available but partly** complete**	**Yes, available but no data recorded**	**No**	
**01**	**Month 1**	1	2	3	0	☐☐☐☐
**02**	**Month 2**	1	2	3	0	☐☐☐☐
**03**	**Month 3**	1	2	3	0	☐☐☐☐

Take the last 15 entries recorded in the IPD registers for each reporting period and check if all the data elements relevant to the selected indicator are filled in.

***COMPLETE means that the source document contains the data relevant to the selected indicator. **PARTLY: the register is available but some information is missing.**

Supplement to: Worku AG, Midekssa WD, Tilahun HA, et al. The impact of health information system interventions on maternal and child health service utilizations in Ethiopia: quasi-experimental study. *Glob Health Sci Pract*. 2024;12(6):2400145. https://doi.org/10.9745/GHSP-D-24-00145

FQ_084	If the source document (IPD register/ IPD tally sheet) is not available, what are the possible reasons?	Storage or archiving problems ......1<br>Absence of designated staff......2<br>Stock out of source document ......3<br>Other (specify): ________96
**FQ_085**	If the source documents (IPD register/ IPD tally sheet) are partially complete or have no data, what are the possible reasons for the missing data?	Staffing issue(s)(shortage, absence)......1<br>Not understanding the data element ......2<br>Presence of other vertical reporting requirement ......3<br>Data burden (too much data elements to be recorded) 4<br>The recording tool is not designed as user friendly ......5<br>Other (specify): ________96

REVIEW THE MONTHLY REPORTS FOR INPATIENT DEATHS AND ANSWER THE FOLLOWING QUESTIONS.

**FQ_086**	Please confirm the availability of **monthly reports** for month 1 to month 3. If available, please **report** the number inpatient deaths recorded in the **monthly reports** for month 1 to month 3.	**(A) Monthly reports available**				**(B) Record the number of inpatient deaths from the monthly reports (if missing, leave blank)**
		**Yes, available and complete***	**Yes, available but partly** complete**	**Yes, available but no data recorded**	**No**	
**01**	**Month 2**	1	2	3	0	☐☐☐☐
**02**	**Month 3**	1	2	3	0	☐☐☐☐
**03**	**Month 2**	1	2	3	0	☐☐☐☐

***COMPLETE means that the monthly report contains the data relevant to the selected indicator.**

****PARTLY: the monthly report is available but some information is missing.**

DATA COMPLETENESS

FQ_087	If the monthly report for inpatient deaths is not available, what are the possible reasons?	Storage or archiving problems ......1<br>Absence of designated staff......2<br>Stock out of source document ......3<br>Other (specify): ________96
FQ_088	If the monthly report of inpatient deaths is partially complete or has no data, what are the possible reasons for the missing data?	Staffing issue(s)(shortage, absence) ......1<br>Not understanding the data element ......2<br>Presence of other vertical reporting requirement ......3<br>Data burden (too much data elements to be recorded) ......4<br>The recording tool is not designed as user friendly ......5<br>There is no client to be reported ......6<br>Other (specify): ________96

Supplement to: Worku AG, Midekssa WD, Tilahun HA, et al. The impact of health information system interventions on maternal and child health service utilizations in Ethiopia: quasi-experimental study. *Glob Health Sci Pract*. 2024;12(6):2400145. https://doi.org/10.9745/GHSP-D-24-00145

DISCREPANCIES		
**FQ_089**	If there was a discrepancy observed between the **source document** and the **monthly report**, what are the reasons for the discrepancy?<br><br>[ask the person that compiles and prepares the report or the HMIS department head]	Data entry errors……1<br>Arithmetic errors……2<br>Information from all source documents not compiled correctly ……3<br>Data burden (too much data elements to be reported) ……4<br>Illegible writing on the source document (not readable) ……5<br>Lack of emphasis for data accuracy……6<br>Other (specify) ________96

8. TB INDICATOR

NUMBER OF TB CASES (ALL TYPES)				
**FQ_090**	Does this facility provide TB diagnosis and/or treatment?	1. Yes ☐	0. No ☐	➔FQ_204 (if No)
**SOURCE DOCUMENTS AND REPORTS**				
**FQ_091**	Does this facility report the **total number of TB cases (all types)** to a reporting system?	1. Yes ☐	0. No ☐	➔FQ_204 (if No)
**FQ_092**	To which of the following reporting systems does the facility report the **total number of TB cases (all types)**?			
**A**	Health Management Information System	1. Yes ☐	0. No ☐	
**B**	National TB program	1. Yes ☐	0. No ☐	
**C**	Nongovernmental organizations or institutions	1. Yes ☐	0. No ☐	
**D**	Other reporting system	1. Yes ☐<br>________<br>SPECIFY	0. No ☐	
**FQ_093**	What is the source document used by this facility for quarterly reporting of notified TB cases? We are primarily interested in the source document that is used for **compiling** quarterly summary statistics for the **total number of TB cases (all types)**. Please report if any locally developed documents are used.	UNIT TB register ……1<br>Other (specify) ________96		
**REVIEW THE SOURCE DOCUMENTS USED TO COMPILE AND SUMMARIZE INFORMATION FOR MONTHLY REPORTING FOR TB CASES AND ANSWER THE FOLLOWING QUESTIONS**				

Supplement to: Worku AG, Midekssa WD, Tilahun HA, et al. The impact of health information system interventions on maternal and child health service utilizations in Ethiopia: quasi-experimental study. *Glob Health Sci Pract*. 2024;12(6):2400145. https://doi.org/10.9745/GHSP-D-24-00145

**FQ_094**	Please confirm the availability of the **source document** used at the facility to compile the number of **notified cases of TB** for the quarter (Month 1 to month 3). If available, please **recount** and record the number of **notified cases of TB** as recorded in the **main source document** for the quarter (month1 to month 3).	**(A) Source documents available**				**(B) Recount the number of notified cases of TB in the source documents (if none, please enter 0)**
		**Yes, available and complete***	**Yes, available but partly** complete**	**Yes, available but no data recorded**	**No**	
**01**	**Quarter (Month 1 to Month 3)**	1	2	3	0	☐☐☐☐

Take the last 10 entries recorded in the Unit TB registers for each reporting period and check if all the data elements relevant to the selected indicator are filled in.

***COMPLETE means that the source document contains the data relevant to the selected indicator. **PARTLY: the register is available but some information is missing.**

**FQ_097**	If the source document (TB unit register) is not available, what are the possible reasons?	Storage or archiving problems ____1<br>Absence of designated staff____2<br>Stock out of source document ____3<br>Other (specify): ____96
**FQ_098**	If the source documents (TB unit register) are partially complete or have no data, what are the possible reasons for the missing data?	Staffing issue(s)(shortage, absence)____1<br>Not understanding the data element ____2<br>Presence of other vertical reporting requirement ____3<br>Data burden (too much data elements to be recorded)<br>4<br>The recording tool is not designed as user friendly ____5<br>Other (specify): ____96
**FQ_095**	From the TB register, count the total number of TB cases **that were transferred in** for the verification period (month1 to month 3). Please record the total number for the quarter.<br><br>**(If none, please enter 0.)**	**(B) =** ☐☐☐☐
**FQ_096**	**CALCULATE C: TOTAL NUMBER OF TB CASES FROM THE TB REGISTER MINUS THE TRANSFERRED-IN CASES. (*TRANSFERRED-IN CASES ARE NOT INCLUDED IN THE RECEIVING UNIT'S CASE REGISTRATIONS.*)**	**TB CASES THAT SHOULD BE REPORTED**<br><br>**C = A – B =** ☐☐☐☐

REVIEW THE QUARTERLY REPORTS FOR TB CASES AND ANSWER THE FOLLOWING QUESTIONS.

Supplement to: Worku AG, Midekssa WD, Tilahun HA, et al. The impact of health information system interventions on maternal and child health service utilizations in Ethiopia: quasi-experimental study. *Glob Health Sci Pract*. 2024;12(6):2400145. https://doi.org/10.9745/GHSP-D-24-00145

**FQ_099**	Please confirm the availability of the **quarterly report** for the number of **notified cases of TB** for month1 to month 3. If available, please **record** the number of **notified cases of TB** as recorded in the **quarterly report** for month1 to month 3.	**(A) Quarterly reports available**				**(B) Record the number of notified cases of TB in the quarterly report (if missing in the quarterly report, leave blank)**
		**Yes, available and complete***	**Yes, available but partly** complete**	**Yes, available but no data recorded**	**No**	
	**01. Quarterly report for month 1 to month 3**	1	2	3	0	D= ☐☐☐☐

***COMPLETE means that the monthly report contains the data relevant to the selected indicator.**

****PARTLY: the monthly report is available but some information is missing.**

DATA COMPLETENESS

FQ_200	If the quarterly report for notified TB cases is not available, what are the possible reasons?	Storage or archiving problems ....1<br>Absence of designated staff....................2<br>Stock out of source document ....3<br>Other (specify): ____________________96
FQ_201	If the quarterly report of notified TB cases is partially complete or has no data, what are the possible reasons for the missing data?	Staffing issue(s)(shortage, absence) ....1<br>Not understanding the data element ....2<br>Presence of other vertical reporting requirement ....3<br>Data burden (too much data elements to be recorded) ....4<br>The recording tool is not designed as user friendly ....5<br>There is no client to be reported ....6<br>Other (specify): ____________________96

DISCREPANCIES

FQ_202	**CALCULATE THE DIFFERENCE BETWEEN C (from FQ_098) AND D (from FQ_099)**	**C – D =** ☐☐☐☐	➔FQ_204 (if 0)
FQ_203	If there was a discrepancy observed between the source document and the quarterly report, what are the reasons for the discrepancy?	Data entry errors....................1<br>Arithmetic errors....................2<br>Information from all source documents not compiled correctly ....3<br>Data burden (too much data elements to be reported) ....4<br>Illegible writing on the source document (not readable) ....5<br>Lack of emphasis for data accuracy....................6<br>Other (specify) ____________________96	

Supplement to: Worku AG, Midekssa WD, Tilahun HA, et al. The impact of health information system interventions on maternal and child health service utilizations in Ethiopia: quasi-experimental study. *Glob Health Sci Pract*. 2024;12(6):2400145. https://doi.org/10.9745/GHSP-D-24-00145

REPORT TIMELINESS					
FQ_204	A. Is there a deadline for submission of the HMIS report by the health facilities?		1. Yes ☐	0. No ☐	➔FQ 205 (if No)
	B. If yes, what is the deadline (date of month)?<br>(Write the end date of the deadline)		Reporting deadline:<br>---------------------------------		
FQ-205	Does the health facility record the dates of submission of monthly HMIS reports to the Woreda/Zone/Region (see logbook/computer)?		1. Yes ☐	0. No ☐	➔FQ_207 (if No)
	**IF AVAILABLE, REVIEW THE RECORDS AND CHECK THE DATES OF SUBMISSION FOR THE THREE REVIEW MONTHS**				
		**A. Service**	**B. IPD**	**C. OPD**	
FQ_206_01	Were the following HMIS monthly reports submitted on time in Month 1?<br>**(Current practices is submission on or before 26th of the month)**	1. Yes ☐<br>0. No ☐	1. Yes ☐<br>0. No ☐	1. Yes ☐<br>0. No ☐	
FQ_206_02	Were the following HMIS monthly reports submitted on time in Month 2?<br>**(Current practices is submission on or before 26th of the month)**	1. Yes ☐<br>0. No ☐	1. Yes ☐<br>0. No ☐	1. Yes ☐<br>0. No ☐	
FQ_206_03	Were the following HMIS monthly reports submitted on time in Month 3?<br>**(Current practices is submission on or before 26th of the month)**	1. Yes ☐<br>0. No ☐	1. Yes ☐<br>0. No ☐	1. Yes ☐<br>0. No ☐	

DATA QUALITY ASSESSMENT MECHANISM				
FQ_207.	Does the health facility have data quality self-assessment tools (e.g. LQAS tool, electronic data quality validation rule/system)?	1. Yes, observed ☐	0. No ☐	➔FQ_212 (if No)
FQ_208.	Did the health facility conduct LQAS in the review three months?<br>(Please observe)	1. Yes, observed ☐	0. No ☐	➔FQ_212 (if No)

Supplement to: Worku AG, Midekssa WD, Tilahun HA, et al. The impact of health information system interventions on maternal and child health service utilizations in Ethiopia: quasi-experimental study. *Glob Health Sci Pract*. 2024;12(6):2400145. https://doi.org/10.9745/GHSP-D-24-00145

FQ_209.	If Yes, which types of the report are covered by the LQAS?			
	**Types of Reports**	**Month 1**	**Month 2**	**Month 3**
	01. For service report	1. Yes ☐<br>0. No ☐	1. Yes ☐<br>0. No ☐	1. Yes ☐<br>0. No ☐
	02. For disease report	1. Yes ☐<br>0. No ☐	1. Yes ☐<br>0. No ☐	1. Yes ☐<br>0. No ☐
FQ_210.	Does the health facility maintain a record of LQAS check sheets conducted in the review three months?	1. Yes, observed ☐	0. No ☐	
FQ_211.	Does the health facility maintain records of feedback to staff on data quality self-assessment findings?	1. Yes, observed ☐	0. No ☐	

DATA PROCESSING AND ANALYSIS

FQ_212.	Does the health facility use an electronic database/system for routine health data management?	1. Yes ☐	0. No ☐	➔FQ_2 14 (if No)

FQ_213. If yes, indicate the type of electronic system used for routine health data management.

**Electronic system**	**A. For data entry**		**B. For data analysis**	
	**1. Yes**	**0. No**	**1. Yes**	**0. No**
01. National open-source data processing system (e.g., DHIS 2)				
02 National proprietary software (e.g., e-HMIS)				
03. Excel-based spreadsheet				
04. Access-based data processing module				
05. Other (specify)				

FQ_214.	**ASK RELEVANT STAFF IN THE HEALTH FACILITY OFFICE TO SHOW UP-TO-DATE (I.E., NOT MORE THAN ONE YEAR OLD) REPORTS, DOCUMENTS, AND/OR DISPLAYS THAT CONTAIN THE FOLLOWING. THE ENUMERATOR SHOULD RECORD THE OBSERVATIONS ACCORDINGLY.**		
A.	Aggregated/summary HMIS report	1. Yes, observed ☐	0. No ☐

B.	Demographic data on the catchment population of the health facility for calculating coverages.	1. Yes, observed ☐	0. No ☐
C.	Indicators (e.g., Penta3 coverage) calculated for the health facility catchment area within the review three months.	1. Yes, observed ☐	0. No ☐
D.	Comparisons between health facility and Woreda/national targets.	1. Yes, observed ☐	0. No ☐
E.	Comparisons of data over time, i.e., monitoring trends (e.g., for ANC, Penta3).	1. Yes, observed ☐	0. No ☐
F.	Comparisons of sex-disaggregated data (e.g., for Penta3, HIV testing, PIHCT).	1. Yes, observed ☐	0. No ☐
G.	Comparisons of service coverage between associated services (e.g., ANC, TT immunization, SBA).	1. Yes, observed ☐	0. No ☐

This section is applicable only for the health centers that have health post under their supervision		
**COMPLETENESS OF HEALTH POSTS REPORTING TO THE HEALTH CENTER**		
FQ_215.	Does this health facility have at least one health post under its supervision?<br>**If No, skip to FU_003**	Yes______1<br>No ______0
FQ_216.	Does the health center keep log book/electronic database to track monthly HMIS reports sent by the health posts? (observe)	Yes, paper-based only______1<br>Yes, electronic only______2<br>Yes, both paper-based and electronic systems__3<br>No ______0
FQ_217.	Does the health center keep copies of monthly HMIS reports (paper-based or electronic) sent by the health posts? (observe)<br>**(Check the reports for the three review months)**	Yes, paper-based only______1<br>Yes, electronic only______2<br>Yes, both paper-based and electronic copies (all health posts submit both types of reports)…3<br>Yes, mixed (some health posts submit paper-based reports; others submit electronic reports)..4<br>No______0
FQ_218.	How many health posts are supposed to submit the monthly HMIS report (service and disease) to the health center and by what method?	

**Health facility type***<br>**(*Specify the facility type according to the structure of the country's health system.)**	**A. Paper-based report only**			**B. Electronic report only**			**C. Both paper and electronic reports**		
	**Service**	**Disease**	**Quarterly**	**Service**	**Disease**	**Quarterly**	**Service**	**Disease**	**Quarterly**

Supplement to: Worku AG, Midekssa WD, Tilahun HA, et al. The impact of health information system interventions on maternal and child health service utilizations in Ethiopia: quasi-experimental study. *Glob Health Sci Pract*. 2024;12(6):2400145. https://doi.org/10.9745/GHSP-D-24-00145

05. Health posts									

FQ_219. **CHECK THE MONTHLY HMIS REPORTS SUBMITTED BY THE HEALTH POSTS DURING THE REVIEW PERIOD**

How many health posts actually submitted monthly HMIS (service and disease) reports for:

A. Month 1 __________ year____________?

Health facility type* (*Specify the facility type according to the structure of the country's health system.)	A. Paper-based report only		B. Electronic report only		C. Both paper and electronic reports	
	Service	Disease	Service	Disease	Service	Disease
01. Health posts						

B. Month 2 __________ year____________?

Health facility type	A. Paper-based report only		B. Electronic report only		C. Both paper and electronic reports	
	Service	Disease	Service	Disease	Service	Disease
01. Health posts						

C. Month 3 __________ year____________?

Health facility type	A. Paper-based report only			B. Electronic report only			C. Both paper and electronic reports		
	Service	Disease	Quarterly	Service	Disease	Quarterly	Service	Disease	Quarterly
01. Health posts									

FQ_220. If health posts are not submitting monthly HMIS reports, what are the possible reasons for this?

Staffing issue(s)........1
Lack of reporting supplies........2
Transportation issue(s)........3
Internet connectivity issue(s)........4
Presence of other vertical reporting requirements 5
Computer system failure........6
Electric supply interruption........7

Other (specify) ____________96

Supplement to: Worku AG, Midekssa WD, Tilahun HA, et al. The impact of health information system interventions on maternal and child health service utilizations in Ethiopia: quasi-experimental study. *Glob Health Sci Pract*. 2024;12(6):2400145. https://doi.org/10.9745/GHSP-D-24-00145

**REPORT TIMELINESS**				
FQ_221.	A. Is there a deadline for submission of the monthly HMIS report by the health posts?	1. Yes ☐	0. No ☐	➔FU_003 (if No)
	B. If yes, what is the deadline (date of month)?<br>(Write the end date of deadline)	Reporting deadline:<br>______________________		
FQ_222.	Does the health center record receipt dates of monthly CHIS reports (observe logbook/electronic system)?	1. Yes ☐	0. No ☐	➔FU_003 (if No)
FQ_223.	Check the receipt dates for the three review months. How many reports were received on time?			

Health facility type	A. Paper-based report only		B. Electronic report only		C. Both paper and electronic reports	
	Service	Disease	Service	Disease	Service	Disease
01. Health posts						

Supplement to: Worku AG, Midekssa WD, Tilahun HA, et al. The impact of health information system interventions on maternal and child health service utilizations in Ethiopia: quasi-experimental study. *Glob Health Sci Pract*. 2024;12(6):2400145. https://doi.org/10.9745/GHSP-D-24-00145

Part 2. Use of Information: Health Facility Assessment Form

DATA VISUALIZATION				
FU_003	Does the health facility prepare data visuals (graphs, tables, maps, etc.) showing achievements toward targets (indicators, geographic and/or temporal trends, and situation data)?	Yes, all paper or electronic copies of data visuals observed at the health facility .... 1<br>Yes, some paper or electronic copies of data visuals observed at the health facility .... 2<br>No 0		➔FU_005 (if No)
FU_004	If yes, what type of information is captured in the data visuals and is it updated as per the standard?			
A	Map of catchment area	1. Yes, observed ☐	0. No ☐	
B	Catchment population profile	1. Yes, observed ☐	0. No ☐	
C	Staffing	1. Yes, observed ☐	0. No ☐	
D	Ten Top Causes of Morbidity (Males & Females)	1. Yes, observed ☐	0. No ☐	
E	Ten Top Causes of Morbidity In < 5 Children	1. Yes, observed ☐	0. No ☐	
F	ANC coverage	1. Yes, observed ☐	0. No ☐	
G	Skilled attendant deliveries	1. Yes, observed ☐	0. No ☐	
H	Penta-3 immunization coverage	1. Yes, observed ☐	0. No ☐	
I	Measles immunization coverage	1. Yes, observed ☐	0. No ☐	
J	Malaria, all ages	1. Yes, observed ☐	0. No ☐	
K	Pneumonia amongst Under 5 years	1. Yes, observed ☐	0. No ☐	
L	HIV/AIDS (VCT)	1. Yes, observed ☐	0. No ☐	
M	HIV/AIDS (PMTCT)	1. Yes, observed ☐	0. No ☐	
N	HIV/AIDS (ART)	1. Yes, observed ☐	0. No ☐	
O	Other (specify)	1. Yes, observed ☐	0. No ☐	

HMIS ANALYTIC REPORT PRODUCTION			
FU_005	Does the health facility have analyzed HMIS data (e.g., summary tables, charts, maps)?	Yes, observed paper-based........1<br>Yes, observed electronic........2<br>No........0	

Supplement to: Worku AG, Midekssa WD, Tilahun HA, et al. The impact of health information system interventions on maternal and child health service utilizations in Ethiopia: quasi-experimental study. *Glob Health Sci Pract*. 2024;12(6):2400145. https://doi.org/10.9745/GHSP-D-24-00145

FU_006	Does the health facility produce any report or bulletin (annual, quarterly, etc.) based on analysis of HMIS data?<br>**(Excluding the monthly summary/aggregate reports submitted to the higher level.)**	Yes, observed ....1<br>No 0		➔FU_009 (if No)
FU_007	If yes, list the reports, indicating the frequency of the reports and the number of times the reports were actually issued in the last 12<br>months			
	Title of the report/bulletin<br>**(A)**	Number of times this report is supposed to be issued per year<br>**(B)**	Number of times this report was actually issued in the last 12 months<br>**(C)**	Target audience of the report (e.g., FMOH, RHB, ZHD, civil administration, parliament, community forums, general population)<br>**(D)**
01				
02				
03				
FU_008	Do any of these reports and/or bulletins contain discussions and decisions/recommendations based on key performance targets based on HMIS data, such as:			
	01. Maternal and Child health	1. Yes ☐	0. No ☐	
	02. Health Promotion and Disease prevention	1. Yes ☐	0. No ☐	
	03. Public health emergency	1. Yes ☐	0. No ☐	
	04. Human resource management	1. Yes ☐	0. No ☐	
	05. Pharmaceuticals	1. Yes ☐	0. No ☐	
	06. Finance and Resource	1. Yes ☐	0. No ☐	
	07. Health Information System	1. Yes ☐	0. No ☐	

**FEEDBACK TO HEALTH FACILITIES**				
FU_009	Did the health facility receive feedback reports from the Region/Zone/Woreda health office based on HMIS information in the review three months?<br>***(OBSERVE THE REPORT AND TICK ACCORDINGLY.)***	1. Yes, observed ☐	0. No ☐	➔FU_011 (if No)
FU_010	If yes, indicate the types of feedback reports:			

Supplement to: Worku AG, Midekssa WD, Tilahun HA, et al. The impact of health information system interventions on maternal and child health service utilizations in Ethiopia: quasi-experimental study. *Glob Health Sci Pract*. 2024;12(6):2400145. https://doi.org/10.9745/GHSP-D-24-00145

A	Feedback on data quality (including data accuracy, reporting timeliness, and/or report completeness)	1. Yes, observed ☐	0. No ☐
B	Feedback on service performance based on reported HMIS data (e.g., appreciation/ acknowledgement of good performance; resource allocation/mobilization)	1. Yes, observed ☐	0. No ☐

ROUTINE DECISION-MAKING FORUMS AND PROCESSES AT HEALTH FACILITY								
FU_011	Does the health facility have performance monitoring team (PMT)?	1. Yes ☐		0 No ☐				➔FU_025 (if No)
FU_012	Is the PMT membership according to the standard? (Head of the institution, HMIS in charge and all representatives of the case teams)	1. Yes ☐		0. No ☐				
FU_013	How often are the performance monitoring team (PMT) meetings supposed to take place?	Weekly .... 1<br>Monthly .... 2<br>Quarterly .... 3<br>Biannually .... 4<br>Annually .... 5<br>No schedule 0						
FU_014	How many times did the performance monitoring team (PMT) meetings take place during the review three months?	More than four times .... 1<br>Four times .... 2<br>Three times .... 3<br>Two times .... 4<br>One time .... 5<br>None 0						➔FU_025 (if None)
FU_015	Were minutes of performance monitoring team meetings maintained for the review three months?	Month 1		Month 2		Month 3		➔FU_025 (if No)
		1. Yes ☐	0. No ☐	1. Yes ☐	0. No ☐	1. Yes ☐	0. No ☐	
FU_016	Is the PMT using a standard PMT minute record/logbook?<br><br>(Standard: Data Quality and Performance Monitoring logbook)	1. Yes, observed ☐		0. No ☐				
FU_017	Please indicate the reason(s) for not using the standard minute record/logbook.<br><br>(Select all that apply)	Standard logbook is not available .... 1<br>Staff not trained/familiar with the logbook .... 2<br>Logbook is not user friendly .... 3<br>Other (Specify): ____________ 96						
	Please check the performance monitoring team minute/records for the review threemonths to see who chaired the meetings each month.							
FU018_01	Who chaired the meetings in Month 1?	Facility in-charge/medical director of the health facility........ 1<br>HMIS in charge........ 2<br>Case team leaders/representatives 3						➔FU_020 (if "1" selected)
FU018_02	Who chaired the meetings in Month 2?	Facility in-charge/medical director of the health facility........ 1<br>HMIS in charge........ 2<br>Case team leaders/representatives 3						

Supplement to: Worku AG, Midekssa WD, Tilahun HA, et al. The impact of health information system interventions on maternal and child health service utilizations in Ethiopia: quasi-experimental study. *Glob Health Sci Pract*. 2024;12(6):2400145. https://doi.org/10.9745/GHSP-D-24-00145

FU018_03	Who chaired the meetings in Month 3?	Facility in-charge/medical director of the health facility……1<br>HMIS in charge……2<br>Case team leaders/representatives……3		
FU_019	Please indicate the reason(s) if the facility in-charge/Medical director of the health facility did not chair the meeting **(ask the HMIS focal person)**	Not available during the meeting……1<br>Other competing priorities……2<br>Lack of familiarity with the subject……3<br>Other (specify)……96		
FU_020	Please check the performance monitoring team minute/records for the review months and see if the following topics were discussed.			
A	Were there discussions on HMIS management, such as data quality, completeness, or timeliness of reporting?	1. Yes ☐	0. No ☐	→FU_021 (if No)
B	Were HMIS related issues identified and prioritized?	1. Yes ☐	0. No ☐	→FU_021 (if No)
C	Were root cause analysis conducted for the prioritized HMIS related issues?	1. Yes ☐	0. No ☐	
D	Was an action plan developed to address the HMIS related issues?	1. Yes ☐	0. No ☐	
E	Has any follow-up actions taken place based on the decisions made during the previous meetings on HMIS-related issues? (e.g., referring HMIS-related issues to the higher level)	1. Yes ☐	0. No ☐	
FU_021<br><br>A	In the PMT meetings, were discussions held to review the following key performance targets (e.g., tracking progress against targets/plan):<br><br>(Please check the performance monitoring team minute/records for the review months and see if the following topics were discussed)			→FU_022 (if all are No)
	01. Maternal and Child health	1. Yes ☐	0. No ☐	
	02. Health Promotion and Disease prevention	1. Yes ☐	0. No ☐	
	03. Public health emergency	1. Yes ☐	0. No ☐	
	04. Human resource management	1. Yes ☐	0. No ☐	
	05. Pharmaceuticals	1. Yes ☐	0. No ☐	
	06. Finance and Resource	1. Yes ☐	0. No ☐	
	07. Health Information System	1. Yes ☐	0. No ☐	
B	Has the PMT identified and prioritized performance issues?	1. Yes ☐	0. No ☐	→FU_022 (if No)
C	Did the PMT conduct root cause analysis for the prioritized performance related issues?	1. Yes ☐	0. No ☐	
D	Has the PMT made any of the following decisions based on the discussion of the health facility's performance?			
	01. Formulation of plans	1. Yes ☐	0. No ☐	

	02. Budget preparation	1. Yes ☐	0. No ☐	
	03. Budget reallocation	1. Yes ☐	0. No ☐	
	04. Medicine supply and drug management	1. Yes ☐	0. No ☐	
	05. Human resource management (training, reallocation, etc.)	1. Yes ☐	0. No ☐	
	06. Advocacy for policy, programmatic, or strategic decisions from the higher level	1. Yes ☐	0. No ☐	
	07. Health services (preventive, promotive, clinical, rehabilitative) planning	1. Yes ☐	0. No ☐	
	08. Promotion of service quality/improvement	1. Yes ☐	0. No ☐	
	09. Reducing the gender gap in the provision of health services	1. Yes ☐	0. No ☐	
	10. Involvement of the community and local government	1. Yes ☐	0. No ☐	
E	Was an action plan developed?	1. Yes ☐	0. No ☐	
F	Has any follow-up action taken place based on the decisions made during the previous meetings on performance-related issues? (e.g., referring performance-related issues for solution to the higher	1. Yes ☐	0. No ☐	
FU_022	Are any data display tools used during the performance review meetings?	1. Yes ☐	0. No ☐	
FU_023	Which display tools were used during the performance review meetings in the review three months?	Manual/paper-based……1<br>Electronic……2<br>Both……3		
FU_024	Were the performance review meeting minutes circulated to all members?	1. Yes ☐	0. No ☐	

ANNUAL PLANNING

FU_025	Does the heath facility have a five years strategic plan of the facility?	1. Yes, observed ☐	0. No ☐	
FU_026	Does the heath facility have annual plan for the current year?	1. Yes, observed ☐	0. No ☐	➔FU_030 (if No)
FU_027	Does the health facility plan reflect use of data from the HMIS for problem identification and/or root cause analysis?	1. Yes ☐	0. No ☐	
FU_028	Does the health facility use HMIS data for target setting?	1. Yes ☐	0. No ☐	

SUPERVISION BY THE WOREDA

Supplement to: Worku AG, Midekssa WD, Tilahun HA, et al. The impact of health information system interventions on maternal and child health service utilizations in Ethiopia: quasi-experimental study. *Glob Health Sci Pract*. 2024;12(6):2400145. https://doi.org/10.9745/GHSP-D-24-00145

FU_030	How many times did the Region/Zone/Woreda supervisor visit your health facility over the review three months?(ISS and/or HMIS supervisory visit)	More than four times............1<br>Four times............2<br>Three times............3<br>Two times............4<br>One time............5<br>None............0		➔FU_035 (if No)
FU_031	Did the supervisor use the integrated supportive supervision and/or HMIS checklist during the most recent visit?	1. Yes ☐	0. No ☐	
FU_032	Did the supervisor(s) check the data quality?	1. Yes ☐	0. No ☐	
FU_033	Over the review three months, did the supervisor discuss your health facility's performance based on HMIS information?	1. Yes ☐	0. No ☐	➔FU_035 (if No)
FU_034	If FU_033 is Yes, did the supervisor help you to make a decision or take corrective action based on the discussion?	1. Yes ☐	0. No ☐	
FU_035	Did the supervisor send a report/ written feedback to the health facility on the last supervisory visit(s)?	1. Yes ☐	0. No ☐	
**SUPERVISION BY THE HEALTH CENTER**				
FU_036	Did the Health center conduct internal supportive supervision/mentorship?	1. Yes ☐	0. No ☐	➔FU_038 (if No)
FU_037	If yes, please check if there is written feedback on the last supervisory visit/mentorship?	1. Yes, observed ☐	0. No ☐	

**DATA DISSEMINATION OUTSIDE HEALTH SECTOR**				
FU_038	Did the health facility submit/present health sector performance reports to a kebele/Woreda council (or other entities outside the health sector) in the past 12 months?	1. Yes ☐	0. No ☐	➔FU_040
FU_039	Do those reports/presentations use data from the HMIS to show the health facility's progress?	1. Yes ☐	0. No ☐	
FU_040	Is there a website updated at least annually for accessing the health facility's HMIS data by the general public?	1. Yes ☐	0. No ☐	
FU_041	Are health facility performance data shared with general public via printed materials (e.g., brochures, newsletter, etc.) in the past six months?	1. Yes ☐	0. No ☐	
FU_042	Did the health facility conducted assessment(s) and disseminate findings in the last six months?	1. Yes, assessment is conducted and findings disseminated ☐<br>2. Assessment is conducted, but findings are not disseminated ☐<br>0. No assessment conducted ☐		

FU_043	Did the health facility hold performance review meeting with community representatives in the last six months?	1. Yes ☐	0. No ☐	

GPS COORDINATES				
**FQ_113**	Please capture the GPS coordinates of health office. **(Note: Enumerator may have to go outside to capture the coordinates)**			

Data use and data quality assessment tool: Health Post Level

Purpose

1. Identify CHIS data quality, disaggregated data, and information use issues.
2. Quantify the levels of data quality (accuracy, reporting timeliness, and completeness) and information use status (access to CHIS data, existence of analyzed data, and use of CHIS data for monitoring and planning).
3. Identify issues/problems with data processing and processes for information use.

Summary of Information Collected via the RHIS Performance Diagnostic Tool at the Health Post Level

Measuring Data Quality

Through analysis of program indicators, the RHIS Performance Diagnostic Tool quantifies the status of data completeness, timeliness, and accuracy, and thus provides valuable information on the adequacy of health post data to support planning and monitoring. The data quality assessment section of this tool is aligned with the data verification aspect of the DQR Tool. The RHIS Performance Diagnostic Tool has the following core recommended indicators to assess data quality:

S.N	Category	Indicators (data elements)	Remark
1	Maternal health	Number of births attended by skilled attendants	
2	Family planning	Total number of family planning acceptors	
3	Immunization	# of children under one year of age who have received third dose of pentavalent (Penta3) vaccine	

4	Disease	Number of under-five children with pneumonia	
5	Disease	Confirmed malaria cases	

At the health post level, the diagnostic tool compares the reported value of an indicator for a selected reporting period to recoded data by reviewing the source document for the same health post and period. The result is an estimate of the accuracy of reporting for the indicators in question for the whole program.

Measuring Information Use

The tool measures the continuous use of information to guide day-to-day operations, track performance, learn from past results, and improve service delivery. The tool focuses on the use of CHIS data for analytic report production, discussion, decision/actions, target setting, planning, and monitoring.

Assessing CHIS Data Management Processes

This section assesses various aspects of CHIS data management processes, including:

- Data processing, analysis, and presentation: the availability of a copy of CHIS data management guidelines; use of standardized CHIS data collection and reporting tools; evidence of data analysis; and visual representation of data.
- Data quality check: presence of data quality assurance guideline and tools; clearly assigned roles and responsibilities for data review; and regular internal data quality checks conducted by the health post.
- Feedback: existence of formal feedback loops to the staff collecting the data; health facilities receive regular written feedback on their performance; and quality of reported data.
- Supervision quality: supervision frequency; checking data quality; using data for discussion; helping in decision making; and supervisory feedback.

Data Collection Method

- Key informant interviews (health post in-charge and data manager, or those responsible for compilation, reporting, and analysis of data)
- Document review and observation (CHIS reports, electronic database, planning documents, meeting minutes, feedback reports/notes, guidelines)

Supplement to: Worku AG, Mideksa WD, Tilahun HA, et al. The impact of health information system interventions on maternal and child health service utilizations in Ethiopia: quasi-experimental study. *Glob Health Sci Pract*. 2024;12(6):2400145. https://doi.org/10.9745/GHSP-D-24-00145

Part I. Data Quality Assessment: Health Post Form

**INTERVIEWER VISITS**		
HPQ_101.	Interview date	
HPQ_102.	Interviewer name	
HPQ_103.	Interviewer code<br>Please enter your 3-character identifier.	
**HEALTH POST IDENTIFICATION**		
HPQ_104.	Select region	Addis Ababa<br>Afar<br>Amhara<br>Beneshangul Gumuz<br>Dire Dawa<br>Gambella<br>Harari<br>Oromiya<br>SNNPR<br>Somali<br>Tigray
HPQ_105.	Zone/Sub-city<br>Please enter the alphanumeric code that identifies this level (up to 11 alphanumeric digits).	
HPQ_106.	Woreda<br>Please enter the alphanumeric code that identifies this level (up to 11 alphanumeric digits).	
HPQ_107.	Health post number<br>(Please enter the unit number up to 10 digits, include leading zeros.)	
HPQ_108.	Health post name	
HPQ_109.	Urban/rural	Urban 1<br>Rural 2
**GENERAL INFORMATION**		
**READ THE FOLLOWING STATEMENT TO THE MANAGER, THE PERSON IN-CHARGE OF THE HEALTH POST, OR THE MOST SENIOR HEALTH WORKER RESPONSIBLE FOR OUTPATIENT SERVICES WHO IS PRESENT AT THE HEALTH POST.**<br>Good day! My name is ____________________. We are here on behalf of [IMPLEMENTING AGENCY] conducting a survey of health facilities to assist the government in knowing more about the performance of routine health information systems in [COUNTRY].		

Supplement to: Worku AG, Midekssa WD, Tilahun HA, et al. The impact of health information system interventions on maternal and child health service utilizations in Ethiopia: quasi-experimental study. *Glob Health Sci Pract*. 2024;12(6):2400145. https://doi.org/10.9745/GHSP-D-24-00145

Your health post was randomly selected to participate in this study. We will be asking you questions about various health services and reporting of those services. Information about your health post may be used by the MOH, organizations supporting services in your health post, and researchers for planning service improvements or for conducting further studies of health services.

Neither your name nor that of any other health worker respondents participating in this study will be included in the dataset or in any report; however, there is a small chance that any of these respondents may be identified later. We are asking for your help to ensure that the information we collect is accurate.

You may refuse to answer any question or choose to stop the interview at any time. However, we hope you will answer the questions, which will benefit the clients you serve, the nation, and yourself.

If there are questions for which someone else is the most appropriate person to provide the information, we would appreciate it if you would introduce us to that person to help us collect the information.

At this point, do you have any questions about the study? Do I have your agreement to proceed?

____

INTERVIEWER'S SIGNATURE INDICATING CONSENT OBTAINED

HPQ_110.	May I begin the interview?	1. YES ☐<br>2. NO ☐	

PLEASE SELECT THE 3 REVIEW MONTHS THAT WILL BE USED DURING THIS ASSESSMENT	
Month_1	Meskerem 2012……1<br>Tikemet 2012……2<br>Hidar 2012……3<br>Tahesas 2012……4<br>Tir 2012……5<br>Yekatit 2012……6<br>Megabit 2012……7<br>Miyazia 2012……8<br>Ginbot 2012……9<br>Sene 2012……10<br>Hamle 2012……11<br>Nehase 2012……12
Month_2	Meskerem 2012……1<br>Tikemet 2012……2<br>Hidar 2012……3<br>Tahesas 2012……4<br>Tir 2012……5<br>Yekatit 2012……6<br>Megabit 2012……7<br>Miyazia 2012……8<br>Ginbot 2012……9<br>Sene 2012……10<br>Hamle 2012……11<br>Nehase 2012……12
Month_3	Meskerem 2012……1<br>Tikemet 2012……2<br>Hidar 2012……3

Supplement to: Worku AG, Mideksa WD, Tilahun HA, et al. The impact of health information system interventions on maternal and child health service utilizations in Ethiopia: quasi-experimental study. *Glob Health Sci Pract*. 2024;12(6):2400145. https://doi.org/10.9745/GHSP-D-24-00145

	Tahesas 2012……………………………………4<br>Tir 2012…………………………………………5<br>Yekatit 2012……………………………………6<br>Megabit 2012……………………………………7<br>Miyazia 2012……………………………………8<br>Ginbot 2012……………………………………9<br>Sene 2012………………………………………10<br>Hamle 2012……………………………………11<br>Nehase 2012……………………………………12

RESOURCES FOR DATA ASSESSMENT			
HPQ_10.	Are all the HEWs or health post's staff responsible to enter data/compile the health post reports?	1. Yes ☐	0. No ☐
HPQ_11.	Are all the HEWs/health post staff involved in reviewing the quality of compiled data prior to submission to the next level, e.g., to health center or Woreda/District health office?	Yes 1<br>Partly (the data are reviewed but not all HEWs/staff are involved)2<br>Not at all0	
HPQ_12.	Are the HEWs/health post staffs trained in:		
	C. CHIS related data entry/compilation	Yes (all received training in the past 12 months)1<br>Mostly (all received training but not in the past 12 months)2<br>Partly (some staff have received training in the past 12 months 3<br>Not at all0	
	D. CHIS related data review and quality control?	Yes (staff have received training in the past 12 months) 1<br>Mostly (all staff have received training but not in the past 12 months)2<br>Partly (some staff have received training in the past 12 months)3<br>Not at all0	

INDICATOR DEFINITIONS AND REPORTING GUIDELINES				
HPQ_13.	Does the health post have HMIS indicator definitions guideline?	1. Yes, observed ☐	2. No ☐	
HPQ_14.	Is the CHIS user's guideline available at the health post?	1. Yes, observed ☐	2. No ☐	

1. IMMUNIZATION INDICATOR

PENTAVALENT/DTP THIRD DOSE (PENTA/DTP) IN CHILDREN UNDER 1 YEAR

Supplement to: Worku AG, Midekssa WD, Tilahun HA, et al. The impact of health information system interventions on maternal and child health service utilizations in Ethiopia: quasi-experimental study. *Glob Health Sci Pract*. 2024;12(6):2400145. https://doi.org/10.9745/GHSP-D-24-00145

HPQ_15.	Does this health post provide immunization services?	1. Yes ☐	0. No ☐	➔HPQ_026 (if No)
SOURCE DOCUMENTS AND REPORTS				
HPQ_16.	Does this health post report immunization data to a reporting system?	1. Yes ☐	0. No ☐	➔HPQ_026 (if No)
HPQ_17.	To which of the following reporting systems does the health post report immunization data?<br>(Please check the reporting forms used by the health post)			
**A**	Community Health Information System (CHIS)	1. Yes ☐	0. No ☐	
**B**	EPI program	1. Yes ☐	0. No ☐	
**C**	Nongovernmental organizations or institutions	1. Yes ☐	0. No ☐	
**D**	Other reporting system	1. Yes ☐____________<br>SPECIFY	0. No ☐	
HPQ_18.	What is the source document used by this health post for monthly reporting of Penta3? We are primarily interested in the source document that is used for compiling monthly summary statistics for Penta3. Please report if any locally developed documents are used.	Service tally sheets 1<br>Field book2<br>EPI register3<br>Integrated MCH card 4<br>Other (specify) ____________________96		

REVIEW THE SOURCE DOCUMENTS USED TO COMPILE AND SUMMARIZE INFORMATION FOR MONTHLY REPORTING FOR PENTA3 AND ANSWER THE FOLLOWING QUESTIONS

HPQ_19.	Please confirm the availability of **source documents** for Penta3for Month 1 to Month 3. If available, please **Recount** the number of Penta immunizations recorded in the **source document** for Month 1 to Month 3.	**(A) Source documents available**				**(B) Recount the number of Penta3 immunizations in the source documents (if none, please enter 0)**
		**Yes, available and complete***	**Yes, available but partly** complete**	**Yes, available but no data recorded**	**No**	
**01**	**Month 1**	1	2	3	0	☐☐☐☐
**02**	**Month 2**	1	2	3	0	☐☐☐☐
**03**	**Month 3**	1	2	3	0	☐☐☐☐

Supplement to: Worku AG, Midekssa WD, Tilahun HA, et al. The impact of health information system interventions on maternal and child health service utilizations in Ethiopia: quasi-experimental study. *Glob Health Sci Pract*. 2024;12(6):2400145. https://doi.org/10.9745/GHSP-D-24-00145

Take the last 15 entries recorded in the register for each reporting period and check if all the data elements relevant to the selected indicator are filled in.If the service tally sheet is the source document, please takeout the family folder for the last 15 entries recorded in the service tally sheet for each reporting period and check if all the data elements relevant for the selected indicator are filled in.

*COMPLETE means that the source document contains the data relevant to the selected indicator.

**PARTLY: the register is available but some information is missing.

HPQ_20.	If the source document (EPI register/service tally sheet/field book) is not available, what are the possible reasons?	Storage or archiving problems 1<br>Absence of designated staff2<br>Stock out of source document3<br>Other (specify): ____________________96
HPQ_21.	If the source document is partially complete or has no data, what are the possible reasons for the missing data?	Staffing issue(s)(shortage, absence)1<br>Not understanding the data element 2<br>Presence of other vertical reporting requirement 3<br>Data burden (too much data elements to be recorded)……………………………………………………………………..4<br>The recording tool is not designed as user friendly 5<br>Other (specify): ____________________96

REVIEW THE MONTHLY REPORTS FOR DPT3 (PENTA3) AND ANSWER THE FOLLOWING QUESTIONS.

HPQ_22.	Please confirm the availability of **monthly reports** for month 1 to month 3. If available, please **report** the number of Penta3 immunizations recorded in the **Service monthly reports** for month 1 to month 3.	**(A) Monthly reports available**				**(B) Record the number of Penta3 immunizations from the monthly reports (if missing, leave blank)**
		**Yes, available and complete***	**Yes, available but partly** complete**	**Yes, available but no data recorded**	**No**	
01	**Month 1**	1		3	0	
02	**Month 2**	1		3	0	
03	**Month 3**	1		3	0	

*COMPLETE means that the monthly report contains the data relevant to the selected indicator.

**PARTLY: the monthly report is available but some information is missing.

DATA COMPLETENESS

HPQ_23.	If the monthly report of EPI is not available, what are the possible reasons?	Storage or archiving problems 1<br>Absence of designated staff2<br>Stock out of reporting forms3<br>Other (specify): ____________________96

Supplement to: Worku AG, Mideksa WD, Tilahun HA, et al. The impact of health information system interventions on maternal and child health service utilizations in Ethiopia: quasi-experimental study. *Glob Health Sci Pract*. 2024;12(6):2400145. https://doi.org/10.9745/GHSP-D-24-00145

HPQ_24.	If the monthly report of EPI is partially complete or has no data, what are the possible reasons for the missing data?	Staffing issue(s)(shortage, absence)1<br>Not understanding the data element 2<br>Presence of other vertical reporting requirement 3<br>Data burden (too much data elements to be recorded)…………………………………………………………………………4<br>The recording tool is not designed as user friendly 5<br>There is no client to be reported 6<br>Other (specify): ______________________96
DISCREPANCIES		
HPQ_25.	If there was a discrepancy observed between the **source document** and the **monthly report**, what are the reasons for the discrepancy?	Data entry errors1<br>Arithmetic errors2<br>Information from all source documents not compiled correctly ……………………………………………………………….3<br>Data burden (too much data elements to be reported)…………………………………………………………………4<br>Illegible writing on the source document (not readable) ……………………………………………………..……..5<br>Lack of emphasis for data accuracy…………………6<br>Other (specify) ________________96

**2. FAMILY PLANNING INDICATOR**				
TOTAL NUMBER OF WOMEN RECEIVED MODERN CONTRACEPTIVE METHODS				
HPQ_26.	Does this health post provide Family planning services?	1. Yes ☐	0. No ☐	➔HPQ_037 (if No)
SOURCE DOCUMENTS AND REPORTS				
HPQ_27.	Does this health post report family planning data to a reporting system?	1. Yes ☐	0. No ☐	➔HPQ_037 (if No)
HPQ_28.	To which of the following reporting systems does the health post report family planning data?<br>(Please check the reporting forms used by the health post)			
**A**	Community Health Information System (CHIS)	1. Yes ☐	0. No ☐	
**B**	family planning department/program	1. Yes ☐	0. No ☐	
**C**	Nongovernmental organizations or institutions	1. Yes ☐	0. No ☐	
**D**	Other reporting system	1. Yes ☐__________<br>SPECIFY	0. No ☐	

Supplement to: Worku AG, Midekssa WD, Tilahun HA, et al. The impact of health information system interventions on maternal and child health service utilizations in Ethiopia: quasi-experimental study. *Glob Health Sci Pract*. 2024;12(6):2400145. https://doi.org/10.9745/GHSP-D-24-00145

HPQ_29.	What is the source document used by this health post for monthly reporting of family planning? We are primarily interested in the source document that is used for compiling monthly summary statistics for family planning. Please report if any locally developed documents are used.	Service tally sheets 1<br>Field book2<br>Family planning register3<br>Female health card 4<br>Other (specify) ________________________96

REVIEW THE SOURCE DOCUMENTS USED TO COMPILE AND SUMMARIZE INFORMATION FOR MONTHLY REPORTING FOR FAMILY PLANNING AND ANSWER THE FOLLOWING QUESTIONS

HPQ_30.	Please confirm the availability of **source documents** for family planning users for month 1 to month 3. If available, please **Recount** the number of family planning users recorded in the **source document** for month 1 to month 3	**(A) Source documents available**				**(B) Recount the number of family planning usersin the source documents (if none, please enter 0)**
		**Yes, available and complete***	**Yes, available but partly** complete**	**Yes, available but no data recorded**	**No**	
**01**	**Month 1**	1	2	3	0	☐☐☐☐
**02**	**Month 2**	1	2	3	0	☐☐☐☐
**03**	**Month 3**	1	2	3	0	☐☐☐☐

the last 15 entries recorded in the FP register for each reporting period and check if all the data elements relevant to the selected indicator are filled in.If the service tally sheet is the source document, please takeout the family folder for the last 15 entries recorded in the service tally sheet for each reporting period and check if all the data elements relevant for the selected indicator are filled in.

*COMPLETE means that the source document contains the data relevant to the selected indicator. Take

**PARTLY: the register is available but some information is missing.

HPQ_31.	If the source document (family planning register/service tally sheet/field book) is not available, what are the possible reasons?	Storage or archiving problems 1<br>Absence of designated staff2<br>Stock out of source document3<br>Other (specify): ________________________96
HPQ_32.	If the source document (family planning register/service tally sheet/field book)is partially complete or has no data, what are the possible reasons for the missing data?	Staffing issue(s)(shortage, absence)1<br>Not understanding the data element 2<br>Presence of other vertical reporting requirement 3<br>Data burden (too much data elements to be recorded)……………………………………………………………………….4<br>The recording tool is not designed as user friendly 5<br>Other (specify): ________________________96

REVIEW THE MONTHLY REPORTS FOR TOTAL NUMBER OF FAMILY PLANNING ACCEPTORSAND ANSWER THE FOLLOWING QUESTIONS.

Supplement to: Worku AG, Midekssa WD, Tilahun HA, et al. The impact of health information system interventions on maternal and child health service utilizations in Ethiopia: quasi-experimental study. *Glob Health Sci Pract*. 2024;12(6):2400145. https://doi.org/10.9745/GHSP-D-24-00145

HPQ_33.	Please confirm the availability of **monthly reports** for month 1 to month 3. If available, please **report** the number of family planning users recorded in the **monthly reports** for month 1 to month 3	**(A) Monthly reports available**				**(B) Record the number of FP acceptor from the monthly reports (if missing, leave blank)**
		**Yes, available and complete***	**Yes, available but partly** complete**	**Yes, available but no data recorded**	**No**	
**01**	**Month 2**	1	2	3	0	☐☐☐☐
**02**	**Month 3**	1	2	3	0	☐☐☐☐
**03**	**Month 2**	1	2	3	0	☐☐☐☐

*COMPLETE means that the monthly report contains the data relevant to the selected indicator.

**PARTLY: the monthly report is available but some information is missing.

DATA COMPLETENESS

HPQ_34.	If the monthly report of FP is not available, what are the possible reasons?	Storage or archiving problems 1<br>Absence of designated staff2<br>Stock out of source document3<br>Other (specify): ________________________________96
HPQ_35.	If the monthly report of FP is partially complete or has no data, what are the possible reasons for the missing data?	Staffing issue(s)(shortage, absence)1<br>Not understanding the data element 2<br>Presence of other vertical reporting requirement 3<br>Data burden (too much data elements to be recorded)…………………………………………………………………………4<br>The recording tool is not designed as user friendly 5<br>There is no client to be reported 6<br>Other (specify): ________________________________96

DISCREPANCIES

HPQ_36.	If there was a discrepancy observed between the **source document** and the **monthly report**, what are the reasons for the discrepancy?	Data entry errors1<br>Arithmetic errors2<br>Information from all source documents not compiled correctly …………………………………………………………………3<br>Data burden (too much data elements to be reported)……………………………………………………………………4<br>Illegible writing on the source document (not readable) ……………………………………………………………..……..5<br>Lack of emphasis for data accuracy…………………6<br>Other (specify) ________________________96

Supplement to: Worku AG, Midekssa WD, Tilahun HA, et al. The impact of health information system interventions on maternal and child health service utilizations in Ethiopia: quasi-experimental study. *Glob Health Sci Pract*. 2024;12(6):2400145. https://doi.org/10.9745/GHSP-D-24-00145

3. MALARIA INDICATOR

**NUMBER OF CONFIRMED MALARIA CASES**				
HPQ_38.	Does this health post diagnose and treat malaria?	1. Yes ☐	0. No ☐	➔HPQ_048 (if No)
**SOURCE DOCUMENTS AND REPORTS**				
HPQ_39.	Does this health post report malaria data to a reporting system?	1. Yes ☐	0. No ☐	➔HPQ_048 (if No)
HPQ_40.	To which of the following reporting systems does the health post report malaria data?			
**A**	Community Health Information System (CHIS)	1. Yes ☐	0. No ☐	
**B**	Malaria program	1. Yes ☐	0. No ☐	
**C**	Nongovernmental organizations or institutions	1. Yes ☐	0. No ☐	
**D**	Other reporting system	1. Yes ☐ ____________ SPECIFY	0. No ☐	
HPQ_41.	What is the source document used by this health post for monthly reporting of malaria? We are primarily interested in the source document that is used for compiling monthly summary statistics for malaria. Please report if any locally developed documents are used.	Service tally sheet1<br>Field book2<br>Disease information tally sheet3<br>Curative care service register4<br>ICCM register5<br>Health card6<br>Other (specify) ____________96		

REVIEW THE SOURCE DOCUMENTS USED TO COMPILE AND SUMMARIZE INFORMATION FOR MONTHLY REPORTING FOR MALARIA AND ANSWER THE FOLLOWING QUESTIONS

HPQ_42.	Please confirm the availability of **source documents** for malaria month 1 to month 3. If available, please **Recount** the number of conf. malaria cases recorded in the **source document** for month 1 to month 3.	**(A) Source documents available**				**(B) Recount the number of conf. malaria casesin the source documents (if none, please enter 0)**
		**Yes, available and complete***	**Yes, available but partly** complete**	**Yes, available but no data recorded**	**No**	
**01**	**Month 1**	1	2	3	0	☐☐☐☐
**02**	**Month 2**	1	2	3	0	☐☐☐☐
**03**	**Month 3**	1	2	3	0	☐☐☐☐

Supplement to: Worku AG, Midekssa WD, Tilahun HA, et al. The impact of health information system interventions on maternal and child health service utilizations in Ethiopia: quasi-experimental study. *Glob Health Sci Pract*. 2024;12(6):2400145. https://doi.org/10.9745/GHSP-D-24-00145

	Take the last 15 entries recorded in the OPD register or disease tally sheet for each reporting period and check if all the data elements relevant to the selected indicator are filled in.If the service tally sheet or disease information tally sheet is the source document, please takeout the family folder for the last 15 entries recorded in the service tally sheet for each reporting period and check if all the data elements relevant for the selected indicator are filled in.<br>*COMPLETE means that the source document contains the data relevant to the selected indicator.<br>**PARTLY: the register is available but some information is missing.	
HPQ_43.	If the source document (OPD register/ disease tally sheet) is not available, what are the possible reasons?	Storage or archiving problems 1<br>Absence of designated staff2<br>Stock out of source document3<br>Other (specify): ________________________________96
HPQ_44.	If the source documents (OPD register/ OPD tally sheet) are partially complete or have no data, what are the possible reasons for the missing data?	Staffing issue(s)(shortage, absence)1<br>Not understanding the data element 2<br>Presence of other vertical reporting requirement 3<br>Data burden (too much data elements to be recorded)4<br>The recording tool is not designed as user friendly 5<br>Other (specify): ________________________________96

REVIEW THE MONTHLY REPORTS FOR CONFIRMED MALARIA AND ANSWER THE FOLLOWING QUESTIONS.

HPQ_45.	Please confirm the availability of **monthly reports** for month 1 to month 3. If available, please **report** the number of # of conf. malaria cases recorded in the **monthly reports** for month 1 to month 3.	**(A) Monthly reports available**				**(B) Record the number of # of conf. malaria cases from the monthly reports (if missing, leave blank)**
		**Yes, available and complete***	**Yes, available but partly** complete**	**Yes, available but no data recorded**	**No**	
**01**	**Month 2**	1	2	3	0	☐☐☐☐
**02**	**Month 3**	1	2	3	0	☐☐☐☐
**03**	**Month 2**	1	2	3	0	☐☐☐☐

*COMPLETE means that the monthly report contains the data relevant to the selected indicator.

**PARTLY: the monthly report is available but some information is missing.

DATA COMPLETENESS

HPQ_46.	If the monthly report for malaria is not available, what are the possible reasons?	Storage or archiving problems 1<br>Absence of designated staff2<br>Stock out of source document3<br>Other (specify): ________________________________96

Supplement to: Worku AG, Midekssa WD, Tilahun HA, et al. The impact of health information system interventions on maternal and child health service utilizations in Ethiopia: quasi-experimental study. *Glob Health Sci Pract*. 2024;12(6):2400145. https://doi.org/10.9745/GHSP-D-24-00145

HPQ_47.	If the monthly report of malaria is partially complete or has no data, what are the possible reasons for the missing data?	Staffing issue(s)(shortage, absence)1<br>Not understanding the data element 2<br>Presence of other vertical reporting requirement 3<br>Data burden (too much data elements to be recorded)…………………………………………………………………………4<br>The recording tool is not designed as user friendly 5<br>There is no client to be reported 6<br>Other (specify): ________________________________96
DISCREPANCIES		
HPQ_48.	If there was a discrepancy observed between the **source document** and the **monthly report**, what are the reasons for the discrepancy?	Data entry errors1<br>Arithmetic errors2<br>Information from all source documents not compiled correctly ………………………………………………………………….3<br>Data burden (too much data elements to be reported)…………………………………………………………………4<br>Illegible writing on the source document (not readable) ……………………………………………………..……..5<br>Lack of emphasis for data accuracy…………………6<br>Other (specify) ________________________96

**4. DISEASE IN UNDER FIVE CHILDREN INDICATOR**				
NUMBER OF UNDER FIVE CHILDREN WITH PNEUMONIA				
HPQ_49.	Does this health post provide ICCM (pneumonia treatment) services?	1. Yes ☐	0. No ☐	➔HPQ_059(if No)
SOURCE DOCUMENTS AND REPORTS				
HPQ_50.	Does this health post report pneumonia data to a reporting system?	1. Yes ☐	0. No ☐	➔HPQ_059 (if No)
HPQ_51.	To which of the following reporting systems does the health post report pneumonia data?			
**A**	Community Health Information System (CHIS)	1. Yes ☐	0. No ☐	
**B**	IMNCI program	1. Yes ☐	0. No ☐	
**C**	Nongovernmental organizations or institutions	1. Yes ☐	0. No ☐	
**D**	Other reporting system	1. Yes ☐___________<br>SPECIFY	0. No ☐	

Supplement to: Worku AG, Mideksa WD, Tilahun HA, et al. The impact of health information system interventions on maternal and child health service utilizations in Ethiopia: quasi-experimental study. *Glob Health Sci Pract*. 2024;12(6):2400145. https://doi.org/10.9745/GHSP-D-24-00145

HPQ_52.	What is the source document used by this health post for monthly reporting of pneumonia? We are primarily interested in the source document that is used for compiling monthly summary statistics for malaria. Please report if any locally developed documents are used.	Service tally sheet1<br>Field book2<br>Disease information tally sheet3<br>Curative care service register4<br>ICCM register5<br>Health card6<br>Other (specify) ___________________________96

REVIEW THE SOURCE DOCUMENTS USED TO COMPILE AND SUMMARIZE INFORMATION FOR MONTHLY REPORTING FOR PNEUMONIA AND ANSWER THE FOLLOWING QUESTIONS

HPQ_53.	Please confirm the availability of **source documents** for pneumonia in under five for month 1 to month 3. If available, please **Recount** the number of under-five children with pneumonia recorded in the **source document** for month 1 to month 3.	**(A) Source documents available**				**(B) Recount the number ofunder-five children with pneumonia in the source documents (if none, please enter 0)**
		**Yes, available and complete***	**Yes, available but partly** complete**	**Yes, available but no data recorded**	**No**	
**01**	**Month 1**	1	2	3	0	☐☐☐☐
**02**	**Month 2**	1	2	3	0	☐☐☐☐
**03**	**Month 3**	1	2	3	0	☐☐☐☐

Take the last 15 entries recorded in the ICCM register for each reporting period and check if all the data elements relevant to the selected indicator are filled in.If the service tally sheet is the source document, please takeout the family folder for the last 15 entries recorded in the service tally sheet for each reporting period and check if all the data elements relevant for the selected indicator are filled in.

*COMPLETE means that the source document contains the data relevant to the selected indicator.

**PARTLY: the register is available but some information is missing.

HPQ_54.	If the source document (ICCM register/ Service tally sheet) is not available, what are the possible reasons?	Storage or archiving problems 1<br>Absence of designated staff2<br>Stock out of source document3<br>Other (specify): ______________________________96
HPQ_55.	If the source documents (ICCM register/ service tally sheet) are partly complete or have no data, what are the possible reasons for the missing data?	Staffing issue(s)(shortage, absence)1<br>Not understanding the data element 2<br>Presence of other vertical reporting requirement 3<br>Data burden (too much data elements to be recorded)……………………………………………………………………….4<br>The recording tool is not designed as user friendly 5<br>Other (specify): ______________________________96

Supplement to: Worku AG, Midekssa WD, Tilahun HA, et al. The impact of health information system interventions on maternal and child health service utilizations in Ethiopia: quasi-experimental study. *Glob Health Sci Pract*. 2024;12(6):2400145. https://doi.org/10.9745/GHSP-D-24-00145

REVIEW THE MONTHLY REPORTS FOR PNEUMONIA IN UNDER_FIVE CHILDREN AND ANSWER THE FOLLOWING QUESTIONS.						
HPQ_56.	Please confirm the availability of **monthly reports** for month 1 to month 3. If available, please **report** the number under-five children with pneumonia recorded in the **monthly reports** for month 1 to month 3.	**(A) Monthly reports available**				**(B) Record the number of under-five children with pneumonia from the monthly reports (if missing, leave blank)**
		**Yes, available and complete***	**Yes, available but partly** complete**	**Yes, available but no data recorded**	**No**	
**01**	**Month 2**	1	2	3	0	
**02**	**Month 3**	1	2	3	0	
**03**	**Month 2**	1	2	3	0	

*COMPLETE means that the monthly report contains the data relevant to the selected indicator.

**PARTLY: the monthly report is available but some information is missing.

DATA COMPLETENESS

HPQ_57.	If the monthly report for pneumonia in under-five children is not available, what are the possible reasons?	Storage or archiving problems 1<br>Absence of designated staff2<br>Stock out of source document3<br>Other (specify): ________________________________96
HPQ_58.	If the monthly report of pneumonia in under-five children is partially complete or has no data, what are the possible reasons for the missing data?	Staffing issue(s)(shortage, absence)1<br>Not understanding the data element 2<br>Presence of other vertical reporting requirement 3<br>Data burden (too much data elements to be recorded)…………………………………………………………………<br>……4<br>The recording tool is not designed as user friendly 5<br>There is no client to be reported 6<br>Other (specify): ________________________________96

DISCREPANCIES

Supplement to: Worku AG, Midekssa WD, Tilahun HA, et al. The impact of health information system interventions on maternal and child health service utilizations in Ethiopia: quasi-experimental study. *Glob Health Sci Pract*. 2024;12(6):2400145. https://doi.org/10.9745/GHSP-D-24-00145

HPQ_59.	If there was a discrepancy observed between the **source document** and the **monthly report**, what are the reasons for the discrepancy?	Data entry errors1<br>Arithmetic errors2<br>Information from all source documents not compiled correctly ……………………………………………………………3<br>Data burden (too much data elements to be reported)……………………………………………………………4<br>Illegible writing on the source document (not readable) ……………………………………………………………5<br>Lack of emphasis for data accuracy…………………6<br>Other (specify) ______________________96

REPORT TIMELINESS					
HPQ_60.	A. Is there a deadline for submission of the monthly CHIS report by the health posts?		1. Yes ☐	0. No ☐	
	B. If yes, what is the deadline (date of month)?<br>(Write the end date of deadline)		Reporting deadline:<br>----------------------------------		
HPQ_61.	Does the health post record the dates of submission of monthly CHIS reports to the Health center or Woreda/District (see documentation)?		1. Yes ☐	0. No ☐	➔HPQ_062 (if No)
	**IF AVAILABLE, REVIEW THE RECORDS AND CHECK THE DATES OF SUBMISSION FOR THE THREE REVIEW MONTHS**				
		**A. Month 1**	**B. Month 2**	**C. Month 3**	
HPQ_62.	Are the CHIS monthly reports submitted on time? **(before or on the deadline)**<br>**(Current practice is submission before 23rd of the month)**	1. Service☐<br>2. Disease☐<br>0. No ☐	1. Service☐<br>2. Disease☐<br>0. No ☐	1. Service☐<br>2. Disease☐<br>3. Quarter ☐<br>0. No ☐	

DATA QUALITY ASSESSMENT MECHANISM				
HPQ_63.	Does the health post have CHIS user manual (that explains data quality check protocols)?	1. Yes, observed ☐	0. No ☐	
HPQ_64.	Does the health post have access to data quality self-assessment tools (e.g. LQAS)?	1. Yes, observed ☐	0. No ☐	
HPQ_65.	Did the health post conduct LQAS in the review three months?	1. Yes ☐	0. No ☐	➔HPQ_068 (if No)

Supplement to: Worku AG, Mideksa WD, Tilahun HA, et al. The impact of health information system interventions on maternal and child health service utilizations in Ethiopia: quasi-experimental study. *Glob Health Sci Pract*. 2024;12(6):2400145. https://doi.org/10.9745/GHSP-D-24-00145

HPQ_066	If Yes, which types of the report are covered by the LQAS?			
	**Types of Reports**	**Month 1**	**Month 2**	**Month 3**
	03. For service report	1. Yes ☐<br>0. No ☐	1. Yes ☐<br>0. No ☐	1. Yes ☐<br>0. No ☐
	04. For disease report	1. Yes ☐<br>0. No ☐	1. Yes ☐<br>0. No ☐	1. Yes ☐<br>0. No ☐
HPQ_67.	Does the health post maintain a record of LQAS check sheets conducted in the review three months?	1. Yes, observed ☐	0. No ☐	
HPQ_68.	Does the health post maintain records of feedback to staff on data quality self-assessment findings?	1. Yes, observed ☐	0. No ☐	

DATA PROCESSING AND ANALYSIS

HPQ_69.	Does the health post use an electronic database/system to enter and analyze routine health data?	1. Yes ☐	0. No ☐	➔HPQ_069(if No)
HPQ_70.	If yes, indicate the type of electronic system used for routine data entry and analysis.			

Electronic system	C. For data entry		D. For data analysis	
	2. Yes	0. No	1. Yes	0. No
02. National open-source data processing system (e.g., DHIS 2, eCHIS)				
02 National proprietary software(e.g., locally developed mHealth solutions)				
06. Excel-based spreadsheet				
07. Access-based data processing module				
08. Other (specify)				

HPQ_71.	**ASK RELEVANT STAFF IN THE HEALTH POST OFFICE TO SHOW UP-TO-DATE (I.E., NOT MORE THAN ONE YEAR OLD) REPORTS, DOCUMENTS, AND/OR DISPLAYS THAT CONTAIN THE FOLLOWING. THE ASSESSOR SHOULD RECORD THE OBSERVATIONS ACCORDINGLY.**		
H.	Aggregated/summary CHIS report within the review three months.	1. Yes, observed ☐	0. No ☐
I.	Demographic data on the catchment population of the health post for calculating coverage.	1. Yes, observed ☐	0. No ☐
J.	Indicators (e.g., Penta3 coverage) calculated for the health post catchment area within the review three months.	1. Yes, observed ☐	0. No ☐

Supplement to: Worku AG, Mideksa WD, Tilahun HA, et al. The impact of health information system interventions on maternal and child health service utilizations in Ethiopia: quasi-experimental study. *Glob Health Sci Pract*. 2024;12(6):2400145. https://doi.org/10.9745/GHSP-D-24-00145

K.	Comparisons between health post and health center/Woreda/District targets.	1. Yes, observed ☐	0. No ☐
L.	Comparisons of data over time, i.e., monitoring trends (e.g., for ANC, Penta3).	1. Yes, observed ☐	0. No ☐
M.	Comparisons of sex-disaggregated data (e.g., OPD visit).	1. Yes, observed ☐	0. No ☐
N.	Comparisons of service coverage between associated services (e.g. Penta1 vs OPV1, Penta3 vs OPV3).	1. Yes, observed ☐	0. No ☐

Supplement to: Worku AG, Midekssa WD, Tilahun HA, et al. The impact of health information system interventions on maternal and child health service utilizations in Ethiopia: quasi-experimental study. *Glob Health Sci Pract*. 2024;12(6):2400145. https://doi.org/10.9745/GHSP-D-24-00145

Part 2. Use of Information: Health Post Assessment Form

DATA VISUALIZATION					
HPU_1.	Does the health post prepare data visuals (graphs, tables, maps, etc.) showing achievements toward targets (indicators, geographic and/or temporal trends, and situation data)?	Yes, paper or electronic copies of data visuals observed at the health post……………………………………1<br>Yes, some paper or electronic copies of data visuals observed at the health post…………………2<br>No……………………………………0			➔HPU_005 *(if No)*
HPU_2.	If yes, what type of information is captured in the data visuals and is it updated as per the standard?				
A	Map of catchment area	1. Yes, observed ☐	2. Yes, but not updated ☐	0. No ☐	
B	Catchment population profile	1. Yes, observed ☐	2. Yes, but not updated ☐	0. No ☐	
C	Staffing	1. Yes, observed ☐	2. Yes, but not updated ☐	0. No ☐	
D	ANC coverage	1. Yes, observed ☐	2. Yes, but not updated ☐	0. No ☐	
E	Penta-3 immunization coverage	1. Yes, observed ☐	2. Yes, but not updated ☐	0. No ☐	
F	Measles immunization coverage	1. Yes, observed ☐	2. Yes, but not updated ☐	0. No ☐	
G	Malaria, all ages	1. Yes, observed ☐	2. Yes, but not updated ☐	0. No ☐	
H	Pneumonia amongst Under five	1. Yes, observed ☐	2. Yes, but not updated ☐	0. No ☐	

CHIS ANALYTIC REPORT PRODUCTION			
HPU_3.	Does the health post have analyzed CHIS data (e.g., summary tables, charts, maps)?	Yes, observed paper-based………1<br>No………………………………………0	
HPU_4.	Does the health post produce any report (annual, quarterly, etc.) based on analysis of CHIS data?<br>**(Excluding the routing monthly, quarterly, and annual summary/aggregate reports submitted to the higher level.)**	Yes, observed……………………………1<br>No ……………………………………0	➔HPU_007(if No)
HPU_5.	If yes, list the reports, indicating the frequency of the reports and the number of times the reports were actually issued in the last 12 months		

	Title of the report/bulletin **(A)**	Number of times this report is supposed to be issued per year **(B)**	Number of times this report was actually issued in the last 12 months **(C)**	Target audience of the report (e.g., FMOH, RHB, ZHD, Woreda/District health office, civil administration, parliament, HAD, community forums, general population) **(D)**
01				
02				
03				
HPU_6.	Do any of these reports contain discussions and decisions/recommendations based on key performance targets based on CHIS data, such as:			
	08. Maternal and Child health		1. Yes ☐	0. No ☐
	09. Health Promotion and Disease prevention		1. Yes ☐	0. No ☐
	10. Public health emergency		1. Yes ☐	0. No ☐
	11. Human resource management		1. Yes ☐	0. No ☐
	12. Pharmaceuticals		1. Yes ☐	0. No ☐
	13. Finance and Resource		1. Yes ☐	0. No ☐
	14. Health Information System		1. Yes ☐	0. No ☐

FEEDBACK TO HEALTH POSTS				
HPU_7.	Did the health post receive feedback reports from the health center/Woreda/District health office based on CHIS information in the review three months? ***(OBSERVE THE REPORT AND TICK ACCORDINGLY.)***	1. Yes, observed ☐	0. No ☐	➔HPU_009 (if No)
HPU_8.	If yes, indicate the types of feedback reports:			
A	Feedback on data quality (including data accuracy, reporting timeliness, and/or report completeness)	1. Yes, observed ☐	0. No ☐	
B	Feedback on service performance based on reported CHIS data (e.g., appreciation/ acknowledgement of good performance; gaps identified; resource allocation/mobilization)	1. Yes, observed ☐	0. No ☐	

ROUTINE DECISION-MAKING FORUMS AND PROCESSES AT HEALTH POST								
HPU_9.	Does the health post conduct performance monitoring team (PMT) meeting?	1. Yes ☐		0. No ☐				➔HPU_016 (if No)
HPU_10.	Are all the health post staffs participating in the performance review?	1. Yes ☐		0. No ☐				
HPU_11.	How often are the performance monitoring meetings supposed to take place?	Weekly ....1<br>Monthly .... 2<br>Quarterly ....3<br>Biannually ....4<br>Annually....5<br>No schedule.... 0						
HPU_12.	How many times did the performance monitoring meetings take place during the review three months?	More than four times1<br>Four times2<br>Three times3<br>Two times4<br>One time5<br>None 0						
HPU_13.	Were minutes of performance monitoring meetings maintained for the review three months?	Month 1		Month 2		Month 3		➔HPU_016 (if No to all)
		1. Yes ☐	0. No☐	1. Yes ☐	0. No☐	1. Yes ☐	0. No☐	
HPU_14.	Please check the performance monitoring meeting minute/records for the review months and see if the following topics were discussed.							
A	Did they have any discussions on CHIS management, such as data quality, completeness, or timeliness of reporting?	1. Yes ☐		0. No ☐				➔HPU_015 (if No)
B	Were CHIS related issues identified and prioritized?	1. Yes ☐		0. No ☐				➔HPU_015(if No)
C	Were root cause analysis conducted for the prioritized CHIS related issues?	1. Yes ☐		0. No ☐				
D	Was an action plan developed to address the CHIS related issues?							
E	Has any follow-up action taken place on the decisions made during the previous meetings on CHIS-related issues? (e.g., referring CHIS-related issues/problems for solution to the higher level)	1. Yes ☐		0. No ☐				
HPU_15. A<br><br>A	Were discussions held to review the following key performance targets (e.g., tracking progress against targets/plan):<br><br>(Please check the performance monitoring team minute/records for the review months and see if the following topics were discussed)							➔HPU_016 (if all are No)
	08. Maternal and Child health	1. Yes ☐		0. No ☐				
	09. Health Promotion and Disease prevention	1. Yes ☐		0. No ☐				
	10. Public health emergency	1. Yes ☐		0. No ☐				

	11. Human resource management	1. Yes ☐	0. No ☐	
	12. Pharmaceuticals	1. Yes ☐	0. No ☐	
	13. Finance and Resource	1. Yes ☐	0. No ☐	
	14. Health Information System	1. Yes ☐	0. No ☐	
B	Has the PMT identified and prioritized performance issues?	1. Yes ☐	0. No ☐	→HPU_016 (if No)
C	Did the PMT conduct root cause analysis for the prioritized performance related issues?	1. Yes ☐	0. No ☐	
D	Have they made any decisions based on the discussion of the health post's performance? Such as:			
	01. Formulation of plans	1. Yes ☐	0. No ☐	
	02. Budget preparation	1. Yes ☐	0. No ☐	
	03. Medicine supply and drug management	1. Yes ☐	0. No ☐	
	04. Advocacy for the community and HAD groups	1. Yes ☐	0. No ☐	
	05. Promotion of service quality/improvement	1. Yes ☐	0. No ☐	
	06. Reducing the gender gap in the provision of health services	1. Yes ☐	0. No ☐	
	07. No action required at this time	1. Yes ☐	0. No ☐	
E	Was an action plan developed?	1. Yes ☐	0. No ☐	
F	Has any follow-up action taken place based on the decisions made during the previous meetings on performance-related issues? (e.g., referring performance related issues for solution to the higher level)	1. Yes ☐	0. No ☐	

ANNUAL PLANNING

HPU_16.	Does the heath post have annual plan for the current year?	1. Yes, observed ☐	0. No ☐	
HPU_17.	Does the health post use CHIS for annual planning?	1. Yes ☐	0. No ☐	→HPU_022 (if No)
HPU_18.	Does the health post plan reflect use of data from the CHIS for target setting?	1. Yes ☐	0. No ☐	

SUPERVISION BY THE HEALTH CENTER/WOREDA/DISTRICT

Supplement to: Worku AG, Midekssa WD, Tilahun HA, et al. The impact of health information system interventions on maternal and child health service utilizations in Ethiopia: quasi-experimental study. *Glob Health Sci Pract*. 2024;12(6):2400145. https://doi.org/10.9745/GHSP-D-24-00145

HPU_20.	How many times did the health center/Woreda/District health office visit your health post over the review three months?	More than four times……………..1<br>Four times…………………………2<br>Three times………………………..3<br>Two times…………………………..4<br>One time……………………………5<br>None……………………………….0		➔HPU_028 (if No)
HPU_21.	Did the supervisor use the integrated supportive supervision and/or CHIS checklist during the most recent visit?	1. Yes ☐	0. No ☐	
HPU_22.	Did the supervisor (s) check the data quality?	1. Yes ☐	0. No ☐	
HPU_23.	During the most recent visit, did the health center/ Woreda/District health office supervisor discuss your health post's performance based on CHIS information?	1. Yes ☐	0. No ☐	➔HPU_025 (if No)
HPU_24.	If HPU_023 is Yes, did the supervisor help you to make a decision or take corrective action based on the discussion?	1. Yes ☐	0. No ☐	
HPU_25.	Did the supervisor send a report/ written feedback on the last supervisory visit(s)?	1. Yes ☐	0. No ☐	
HPU_26.	Did the health center/ Woreda/District health office conduct CHIS mentorship?	1. Yes ☐	0. No ☐	➔HPU_028 (if No)
HPU_27.	If yes, please check if there is written feedback on the last CHIS mentorship?	1. Yes, observed ☐	0. No ☐	

DATA DISSEMINATION OUTSIDE HEALTH SECTOR				
HPU_28.	Did the health post submit/present health sector performance reports to the kebele administration in the past 12 months?	1. Yes ☐	0. No ☐	➔HPU_030
HPU_29.	Do those reports/presentations use data from the CHIS to show the health post's progress?	1. Yes ☐	0. No ☐	
HPU_30.	Are health post performance data shared with the community via local publication, reports, etc.	1. Yes ☐	0. No ☐	
HPU_31.	Did the health post hold performance review meeting with community representatives in the past six month (e.g. with the kebele council, HAD leaders, etc)?	1. Yes ☐	0. No ☐	

GPS COORDINATES

Supplement to: Worku AG, Midekssa WD, Tilahun HA, et al. The impact of health information system interventions on maternal and child health service utilizations in Ethiopia: quasi-experimental study. *Glob Health Sci Pract*. 2024;12(6):2400145. https://doi.org/10.9745/GHSP-D-24-00145

**HPQ_111**	Please capture the GPS coordinates of health office. **(Note: Enumerator may have to go outside to capture the coordinates)**			

Organizational and Behavioral Assessment Tool (OBAT) – For Woreda and Higher Levels

Purpose

1. Assess whether the organizational mechanisms are in place for producing the desired results in RHIS performance.
2. Explore the extent to which a culture of information exists in the organization.
3. Identify the commitment and support of upper management for enhancing an information system.
4. Quantify the health workers' motivation, knowledge, and skills to perform RHIS tasks.

Summary of Information Collected via the OBAT

1. Promotion of information culture
   - Emphasis on data quality
   - Use of RHIS information (for planning, day-to-day operations, and monitoring)
   - Problem solving, feedback
   - Sense of responsibility
   - Empowerment/accountability

2. Individuals behavior
   - Perception of self-competency to perform RHIS tasks
   - Knowledge of RHIS (including rationale for data collection and how to perform data quality checks)
   - Skills to perform RHIS tasks (such as identification and problem solving, visually presenting data, interpretation, and evidence-based decision-making)
   - Motivation

Supplement to: Worku AG, Midekssa WD, Tilahun HA, et al. The impact of health information system interventions on maternal and child health service utilizations in Ethiopia: quasi-experimental study. *Glob Health Sci Pract*. 2024;12(6):2400145. https://doi.org/10.9745/GHSP-D-24-00145

Data Collection Methods

Paper and pencil-based self-assessment to be administered by:

- Woreda (Head /deputy head, HMIS focal person/M& officer, 1 health program supervisor or focal persons/case team coordinator)
- Zone and region (Plan and M&E Director/core process owner, HMIS focal person, 1 program case team coordinator)

The OBAT has the following parts:

- Survey relevant for staff and management at all levels
- Cadre and level-specific competency surveys

Number	Question	Result
**SURVEY FACILITATOR**		
**OBAT_101**	Survey date	
**OBAT_102**	Interviewer name	
**OBAT_103**	Interviewer code<br>Please enter your 2-character identifier.	
**OBAT_104**	Type of facility<br>[Country specific]	1 = Referral/Specialized hospital<br>2 = General hospital<br>3 = Primary hospital<br>4 = Health center<br>5 = Medium clinics<br>6 = Health post<br>7 = Woreda health office<br>8 = ZHD<br>9= RHB
**UNIT IDENTIFICATION<br>[Valid when facility type is 7, 8 or 9 – see customization notes]**		
**OBAT_105h**	Select Region	Addis Ababa<br>Afar<br>Amhara<br>Beneshangul Gumuz<br>Dire Dawa<br>Gambella<br>Harari<br>Oromiya<br>SNNPR<br>Somali<br>Tigray
**OBAT_106h**	Zone/Sub-city<br>Please enter the alphanumeric code that identifies this level (up to 11 alphanumeric digits).	
**OBAT_107h**	Woreda<br>Please enter the alphanumeric code that identifies this level (up to 11 alphanumeric digits).<br>**[Valid when type of facility is 7]**	

Supplement to: Worku AG, Midekssa WD, Tilahun HA, et al. The impact of health information system interventions on maternal and child health service utilizations in Ethiopia: quasi-experimental study. *Glob Health Sci Pract*. 2024;12(6):2400145. https://doi.org/10.9745/GHSP-D-24-00145

**OBAT_108h**	Unit name	
**OBAT_110h**	Office(s) visited<br>**(Note: it could be one or more offices from which information is collected. Please list them here.)**	______________________<br>______________________<br>______________________
**OBAT_111f**	Urban/rural	Urban ----------------------------1<br>Rural ----------------------------2
**OBAT_112f**	Managing authority	Government/public ------------------1<br>NGO/not-for-profit ------------------2<br>Private-for-profit ------------------3<br>Mission/faith-based ------------------4<br>Other (specify)<br>____________________96

Supplement to: Worku AG, Mideksa WD, Tilahun HA, et al. The impact of health information system interventions on maternal and child health service utilizations in Ethiopia: quasi-experimental study. *Glob Health Sci Pract*. 2024;12(6):2400145. https://doi.org/10.9745/GHSP-D-24-00145

Part 1. OBAT: For Staff and Management at All Levels

Introduction

This survey is part of the___________________________________(indicate name of Program/Project leading the RHIS assessment), to improve Routine Health Information Systems in the health sector. The objective of this survey is to identify strengths and weaknesses in the system with a view to developing interventions for system strengthening. Please express your opinions honestly. Your responses will remain confidential and will not be shared with anyone, except in aggregate formats. We appreciate your assistance and co-operation in completing this study.

Thank you.

OBAT_112	Survey start time<br><br>(Use the 12-hour clock system)	☐☐ : ☐☐

Number	Question	Result
SECTION 1.1: RESPONDENT BACKGROUND		
DDI	Title of the person completing the questionnaire **(circle answer)**	Regional / Zonal/woreda head/deputy head.......1<br>Regional / Zonal/woreda RHIS/M&E unit leads...... 2<br>Regional / Zonal/woreda DPC or MCH supervisors or focal persons ....3<br><br>Other (specify)______________________96
DD2	Sex	Male.....................................1<br>Female 2
DD3a	Highest level of education achieved.<br><br>**(Circle one)**	Primary/Elementary ....1<br>Secondary/High School ....2<br>Post-secondary or Higher..................3
DD3b	If formal technical/medical training, please specify type.<br><br>**(Circle answer)**	MSc in M&E.......................................1<br>MSc in Health informatics ....2<br>MPH or other related master's degree.................3

Supplement to: Worku AG, Midekssa WD, Tilahun HA, et al. The impact of health information system interventions on maternal and child health service utilizations in Ethiopia: quasi-experimental study. *Glob Health Sci Pract*. 2024;12(6):2400145. https://doi.org/10.9745/GHSP-D-24-00145

		Physician .... 4<br>Health officer .... 5<br>Nurse/Midwife .... 6<br>HIT/IT .... 7<br>HEW .... 8<br>Other (specify)________ 96
DD4a	Years of employment	Year | Month
DD4b	Years of working with health data or RHIS/HMIS/CHIS	Year | Month
DD5a	Have you ever received formal RHIS/HMIS/CHIS training?<br><br>**(Circle answer)**	Yes .... 1<br>No 0
DD5b	If yes, did you receive training in RHIS/HMIS/CHIS RHIS-related activities in the last year?<br>**(Circle answer)**	Yes .... 1<br>No 0
DD5c	If Yes to DD5a, what type of formal RHIS training have you ever received?<br><br>**(Circle all that apply)**	Health statistics .... 1<br>HMIS/CHIS (data collection, transmission, storage, and/or data quality assurance) .... 2<br>Data analysis and use .... 3<br>General M&E .... 4<br>ICT .... 5<br>Other (specify)________ 96

Supplement to: Worku AG, Midekssa WD, Tilahun HA, et al. The impact of health information system interventions on maternal and child health service utilizations in Ethiopia: quasi-experimental study. *Glob Health Sci Pract*. 2024;12(6):2400145. https://doi.org/10.9745/GHSP-D-24-00145

SECTION 1.3: RHIS KNOWLEDGE		
U1	Indicate possible reasons for collecting or using aggregated data on a monthly basis for the following data types.<br>**(Circle your response either 1. Yes or 2. No.)**	**Responses**
U1A	The reasons for collecting or using aggregated diseases data:	
	a. To provide individual level care	1. Yes 0. No
	b. To know changes in magnitude/burden of selected diseases	1. Yes 0. No
	c. To triage patients who need urgent care and those who can wait for some time	1. Yes 0. No
	d. To identify disease outbreaks and take action to address epidemics	1. Yes 0. No
	e. To plan preventive and promotive activities	1. Yes 0. No
U1B	The reasons for collecting or using aggregated immunization data:	
	a. To know the coverage of effective intervention (immunization) for improving maternal or child health	1. Yes 0. No
	b. To improve diagnosis and treatment of under five children	1. Yes 0. No
	c. To take action for providing necessary resources (eg. staffing, equipment, vaccines, etc)	1. Yes 0. No
	d. To plan for immunization activities – developing targets for immunization	1. Yes 0. No
U1C	The reasons for collecting or using aggregated age/sex of patients/clients	
	a. To ensure equitable service coverage across people of all groups	1. Yes 0. No
	b. To know which group is affected by certain disease	1. Yes 0. No
	c. To get more funding	1. Yes 0. No
	d. To calculate workload of OPD and under-five clinic	1. Yes 0. No
	e. To know if the appropriate group is getting the relevant services	1. Yes 0. No
U1E	The reasons for collecting or using geographical data or residence of patients, i.e., where they come from	
	a. To plan preventive and promotive activities targeted to certain geographic areas	1. Yes 0. No
	b. To improve access and utilization of health services	1. Yes 0. No
	c. To determine the behavior of clients/population group	1. Yes 0. No
	d. For disease surveillance (to control epidemic/disease outbreaks)	1. Yes 0. No
U1F	Why are population data needed (e.g., number of people living in the catchment area)?	
	a. To use as denominator for calculating of indicators	1. Yes 0. No
	b. To plan the provision of various health services	1. Yes 0. No
	c. To calculate the workload of health facilities	1. Yes 0. No
	d. To know the knowledge and skill of health professionals	1. Yes 0. No

Supplement to: Worku AG, Midekssa WD, Tilahun HA, et al. The impact of health information system interventions on maternal and child health service utilizations in Ethiopia: quasi-experimental study. *Glob Health Sci Pract*. 2024;12(6):2400145. https://doi.org/10.9745/GHSP-D-24-00145

U2	Describe three dimensions of data quality.
1	
2	
3	
U3	Describe three ways (mechanisms) of ensuring data quality, relevant to your job classification/responsibilities.
1	
2	
3	

Part 2. OBAT: For Woreda Levels

Number	Question
SECTION 2.1: COMPETENCY TO PERFORM RHIS TASKS	
**THIS SURVEY IS FOR THE WOREDA, ZONE OR REGIONAL MANAGEMENT, CASE TEAM COORDINATOR AND RHIS MANAGER OR STAFF RESPONSIBLE FOR THE ANALYSIS AND INTERPRETATION OF AGGREGATE WOREDA/ZONAL/REGIONAL DATA.**<br><br>**WE WOULD LIKE YOU TO SOLVE THE FOLLOWING PROBLEMS ABOUT PROBLEM IDENTIFICATION AND SOLVING, COMPILING DATA, CALCULATING PERCENTAGES, AND PLOTTING AND INTERPRETING INFORMATION.**	
	CASE STUDY ON DATA QUALITY<br><br>Ato Mesfin, Woreda Health Office head, read a recent report prepared by the HMIS Officer after a supervision visit made to five out of eight health facilities in the woreda. The supervisor cross-checked the reported data with the recorded data from the source document and calculated verification factor. The supervision report showed that the average data accuracy for the indicator, antenatal care 1st visit (ANC1) was only 60% and Ato Mesfin felt very disturbed by it. "I need to take action," he said aloud. He set up a meeting with the entire Woreda health team to identify the reasons for the discrepancy and think about next steps to improve the data quality. After some discussion with his team about the potential reasons for the low percentage of data accuracy, the Woreda team started preparing an action plan for all health facilities in the Woreda.
PS1	Describe possible data quality problem in this scenario
PS2	Write potential reasons to the data quality problem.

a	
b	
c	
PS3	Major activities to improve the data quality.
a	
b	
c	
d	
e	
CD1	The estimated number of pregnant mothers in the woreda catchment area for the current period is 760. The health facilities in the woreda have registered 456 pregnant mothers for antenatal care first visit. Calculate the percentage of pregnant mothers in the woreda attending antenatal care in the same period.
CD2	The table below shows the monthly HIV counseling and testing (HCT) results for Dera Woreda. In this Woreda, government facilities provide HCT services. During a recent review of the data, it was discovered that youth (below age 24) account for a significant number of new HIV infections. In response to these data, health centers in Dera Woreda regularly review HCT data to inform decisions related to increasing the uptake of HCT services among youth.

Supplement to: Worku AG, Midekssa WD, Tilahun HA, et al. The impact of health information system interventions on maternal and child health service utilizations in Ethiopia: quasi-experimental study. *Glob Health Sci Pract*. 2024;12(6):2400145. https://doi.org/10.9745/GHSP-D-24-00145

HIV Counseling & Testing Monthly Summary, December 2009

		Facility #1		Facility #2		Facility #3		Facility #4	
		Age of client		Age of client		Age of client		Age of client	
**Indicator**		< 24 y.	≥ 24 y.	< 24 y.	≥ 24 y.	< 24 y.	≥ 24 y.	< 24 y.	≥ 24 y.
HCT 1	Number of clients counseled	341	401	61	226	501	623	108	151
HCT 2	Number of clients tested for HIV	339	399	53	220	494	600	108	151
HCT 5	Number of clients who tested positive	30	41	9	63	96	141	17	19
HCT 7	Number of clients referred to support groups	30	41	4	41	84	98	4	8

CD2a

Using the table above, calculate the proportion of clients who tested positive for HIV among those tested in Dera woreda disaggregated by age.

01. Less than 24 years old ____________________.

02. Equal to or greater than 24 years old ____________________.

CD2b

Develop a bar chart depicting the distribution across the ages of clients tested for HIV for the four facilities.

Supplement to: Worku AG, Midekssa WD, Tilahun HA, et al. The impact of health information system interventions on maternal and child health service utilizations in Ethiopia: quasi-experimental study. *Glob Health Sci Pract*. 2024;12(6):2400145. https://doi.org/10.9745/GHSP-D-24-00145

CD3a	Interpret the following graph: 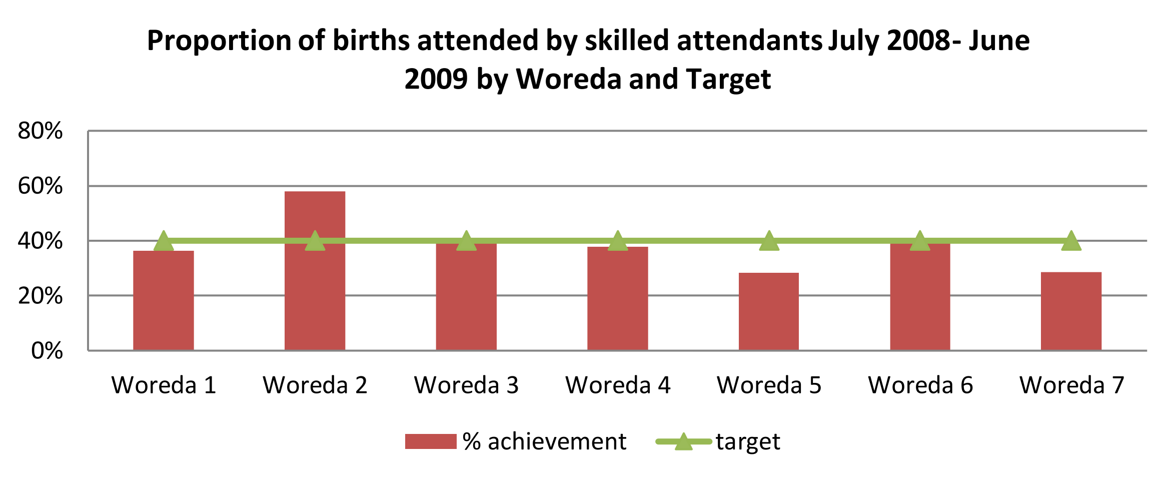
CD3b	Among the woredas shown in the above graph, which of the woredas have attained the target coverage rate (40%) by the end of 2009? In general, what possible guidance could you provide to woredas that didn't meet the target based on this data? (write a minimum of four solutions)

Supplement to: Worku AG, Midekssa WD, Tilahun HA, et al. The impact of health information system interventions on maternal and child health service utilizations in Ethiopia: quasi-experimental study. *Glob Health Sci Pract*. 2024;12(6):2400145. https://doi.org/10.9745/GHSP-D-24-00145

CD3c	Provide at least one use of the above chart findings at the:
CD3c	1. Facility level
CD3c	2. Community level
CD3c	3. Woreda level
CD4	A survey in the facility catchment area found 500 children under five years old that were malnourished. The total population of children less than five years old was 5,000. What is the malnutrition rate?
CD5	If the malnutrition rate in children less than two years old was 20% and the number of total children less than two years old was 10,000, then calculate number of children who are malnourished.

OBAT_113	Survey end time<br>(Use the 12-hour clock system)	☐☐ : ☐☐

Supplement to: Worku AG, Midekssa WD, Tilahun HA, et al. The impact of health information system interventions on maternal and child health service utilizations in Ethiopia: quasi-experimental study. *Glob Health Sci Pract*. 2024;12(6):2400145. https://doi.org/10.9745/GHSP-D-24-00145

Organizational and Behavioral Assessment Tool (OBAT)

For Health Facility

Purpose

5. Assess whether the organizational mechanisms are in place for producing the desired results in RHIS performance.
6. Explore the extent to which a culture of information exists in the organization.
7. Identify the commitment and support of upper management for enhancing an information system.
8. Quantify the health workers' motivation, knowledge, and skills to perform RHIS tasks.

Summary of Information Collected via the OBAT

3. Promotion of information culture
   - Emphasis on data quality
   - Use of RHIS information (for planning, day-to-day operations, and monitoring)
   - Problem solving, feedback
   - Sense of responsibility
   - Empowerment/accountability

4. Individuals behavior
   - Perception of self-competency to perform RHIS tasks
   - Knowledge of RHIS (including rationale for data collection and how to perform data quality checks)
   - Skills to perform RHIS tasks (such as identification and problem solving, visually presenting data, interpretation, and evidence-based decision-making)
   - Motivation

Data Collection Methods

Paper and pencil-based self-assessment to be administered by:

- Health facility (head, HMIS focal person, one case team/department head)

The OBAT has the following parts:

- Survey relevant for staff and management at all levels
- Cadre and level-specific competency surveys

Supplement to: Worku AG, Midekssa WD, Tilahun HA, et al. The impact of health information system interventions on maternal and child health service utilizations in Ethiopia: quasi-experimental study. *Glob Health Sci Pract*. 2024;12(6):2400145. https://doi.org/10.9745/GHSP-D-24-00145

Number	Question	Result
**SURVEY FACILITATOR**		
**OBAT_101**	Survey date	
**OBAT_102**	Interviewer name	
**OBAT_103**	Interviewer code<br>Please enter your 3-character identifier.	
**OBAT_104**	Type of facility	1 = Referral/Specialized hospital<br>2 = General hospital<br>3 = Primary hospital<br>4 = Health center
**FACILITY IDENTIFICATION**<br>**[Valid when facility type is 1, 2, 3, 4, 5 or 6]**		
**OBAT_105f**	Select Region<br>Please circle	1. Addis Ababa<br>2. Afar<br>3. Amhara<br>4. Beneshangul Gumuz<br>5. Dire Dawa<br>6. Gambella<br>7. Harari<br>8. Oromiya<br>9. SNNPR<br>10. Somali<br>11. Tigray<br>12. Sidama
**OBAT_107f**	Woreda/Sub-city<br>Please enter name of the woreda or sub-city	
**OBAT_109f**	Health facility name	
**OBAT_110f**	Urban/rural	Urban .... 1<br>Rural 2
**OBAT_111f**	Managing authority	Government/public .... 1<br>NGO/not-for-profit .... 2<br>Private-for-profit .... 3<br>Mission/faith-based .... 4<br>Other (specify) ________ 96

Supplement to: Worku AG, Midekssa WD, Tilahun HA, et al. The impact of health information system interventions on maternal and child health service utilizations in Ethiopia: quasi-experimental study. *Glob Health Sci Pract*. 2024;12(6):2400145. https://doi.org/10.9745/GHSP-D-24-00145

Part 1. OBAT: For Staff and Management at All Levels

Introduction

This survey is part of the ________________________ (indicate name of Program/Project leading the RHIS assessment), to improve Routine Health Information Systems in the health sector. The objective of this survey is to identify strengths and weaknesses in the system with a view to developing interventions for system strengthening. Please express your opinions honestly. Your responses will remain confidential and will not be shared with anyone, except in aggregate formats. We appreciate your assistance and co-operation in completing this study.

Thank you.

OBAT_112	Survey start time<br><br>(Use the 12-hour clock system)	☐☐ : ☐☐

Number	Question	Result
SECTION 1.1: RESPONDENT BACKGROUND		
DDI	Title of the person completing the questionnaire **(circle answer)**	Health facility head____1<br>Health facility HMIS focal person/data management staff/HIT____2<br>Health care provider/MCH case team lead____3<br>Other (specify)____96
DD2	Sex	Male____1<br>Female____2
DD3a	Highest level of education achieved.<br><br>**(Circle one)**	Primary/Elementary____1<br>Secondary/High School____2<br>Post-secondary or Higher____3
DD3b	If formal technical/medical training, please specify type.<br><br>**(Circle answer)**	MSc in M&E____1<br>MSc in Health informatics____2<br>MPH or other related master's degree____3<br>Physician____4<br>Health officer____5<br>Nurse/Midwife____6<br>HIT/IT____7<br>Other (specify)____96
DD4a	Years of employment	Year: ____ Month: ____

Supplement to: Worku AG, Midekssa WD, Tilahun HA, et al. The impact of health information system interventions on maternal and child health service utilizations in Ethiopia: quasi-experimental study. *Glob Health Sci Pract*. 2024;12(6):2400145. https://doi.org/10.9745/GHSP-D-24-00145

DD4b	Years of working with health data or RHIS/HMIS/CHIS	**Year** / **Month**
DD5a	Have you ever received formal RHIS/HMIS/CHIS training?<br><br>**(Circle answer)**	Yes .... 1<br>No 0
DD5b	If yes, did you receive training in RHIS/HMIS/CHIS RHIS-related activities in the last year?<br>**(Circle answer)**	Yes .... 1<br>No 0
DD5c	If Yes to DD5a, what type of formal RHIS training have you ever received?<br><br>**(Circle all that apply)**	Health statistics .... 1<br>HMIS/CHIS (data collection, transmission, storage, and/or data quality assurance) .... 2<br>Data analysis and use .... 3<br>General M&E .... 4<br>ICT .... 5<br>Other (specify)_______ 9 6

SECTION 1.3: RHIS KNOWLEDGE		
U1	Indicate possible reasons for collecting or using aggregated data on a monthly basis for the following data types.<br>**(Circle your response either 1. Yes or 2. No.)**	**Responses**
U1A	The reasons for collecting or using aggregated diseases data:	
	a. To provide individual level care	1. Yes 0. No
	b. To know changes in magnitude/burden of selected diseases	1. Yes 0. No
	c. To triage patients who need urgent care and those who can wait for some time	1. Yes 0. No
	d. To identify disease outbreaks and take action to address epidemics	1. Yes 0. No
	e. To plan preventive and promotive activities	1. Yes 0. No
U1B	The reasons for collecting or using aggregated immunization data:	
	a. To know the coverage of effective intervention (immunization) for improving maternal or child health	1. Yes 0. No
	b. To improve diagnosis and treatment of under five children	1. Yes 0. No
	c. To take action for providing necessary resources (e.g. staffing, equipment, vaccines, etc.)	1. Yes 0. No
	d. To plan for immunization activities – developing targets for immunization	1. Yes 0. No
U1C	The reasons for collecting or using aggregated age/sex of patients/clients	
	a. To ensure equitable service coverage across people of all groups	1. Yes 0. No
	b. To know which group is affected by certain disease	1. Yes 0. No
	c. To get more funding	1. Yes 0. No

	d. To calculate workload of OPD and under-five clinic	1. Yes 0. No
	e. To know if the appropriate group is getting the relevant services	1. Yes 0. No
U1D	The reasons for collecting or using geographical data or residence of patients, i.e., where they come from	
	a. To plan preventive and promotive activities targeted to certain geographic areas	1. Yes 0. No
	b. To improve access and utilization of health services	1. Yes 0. No
	c. To determine the behavior of clients/population group	1. Yes 0. No
	d. For disease surveillance (to control epidemic/disease outbreaks)	1. Yes 0. No
U1E	Why are population data needed (e.g., number of people living in the catchment area)?	
	a. To use as denominator for calculating of indicators	1. Yes 0. No
	b. To plan the provision of various health services	1. Yes 0. No
	c. To calculate the workload of health facilities	1. Yes 0. No
	d. To know the knowledge and skill of health professionals	1. Yes 0. No

Supplement to: Worku AG, Mideksa WD, Tilahun HA, et al. The impact of health information system interventions on maternal and child health service utilizations in Ethiopia: quasi-experimental study. *Glob Health Sci Pract*. 2024;12(6):2400145. https://doi.org/10.9745/GHSP-D-24-00145

U2	Describe three dimensions (aspects) of data quality.
1	
2	
3	
U3	Describe three ways (mechanisms) of ensuring data quality, relevant to your job classification/responsibilities.
1	
2	
3	

Part 3. OBAT: For Health Facility

Number	Question	Result
SECTION 3.1: COMPETENCY TO PERFORM RHIS TASKS		
**THIS SURVEY IS FOR A FACILITY MANAGEMENT, DEPARTMENT/CASE TEAM COORDINATOR OR STAFF RESPONSIBLE FOR THE ANALYSIS AND INTERPRETATION OF HEALTH FACILITY DATA.**<br><br>**WE WOULD LIKE YOU TO SOLVE THESE PROBLEMS ABOUT PROBLEM IDENTIFICATION AND SOLVING, COMPILING DATA, CALCULATING PERCENTAGES, AND PLOTTING AND INTERPRETING INFORMATION.**		
	CASE STUDY ON DATA QUALITY<br><br>The performance monitoring team of Gera Health Facility randomly selected 12 data elements from Yekatit monthly report and cross-checked the reported data with the recorded data from the relevant tally sheet and registers. They calculated the data accuracy using LQAS table. The result showed that the data accuracy score is only 70% and the team felt very disturbed by it. They set up a PMT meeting to identify the reasons for the discrepancy and think about next steps to improve the data quality.<br>After some discussion about the potential reasons for the low percentage of data accuracy, the team started preparing an action plan for their health facility.	
PS1	Describe possible data quality problem in this scenario.	
PS2	Write potential reasons to the data quality problem.	
a		

b	
c	
PS3	Write major activities to improve the data quality of Gera Health Facility
a	
b	
c	
d	
e	
CF1	The estimated number of pregnant mothers in the facility catchment area for the current period is 340. The antenatal clinic in your facility has registered 170 pregnant mothers. Calculate the percentage of pregnant mothers in the facility catchment area attending antenatal care.
CF2	The table below shows pregnant women attending antenatal care for the first time, as well as the number of these women who received Iron 90+ for prevention of anemia.

ANC Clinic - Health Center X												
**Indicator**	**Jul**	**Aug**	**Sep**	**Oct**	**Nov**	**Dec**	**Jan**	**Feb**	**Mar**	**Apr**	**May**	**Jun**
No. of ANC 1st visits	156	162	158	151	168	148	129	138	145	171	164	152
No. of women who received Iron 90+	101	110	107	106	121	105	97	109	117	144	143	138

Supplement to: Worku AG, Midekssa WD, Tilahun HA, et al. The impact of health information system interventions on maternal and child health service utilizations in Ethiopia: quasi-experimental study. *Glob Health Sci Pract*. 2024;12(6):2400145. https://doi.org/10.9745/GHSP-D-24-00145

CF2a	Develop a line graph depicting the trend over one year in Iron 90+ coverage among pregnant women attending ANC for the first time.
CF2b	What does the following graph tell you about the FP method mix for new users in a health center Y? 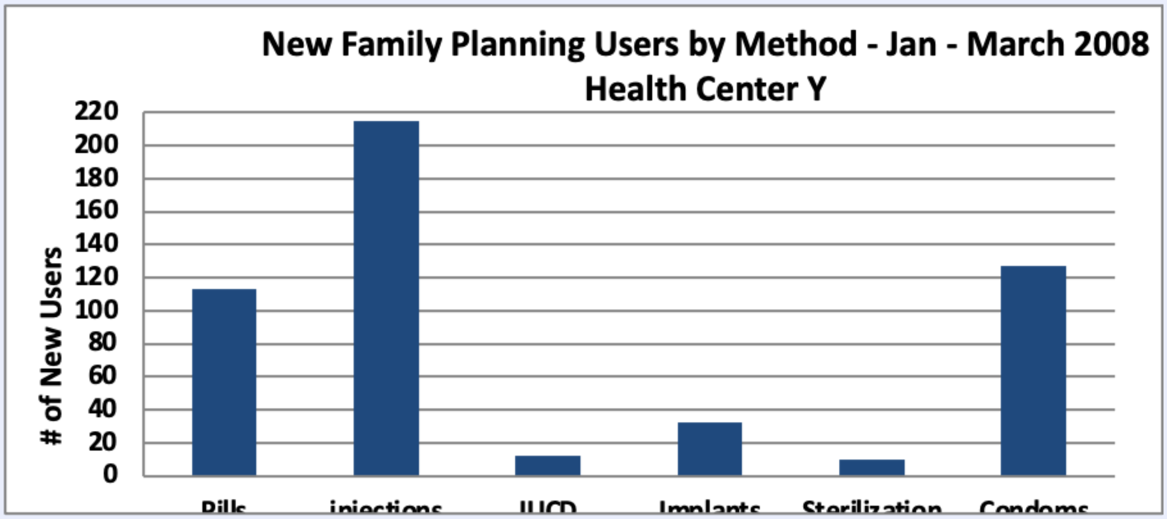

Supplement to: Worku AG, Midekssa WD, Tilahun HA, et al. The impact of health information system interventions on maternal and child health service utilizations in Ethiopia: quasi-experimental study. *Glob Health Sci Pract*. 2024;12(6):2400145. https://doi.org/10.9745/GHSP-D-24-00145

CF2c	The target for the health center Y for new clients on FP for the year 2008 is 1,200 clients. How many new clients would the facility need to have each month if new clients were evenly distributed by month?<br><br>Assuming that the health center has achieved the target in the first quarter as planned and it maintains this (the calculated monthly figure) number of new FP client enrollments for the next three quarters, will the target be reached by the end of the year?<br><br>0. No ☐ 1. Yes ☐
CF2d	Provide at least one use of above family planning graph findings at the:
CF2d1	Facility level
CF2d2	Community level
CF3	A survey in the facility catchment area found a total of 500 children (225 boys and 275 girls) under five years old that were malnourished. The total population of children less than five years old was 5,000, among them 55% were female.
CF3a	What is the malnutrition rate of boys?
CF3b	What is the malnutrition rate of girls?
CF3c	What information do you get by disaggregating the data by sex? How does this information help you in planning/improving your service delivery?
OBAT_113	Survey end time<br><br>(Use the 12-hour clock system) ☐☐ : ☐☐
