# Supplementary material for: The Impact of Health Information System Interventions on Maternal and Child Health Service Utilizations in Ethiopia: A Quasi-Experimental Study
Source: Glob Health Sci Pract. 2024 Dec 20;12(6):2400145. doi: 10.9745/GHSP-D-24-00145 (PMC11666088; doi:10.9745/GHSP-D-24-00145)
Supplement: GHSP-D-24-00145-Supplement3.pdf [file GHSP-D-24-00145-Supplement3.pdf]

Supplement to: Worku AG, Midekssa WD, Tilahun HA, et al. The impact of health information system interventions on maternal and child health service utilizations in Ethiopia: quasi-experimental study. *Glob Health Sci Pract*. 2024;12(6):2400145. https://doi.org/10.9745/GHSP-D-24-00145

Supplement 3. Operational definitions and measurements of key variables

Data quality indicators

1. Completeness of facility reporting

Proportion of expected facility monthly reports (by report type) that are actually received at the aggregate level.

Numerator: Total # of facility reports received at the aggregate level (by report type)

Denominator: Total # of expected facility reports at the aggregate level (by report type)

Type of reports: Service report, IPD report, OPD report and quarterly report

2. Timeliness of facility reporting

Percentage of submitted facility monthly reports (by report type) that are received on time (i.e. by the deadline of reporting). In Ethiopia, facilities are expected to submit reports by the 26th of each month and Woreda health offices are supposed to keep report receipt logbook.

Numerator: Total # of facilities that submitted reports (by type) on time to the aggregation site

Denominator: Total # of expected facility reports (by type) at the aggregation site

Type of reports: Type of reports: Service report, IPD report, OPD report and quarterly report

3. Completeness of source document

Percentage of facilities with completely filled primary source documents, such as registers, patient records, etc. for selected indicators (i.e., source documents contain the data relevant to the selected indicators). Source document completeness was assessed for the seven indicators: skilled birth attendance (SBA), pentavalent third dose (penta3), women received modern contraceptive methods (FP), clients who tested positive for HIV, confirmed malaria cases (malaria), under five pneumonia and TB cases (TB) for each of the three months in the health facilities.

Numerator: Total # of assessed facilities with a completely filled primary source document for selected indicators within the review period.

Denominator: Total # of assessed facilities expected to report on the selected indicators

Completely filled means 90% or more of the data elements relevant to the selected indicator are filled (i.e. only 10% of the fields are left blank for selected indicator)

Procedure: The assessor should take the last 15 entries recorded in the source document for each reporting period and check how many of the data elements relevant to the selected indicator are filled in. For example, for SBA we identified 7 relevant fields to be completed per client on the

register (MRN, Name, Age, Date of delivery, Mode of delivery, Maternal status, Newborn birth outcome). So we expect a total of 105 fields to be filled (15 x 7). A source document is considered as complete, if ≥ 90% (or 95) of the data elements are filled on the SBA register.

4. **Facility reporting accuracy (VF or reporting consistency)**

Percentage of facilities where data recorded in source documents are exactly matching reported data of selected indicator. This is the percentage of facilities that scored verification factor (VF) between 90% - 110% for selected indicators. Agreed tolerance/ acceptability error range by MOH is +/- 10%. It will be over-reporting if < 90% and under-reporting if > 110%.

The VF should be calculated for each selected indicator (SBA, Penta3, FP, HIV, malaria, pneumonia and TB) in 2 steps

Step 1. Calculate VF of each facility as follows:
Numerator: recounted data from the source documents for the selected indicator
Denominator: reported data from the monthly/quarterly report for the selected indicator
Step 2. Calculate the percent of facilities that have VF = 90% - 110% as follows:
Numerator: Number of facilities that have scored VF= 90% - 110% for the selected indicator
Denominator: Total # of facilities assessed that are providing service for the selected indicator

Data/ information use indicators

1. **Use of routine data for HMIS quality improvement**

It is the average score of health facilities using data to improve HMIS data quality. For each facility, the score is calculated based on evidence of Performance monitoring team (PMT) using data to improve HMIS data quality in the following areas (each positive response assigned 1 point); a) discussions on HMIS management, such as data quality, completeness, or timeliness of reporting, b) HMIS related issues identified and prioritized, c) root cause analysis conducted for the prioritized HMIS related issues, d) action plan developed to improving HMIS performance, and e) follow-up actions taken. The maximum scores for each facility is 5 (100%) if all checklist items present.

2. **Data use for quality control practices**

Measured by taking the average score of health facilities data quality control practice using the following indicators; a) HFs designated person or group in place to review data quality, b) HFs trained all designated staff on HMIS data review and quality control, c) HFs use data quality assessment tools, d) HFs conducted LQAS in the review period, e) HFs maintained records of the LQAs sheets conducted, and f) HFs maintain record of feedbacks given to staff based on data quality assessment. The maximum scores for each facility is 6 (100%) if all items present.

3. Use of routine data for performance review and evidence-based decision making

This is measured by taking the average score of health facilities using data for performance review using the following areas (each positive response assigned 1 point); a) discussed on key performance and quality of care indicators using HMIS data, b) identified and prioritized performance issues/problems, c) Conducted root cause analysis, d) made evidence-based decision, e) developed action plan for improving performance, and f) follow-up actions taken. The maximum scores for each facility is 6 (100%) if all items present.

4. Average score for level of data analysis practice

Measured by taking the average score of health facilities data analysis practice using the following checklist items (each practice assigned 1 point); a) aggregated/summary HMIS report, b) demographic data on the catchment population of the health facility for calculating coverage, c) indicators (e.g., Penta3 coverage) calculated for the health facility catchment within the review three months, d) comparisons between health facility and woreda/national targets, e) comparisons of data over time (monitoring trends) (e.g., for ANC, Penta3, etc.), f) comparisons of sex-disaggregated data (e.g., for Penta3, HIV testing, provider-initiated counseling and testing [PICT]), and g) comparisons of service coverage (e.g. ANC, TT immunization, SBA). The maximum scores for each facility is 7 (100%) if all practices present.

5. Use of routine data to produce analytic report

Percentage of health facilities with evidence of analytical report production using HMIS data.

6. Data visualization practice

Percentage of facilities that are using raw HMIS data to produce data visuals showing achievements toward targets. Data visuals refers to graphs, tables, maps, etc. showing achievements toward targets (indicators, geographic and/or temporal trends, and situation data).

7. Data dissemination outside the health sector

Average score for disseminating routine health information to stakeholders outside of the health sector calculated by observing evidence of; a) HMIS based report shared with the kebele/woreda constituency, b) Updated HMIS data shared electronically and/or printed media with the general public, and c) the health facility/woreda held performance review with the community representatives. Each positive response assigned 1 point and the maximum scores for each facility is 3 (100%) if all practices present.

8. Assessing PMT functionality

Series of question/checklist items were prepared to assess functionality of PMT. The 8 functionality areas include establishment of PMT in the HF, presence of regular PMT meetings

(≥3 in assessed 3 months), availability of at least one PMT meeting minutes, PMT meetings chaired by facility director (all 3 months), HFs discussed performance using HMIS data, HFs identified performance related problems, HFs conducted root cause analysis, and HFs developed action plan to improve performance.

MCH indicators

1. **Maternal Service coverage indicators**

Maternal service coverage indicators including ANC4 (at least 4 ANC visits), SBA (births attend by skilled provider), HFdel (delivered at health facility), PNC (having postnatal checkup) and EPNC are calculated by taking the proportion of service users from the total sample.

ANC4+: % of mothers receiving at least four antenatal care (ANC) service from a skilled health provider during their pregnancy

Numerator: Total # of targeted mothers who reported to attend at least four ANC service by skilled health provider

Denominator: Total # of mothers interviewed (have child aged 12 to 23 months)

Skilled birth attendance (SBA): Proportion of births attended by skill provider

Numerator: Total # of births attended by skilled provider

Denominator: Total # of eligible mothers (have child aged 12 to 23 months)

Delivery at health Facility(HFdel): Proportion of mothers delivered at health facility (health center or hospital)

Numerator: Total # of mothers who gave birth at HF

Denominator: Total # of eligible mothers (have child aged 12 to 23 months)

PNC: Proportion of mothers receiving postnatal care (PNC) service from a skilled health provider

Numerator: Total # of mothers who received PNC service from skilled provider

Denominator: Total # of mothers interviewed (have child aged 12 to 23 months)

Early PNC (EPNC): Proportion of mothers receiving postnatal care (PNC) service from a skilled health provider in the first 24 hours

Numerator: Total # of mothers who received PNC service from skilled provider in the first 24 hours

Denominator: Total # of mothers interviewed (have child aged 12 to 23 months)

2. **Quality of ANC services**

The average score of receiving essential ANC service components (quality of ANC services) is calculated based on the following 13 ANC services provided for the pregnant women during her visit; measure blood pressure measured, weight measured, blood sample taken, urine sample taken, TT (tetanus) vaccine provided, dietary advice provided, baby growth updated, due date known, counseled for delivery preparation, advised for health facility delivery, counseled for danger signs, tested for HIV, and communicated for HIV test results. The maximum scores for individual client (pregnant women having ANC visit) is 13 (100%) if all services are provided.

3. **Composite score for maternal care**

To develop the composite indicator, 3 categories of care, antenatal, intrapartum, and postnatal, with equal weights were included. The calculation used 9 indicators of essential interventions, 5 for antenatal, 2 for intrapartum, and 2 for postnatal care. The indicator score is calculated on a scale between 0–100; the ideal score is the maximum attainable score (utilization rate), which is 100; the target is the ideal performance of the indicator. For our analysis, we calculated the mean score of maternal care service utilization at the individual level.

$$Maternal\ care\ C = \frac{\frac{(ANC4 + EANC + TT + HIVtest + FeFol)}{5} + \frac{(SBA + HFdel)}{2} + \frac{(PNC + EPNC)}{2}}{3}$$

where ANC4 is at least 4 ANC visits, EANC is initiate early ANC visit (in the first trimester), TT is received TT vaccination, HIVtest is tested for HIV (as part of PMTCT), Fefol is received iron folic acid supplement, SBA is delivery by a skilled birth attendant, HFdel is delivered at health facility, PNC is having postnatal checkup, and EPNC is received early postnatal care (in the first 24 hours).

4. **Child immunization coverage indicators**

Similar approach applied for immunization coverages calculations for OPV3, Penta3 (DPT-HepB-Hib Pentavalent Vaccine), PCV3, MCV (measles), Rota2, BCG, VitA, and full vaccination (received all required doses vaccines).

Valid immunization coverage: Proportion of children who received immunization based on valid evidence (card or scar).

Numerator: Total # of children having immunization seen on card (for BCG: Card + BCG Scar)
<u>Denominator:</u> Total # of targeted children (12 -23 months of age)

Note: The following abbreviations represent vaccine types as follows
BCG: Bacillus Calmette–Guérin;
OPV: Oral polio vaccine

PCV: Pneumococcal conjugate vaccine
Pentavalent vaccine: The five vaccines including DPT (diphtheria, tetanus, pertussis), Hepatitis B, and Hemophilus influenzae type B vaccine (DTP-HepBHib)
MCV: measles containing vaccine
Rota: Rota virus vaccine
VitA: Vitamin A supplementation

5. Family planning indicators

Met need (Contraceptive Prevalence Rate): Proportion of mothers or their partners who are using a modern contraceptive method at the time of the survey (current contraceptive users).

Numerator: Total # of mothers/their partners currently using any contraceptive methods
Denominator: Total # of mothers included in the sample

Unmet need: Proportion of mothers who have unmet need for contraceptive use
Numerator: Total # of mothers with unmet need for contraceptive methods
Denominator: Total # of mothers included in the sample
