# Supplementary material for: The Impact of Health Information System Interventions on Maternal and Child Health Service Utilizations in Ethiopia: A Quasi-Experimental Study
Source: Glob Health Sci Pract. 2024 Dec 20;12(6):2400145. doi: 10.9745/GHSP-D-24-00145 (PMC11666088; doi:10.9745/GHSP-D-24-00145)
Supplement: GHSP-D-24-00145-Supplement1.pdf [file GHSP-D-24-00145-Supplement1.pdf]

Supplement to: Worku AG, Midekssa WD, Tilahun HA, et al. The impact of health information system interventions on maternal and child health service utilizations in Ethiopia: quasi-experimental study. *Glob Health Sci Pract*. 2024;12(6):2400145. https://doi.org/10.9745/GHSP-D-24-00145

Supplement 1. IR ASSESSMENT CHECKLISTS

Health Post IR Assessment Checklist

**Section A: Structure and Implementation of HIS (30%)**		
1	The health post has put in place the inputs needed to strengthen HIS	
	1.1	**The HP has the necessary input for Implementing CHIS**
		(1) Standard tickler file & Proper use,
		(2) Shelves
		(3) Family folders are properly filed
		(4) Family folders are easily accessible for service provision
	1.2	**At least two manuals which facilitate the implementation of HIS are in place**
		Updated CHIS user guide is available in the health post. A. YES B. NO
		Recent health post NCOD version is available. A. YES B. NO
	1.3	Availability of CHIS materials such as family folder, cards and tally sheet in past three months
2	The health facility has implemented eCHIS	
	2.1	The health post has started house hold registration for the implementation of eCHIS
	2.2	The health post has completed house hold registration and started service provision using eCHIS
**Section B: Data quality (30%)**		
1	Data Completeness and consistency on individual medical records and registers	
	1.1	Data completeness on the randomly selected family folders(rural HP) or register (Pastoralist HP)
2	Reporting accuracy/ Consistency between reports and records	
	2.1	In the last three months, the health post has conducted LQAS
	2.2	First LQAS score of the health center of the latest month of the reporting period for the monthly service delivery report
3	In the last three months, the health post has produced complete report	
	3.1	Does the health post keep a log book/uses electronic system that helps to track their reporting to HCs
	3.2	Report submission to health center<br>**Calculate as total number of reports the HP sent to health center/Total number of expected reports Total number of expected reports= types of report* period**
	3.3	Content completeness of service delivery report.<br>**Select 12 data elements randomly from the last month Service Delivery report and check the data complete- ness). Completeness= Data elements with complete data/12**
4	Reports in the last three months are submitted in a timely manner	
	4.1	The health post keeps logbook/uses electronic system that helps to track report submission timeliness
	4.2	Reports are submitted to the next level according to the national reporting schedule<br>*Calculate as total number of reports sent timely to the Health center/total number of expected reports Total number of expected reports= Types of report* period*
**Section C: Data use (40%)**		
1	The health post has developed annual action plan. A. YES B. NO	
2	HEW(s) participates in the kebele cabinet meeting:<br>A. Participated three times in the last quarter – 3 points<br>B. Participated two times in the last quarter – 2 points<br>C. Participated one time in the last quarter – 1 points<br>D. Never participated in the last quarter – 0 point	
3	Health Program performance was discussed in the kebele cabinet meeting<br>A. Health program performance was discussed three times in the last quarter – 5 points<br>B. Health program performance was discussed twice times in the last quarter – 4 points<br>C. Health program performance was discussed Once times in the last quarter– 2 points<br>D. Health program performance was never discussed in the last quarter – 0 point	

4	Review of key performance indicators	
	4.1	Plan versus achievement based on the key indicators<br>A. There is documented information that shows comparison was made between what is planned and what is achieved on the key indicators for all of the three months in the quarter- 9 points<br>B. There is documented information that shows comparison was made between what is planned and what is achieved on the key indicators for two months in the quarter- 6 points<br>C. There is documented information that shows comparison was made between what is planned and what is achieved on the key indicators only for one of the months in the quarter- 3 point<br>D. There is no documented information that shows comparison is made between what is planned and achieved based on the key indicators - 0 points
	4.2	Performance gaps are identified by comparing achievement against target (At least once in 3 months)
	4.3	Action plan is prepared for the identified priority problems/challenges<br>A. Action plan (with roles and responsibilities, resources and timeline) is prepared for all the identified priority problems/ challenges – 4 points<br>B. Action plan is prepared for some of the identified priority problems – 2 points<br>C. Action plan is not prepared at all – 0 points
	4.4	The action plan is being implemented<br>A. There is documented evidence for actions taken – 4 points<br>B. No action is taken– 0 points
5	The health post has a program performance monitoring chart (demographic profile, kebele resource mapping, school health profile and kebele HHs environmental sanitation profile)<br>A. All Key performance monitoring charts) are displayed – 10 points<br>B. At least half of monitoring charts are displayed-7 point<br>C. Few key performance monitoring charts are displayed – 5 points<br>D. No monitoring chart is displayed – 0 point	

Supplement to: Worku AG, Midekssa WD, Tilahun HA, et al. The impact of health information system interventions on maternal and child health service utilizations in Ethiopia: quasi-experimental study. *Glob Health Sci Pract*. 2024;12(6):2400145. https://doi.org/10.9745/GHSP-D-24-00145

Health Center IR Assessment Checklist

Section A: HIS structure and resources (30%)		
1	Medical record Unit	
	1.1	The health facility has the inputs necessary for medical record unit:
		1) Adequate space as per the standard? A. YES B. NO
		2) Adequate number of shelves? A. YES B. NO
		3) Adequate number of medical record unit staff? A. YES B. NO
		4) Availability of individual medical records (central register, Folder, service ID, patient card etc)?
	1.2	Proper procedures /processes in the medical record unit
		1) Centralized /unified medical record system/unit? A. YES B. NO
		2) Medical records are filed based on the label given on the shelves? A. YES B. NO
		3) The MRU is assisted by the use of an electronic system (Electronic Medical Record System)? A. YES B. NO
		4) Individual medical records are easily accessible for retrieving (Randomly take two samples and check if record easily (within three minutes for each) retrieved)? A. YES B. NO
		5) Completeness of central register? A. Complete B. Incomplete
2	HMIS Unit	
	2.1	The health facility has a well-organized HMIS unit.
		1. Dedicated desk/office? A. YES B. NO
		2. A functioning computer dedicated for DHIS 2 is in place? A. YES B. NO
		3. The HMIs unit has HIT or a personnel dedicated for the implementation of HMIS? A. YES B. NO
	2.2	At least five currently updated manuals which facilitate the implementation of HIS are in place
		1. HMIS procedure/data recording and reporting? A. YES B. NO
		2. HMIS Indicator reference guide? A. YES B. NO
		3. HMIS disease classification (NCoD)? A. YES B. NO
		4. Data quality manual? A. YES B. NO
		5. Data use manual? A. YES B. NO
	2.3	Availability of registers and tally sheet in past three months<br>*Calculate as (Registers and tally sheets that were available all the time during the reporting period/ number of applica- ble registers and tally sheets)*
3	The health center has running cost for strengthening and implementation of HIS? If the facility had budget to conduct any of the HIS activties such as printing, supervision, review meeting, then give YES.<br>A. Yes<br>B. No	
4	A supportive supervision with corresponding time frame is in place and is being implemented?	
	4.1	The health center conducted CHIS/integrated supportive supervision* to health posts in the past three month<br>*Calculate as (Total number of visits conducted/expected number of visits during the period)*<br>*(Expected number of visits equals= # of times HPs are visited* Number of months). A health center is expected to visit a health post monthly.*
	4.2	Supportive supervision is done using a checklist?<br>A. Yes<br>B. No
	4.3	Written supportive supervision report/feedback provided to the health posts<br>*Calculate as (Total number of feedback provided/ Total number of visits conducted during the period)*
	4.4	Action plan with responsible person and time table prepared and tracked implementation to facilitate follow-up?<br>A. Action plan developed and previous actions monitored<br>B. Action plan developed but previous actions not monitored<br>C. Action plan not developed

5		There is a documented evidence that shows HIS capacity building needs assessment is done (on data management, data quality, and data use) for the staff in the past 6 months.<br>A. HIS capacity needs assessment was conducted and took action/communicated to the next level in the last six months<br>B. HIS capacity needs assessment was conducted but didn't take action/was not communicated to the next level in the last six months.<br>C. HIS capacity needs assessment was not conducted in the last six months
6	Digitization	
	6.1	The health facility has a functional DHIS-2?<br>A. Yes and it is online<br>B. Yes but it is offline<br>C. No DHIS-2
	6.2	The health facility has an internet connectivity (functional HealthNet, 3G/4G etc)<br>A. Yes<br>B. No
**Section B: Data quality (30%)**		
1	Data Completeness and consistency on individual medical records and registers	
1.1	Data completeness of the registers.<br>*Calculate data completeness of the register*<br>*If register completeness check is not conducted, the facility gets 0*	
1.2	Data consistency between register and individual medical records for the indicator selected above during the last audit :<br>*Calculate the consistency between register to individual medical record*<br>*If consistency of data on register is not checked against individual medical record, the facility gets 0*	
1.3	Data completeness on the individual medical records for randomly selected MRNs from the central medical register:<br>*Calculate data completeness of the individual medical records*<br>*If register completeness check is not conducted, the facility gets 0*	
1.4	Data consistency between individual medical records and register for the selected MRNs from the central medical register:<br>*Calculate the consistency between register to individual medical record*<br>*If consistency of data on register is not checked against individual medical record, the facility gets 0*	
2	Reporting accuracy/ Consistency between reports and records	
2.1	The facility conducts data verification (reporting accuracy ratio) of reported and recounted data at department level before reporting to the HMIS unit<br>A. Yes<br>B. No	
2.2	In the last three months, the health facility has conducted LQAS: *Calculate as total number of LQAS conducted/expected number of LQAS Expected number of LQAS= Number of months * Types of reports*	
2.3	First LQAS score of the health center of the latest month of the reporting period for the monthly service delivery report:<br>*If the health facility has not conducted LQAS, it gets 0.*	
3	Completeness of reports (Representative and content)	
3.1	The health facility keeps a log book/uses electronic system that helps to track completeness and timeliness of reports<br>A. From both health posts and case teams<br>B. Only from one of the two<br>C. Never	
3.2	Completeness of reports (Representative) from health posts (Reports received from health posts) Calculate as total number of reports received from HPs/Total number of expected reports<br>*Total number of expected reports= Total number of Health posts * types of report* period*	
3.3	Completeness of reports (representative) from the health center/Primary health care unit (Reports sent from the health center)<br>*Calculate as total number of reports sent from the Health center/PHCU/Total number of expected reports Total number of expected reports= Types of report* period*	
3.4	Content completeness in the previous quarter<br>*(Select 12 data elements randomly from the last month Service Delivery report and check the data completeness). Complete - ness= Data elements with complete data/12*	

Supplement to: Worku AG, Midekssa WD, Tilahun HA, et al. The impact of health information system interventions on maternal and child health service utilizations in Ethiopia: quasi-experimental study. *Glob Health Sci Pract*. 2024;12(6):2400145. https://doi.org/10.9745/GHSP-D-24-00145

4	Timeliness of reports	
4.1	Health center has received reports from its health posts according to the national schedule *Calculate as total number of reports received timely from HPs/Total number of expected reports Total number of expected reports= Total number of Health posts * types of report* period*	
4.2	Reports are submitted to the next level according to the national reporting schedule<br>*Calculate as total number of reports sent timely from the Health center/PHCU/Total number of expected reports. Total number of expected reports= Types of report* period*	
**Section C. Data use (40%)**		
1	PHCU annual Woreda based plan:<br>A. The PHCU has annual plan which is cascaded to case team and Health post<br>B. The PHCU has plan but not cascaded to case team and health post<br>C. The PHCU doesn't have a plan at all	
2	Performance monitoring team (PMT) is in place and established according to national standard<br>A. PMT is in place and the members are put together based on the national standard<br>B. PMT is in place but the members are not put together based on the national standard<br>C. PMT is not established at all	
3	PMT is convening on monthly basis with 50%+ members<br>*Calculate as total number of PMTs held with 50%+ of its members/ Total number expected PMT meetings*<br>*Total number of expected PMT meetings equals the total number of months in the reporting period as it is expected to happen on a monthly basis*	
4	PMT is chaired by the head/delegate of the health facility as per the national standard<br>*Calculate as total number of PMTs chaired by head/delegate of the health facility/ Total number PMT meetings conducted*	
5	PMT is reviewing key performance indicators:	
	5.1	The health facility tracks performance/ indicators of all types (Coverage, quality and equity)
		5.1.1. Tracked key HMIS indicators (Plan versus Performance for key indicators)<br>*Calculate as total number of months where plan versus performance for key indicators is done/total number of months in the period*
		5.1.2. Evidence of analysis by any form of disaggregation (Age, sex, geography etc) A. YES B. NO
		5.1.3. Quality of health care (Content of care, standard of care etc) are measured and monitored by conducting clinical audit in the quarter?<br>A. YES<br>B. NO
	5.2	Performance gaps are identified<br>A. YES<br>C. NO
	5.3	Root cause analysis is done for low performing key prioritized indicators<br>A. Root cause is identified for all of the prioritized low performing key low performing indicators<br>B. Root cause is identified for only some of the prioritized low performing indicators<br>C. Root cause is not identified for all the low performing indicators
	5.4	Action plan/QI project with roles and responsibilities, resources and timeline is prepared for the identified priority problems/challenges<br>A. Action plan is prepared for all the identified priority problems/challenges<br>B. Action plan is prepared for some of the identified priority problems<br>C. Action plan has never been prepared at all
	5.5	The action plan/intervention/QI projects is being implemented<br>A. There is documented evidence that previous action plan is monitored during the current PMT<br>B. No action is taken
	5.6	PMT action plan/meeting minutes (Hard and/or soft) copy were shared to case teams<br>*Calculate as total number of times that case teams actually received the PMT meeting minute /[Number of case teams * number of months PMT meetings conducted]*
6	Updated DHIS-2 dashboard for health centers selected key indicators<br>A. The health center has an updated DHIS-2 dashboard for selected key indicators<br>B. The health center has a DHIS-2 dashboard for selected key indicators but it is not updated<br>C. The health center has never created a DHIS-2 dashboard for its key indicators	

7	Written feedback was given to lower level supervisory units (Health Posts) on the performance and data quality issues based on the routine report?<br>*Calculate as total number of written feedback given on performance and data quality issues / [total number of months in the period* total number of health posts]*
8	The health center practice Case team level performance review system<br>*Calculate as total number of times case teams conducted performance review/ Expected number of case team level performance and data quality review*<br>*Expected number of case team level performance review: [2 case teams (MCH and Disease)* 3 month =6)*
9	Service delivery units (EPI, OPD, Family planning, ANC/delivery/PNC, ART, TB) that have an updated program performance monitoring chart<br>*Calculate as total number of service delivery units that have a performance monitoring chart / total number of service delivery units in the facility*
10	The health facility has displayed information in the form of table, chart, etc. based on selected indicators in the health facility compound<br>A. Information (Performance related) is displayed in the health facility compound<br>B. No information (Performance related) was displayed in the health facility compound
11	Health facility held participatory performance review meeting with the community/stakeholders quarterly?<br>A. Yes<br>B. No
12	Health facility conducted review meeting (health Facility staff and health post staff)<br>A. The health facility conducted review meeting (any type: HIS/other program based) three times in the quarter<br>B. The health facility held review meeting (any type: HIS/other program based) twice in the quarter<br>C. The health facility held review meeting (any type: HIS/other program based) once in the quarter<br>D. The health facility did not hold performance review meeting in the last quarter
13	Presence of any gap addressed (Gap in performance, quality, equity)<br>A. Yes<br>B. No

Supplement to: Worku AG, Midekssa WD, Tilahun HA, et al. The impact of health information system interventions on maternal and child health service utilizations in Ethiopia: quasi-experimental study. *Glob Health Sci Pract*. 2024;12(6):2400145. https://doi.org/10.9745/GHSP-D-24-00145

Hospital IR Assessment Checklist

Section A: HIS structure and resources (30%)		
1	Medical record Unit	
	1.1	The hospital has the inputs necessary for medical record unit:<br>1) Adequate space as per the standard? A. YES B. NO<br>2) Adequate number of shelves? A. YES B. NO<br>3) Adequate number of medical record unit staff? A. YES B. NO<br>4) Availability of individual medical records (central register, Folder, service ID, patient card)
	1.2	Proper procedures /processes in the medical record unit<br>1) Centralized /unified medical record system/unit? A. YES B. NO<br>2) Medical records are filed based on the label given on the shelves? A. YES B. NO<br>3) The MRU is assisted by the use of an electronic system (Electronic Medical Record System)? A. YES B. NO<br>4) Individual medical records are easily accessible for retrieving (Randomly take two samples and check if record can easily (within three minutes for each) be retrieved)? A. YES B. NO<br>5) Completeness of central register? A. Complete B. Incomplete
2	HMIS Unit	
	2.1	The hospital has a well-organized HMIS unit.<br>1. Dedicated desk/office? A. YES B. NO<br>2. A functioning computer dedicated for DHIS 2 is in place? A. YES B. NO<br>3. The HMIS unit has HIT or a personnel dedicated for the implementation of HMIS? A. YES B. NO
	2.2	At least five currently updated manuals which facilitate the implementation of HIS are in place<br>1. HMIS procedure/data recording and reporting? A. YES B. NO<br>2. HMIS Indicator reference guide? A. YES B. NO<br>3. HMIS disease classification (NCoD)? A. YES B. NO<br>4. Data quality manual? A. YES B. NO<br>5. Data use manual? A. YES B. NO
	2.3	Availability of registers and tally sheet in past three months<br>*Calculate as (Registers and tally sheets that were available all the time during the reporting period/ number of applicable registers and tally sheets)*
3	The hospital has running cost for strengthening and implementation of HIS?<br>A. YES B. NO	
4	The hospital conducted HIS alone/integrated supportive supervision/mentorship* to health facilities in the past three months<br>Calculate as (Total number of visits conducted/expected number of visits during the period) (Expected number of visits equals= # of times centers are visited* Number of months)	
5	There is a documented evidence that shows HIS capacity building needs assessment is done (on data management, data quality, and data use) for the staff in the past 6 months.<br>A. HIS capacity needs assessment was conducted and took action/communicated to the next level in the last six months<br>B. HIS capacity needs assessment was conducted but didn't take action/was not communicated to the next level in the last six months.<br>C. HIS capacity needs assessment was not conducted in the last six months	
6	Digitization	
	6.1	The hospital has a functional DHIS 2?<br>A. Yes and it is online<br>B. Yes but it is offline<br>C. No DHIS-2
	6.2	The hospital has an internet connectivity (functional HealthNet, 3G/4G etc)<br>A. YES B. NO
	6.3	The hospital has implemented an Electronic health record (EHR) system<br>A. Full EHR<br>B. Partial EHR (Any of the service units except MRU)<br>C. None

Supplement to: Worku AG, Midekssa WD, Tilahun HA, et al. The impact of health information system interventions on maternal and child health service utilizations in Ethiopia: quasi-experimental study. *Glob Health Sci Pract*. 2024;12(6):2400145. https://doi.org/10.9745/GHSP-D-24-00145

	6.4	The hospital has implemented HRIS<br>A. YES B. NO
**Section B: Data quality (30%)**		
1	Data Completeness and consistency on individual medical records and registers	
1.1	Data completeness on the registers for one randomly selected data element from data elements listed on the most recent LQAS:<br>*Calculate data completeness of the register*<br>*If register completeness check is not conducted, the facility gets 0*	
1.2	Data consistency between register and individual medical records for the indicator selected above during the last audit :<br>*Calculate the consistency between register to individual medical record*<br>*If consistency of data on register is not checked against individual medical record, the facility gets 0*	
1.3	Data completeness on the individual medical records for randomly selected MRNs from the central medical register:<br>*Calculate data completeness of the individual medical records*<br>*If register completeness check is not conducted, the facility gets 0*	
1.4	Data consistency between individual medical records and register for the selected MRNs from the central medical register:<br>*Calculate the consistency between register to individual medical record*<br>*If consistency of data on register is not checked against individual medical record, the facility gets 0*	
2	Reporting accuracy/ Consistency between reports and records	
2.1	The facility conducts data verification (reporting accuracy ratio) of reported and recounted data at department level before reporting to the HMIS unit<br>A. YES B. NO	
2.2	In the last three months, the health facility has conducted LQAS: *Calculate as total number of LQAS conducted/expected number of LQAS Expected number of LQAS= Number of months * Types of reports*	
2.3	First LQAS score of the hospital for the last month of the reporting period for the monthly service delivery report:<br>*During verification: Take two data element from the sample data elements in the LQAS and check for correctness*	
3	Completeness of reports (Representative and content)	
3.1	The HMIS unit keeps a log book/uses electronic system that helps to track completeness and timeliness of reports from departments/case teams<br>A. YES B. NO	
3.2	Service Report completeness of the hospital (Reports sent- Representative completeness) *Calculate as total number of reports sent from the Hospital /Total number of expected reports Total number of expected reports= Types of report* period*	
3.3	Disease Report completeness of the hospital (Reports sent- Representative completeness) *Calculate as total number of reports sent from the Hospital /Total number of expected reports Total number of expected reports= Types of report* period*	
3.4	Content completeness in the previous three months<br>*(Select 12 data elements randomly from the last month Service Delivery report and check the data completeness). Complete - ness= Data elements with complete data/12*	
4	Timeliness of reports	
4.1	Service Reports of the hospital are sent according to the national reporting schedule (Service reporting timeliness)<br>*Calculate as total number of service reports sent timely /Total number of expected reports*	
4.2	Disease Reports of the hospital are sent according to the national reporting schedule (Disease reporting timeliness)<br>*Calculate as total number of disease reports sent timely /Total number of expected reports*	

Supplement to: Worku AG, Midekssa WD, Tilahun HA, et al. The impact of health information system interventions on maternal and child health service utilizations in Ethiopia: quasi-experimental study. *Glob Health Sci Pract*. 2024;12(6):2400145. https://doi.org/10.9745/GHSP-D-24-00145

**Section C. Data use (40%)**		
1		Availability of an annual hospital plan:<br>A. The hospital and each directorate has an annual plan<br>B. The hospital has an annual plan but targets not cascaded to directorates<br>C. The hospital doesn't have a plan at all
2		Performance monitoring team (PMT) is in place and established according to national standard<br>A. PMT is in place and the members are put together based on the national standard<br>B. PMT is in place but the members are not put together based on the national standard<br>C. PMT is not established at all
3		PMT is convening on monthly basis with 50%+ members<br>*Calculate as total number of PMTs held with 50%+ of its members/ Total number expected PMT meetings*<br>*Total number of expected PMT meetings equals the total number of months in the reporting period as it is expected to happen on a monthly basis*
4		PMT is chaired by the head/delegate of the health facility as per the national standard<br>*Calculate as total number of PMTs chaired by head/delegate of the health facility/ Total number PMT meetings conducted*
5		PMT/Quality improvement team is reviewing key performance indicators:
	5.1	The health facility tracks performance/ indicators of all types (Coverage, quality and equity)
		5.1.1. Tracked key HMIS indicators (Plan versus Performance for key indicators) A. YES B. NO
		5.1.2. Evidence of analysis by any form of disaggregation (Age, sex, geography etc) A. YES B. NO
		5.1.3. Quality of health care measured and monitored (Content of care, standard of care etc)- Clinical audit<br>*Calculate as total number of clinical audit conducted /Total number of expected clinical audits Total number of expected reports= Departments/case teams eligible for audit* period*
		5.1.4. The hospital conducted regular Perinatal/maternal death review<br>*Calculate as total number of times perinatal/maternal death review is conducted /Total number of expected reviews*
	5.2	Performance gaps are identified<br>A. YES B. NO
	5.3	Root cause analysis is done for low performing key priority indicators<br>A. Root cause is identified for all of the prioritized low performing key low performing indicators<br>B. Root cause is identified for only some of the prioritized low performing indicators<br>C. Root cause is not identified for all the low performing indicators
	5.4	Action plan with roles and responsibilities, resources and timeline is prepared for the identified priority problems/ challenges<br>A. Action plan is prepared for all the identified priority problems/challenges<br>B. Action plan is prepared for some of the identified priority problems<br>C. Action plan has never been prepared at all
	5.5	The action plan/intervention/QI projects is being implemented<br>A. There is documented evidence that previous action plan is monitored during the current PMT (Complete, partially complete and not implemented)<br>B. No action is taken
	5.6	PMT action plan/meeting minutes copy were shared to case teams<br>*Calculate as total number of times that case teams actually received the PMT meeting minute /[Number of case teams * number of months PMT conducted]*
6		Updated DHIS-2 dashboard for hospital's selected key indicators<br>A. The hospital has an updated DHIS-2 dashboard for selected key indicators<br>B. The hospital has a DHIS-2 dashboard for selected key indicators but it is not updated<br>C. The hospital has never created a DHIS-2 dashboard for its key indicators
7		Written feedback was given to service departments/directorates on the performance and data quality issues?<br>*Calculate as total number of written feedback given on performance and data quality issues / [total number of months in the period* total number of departments/directorates]*
8		The hospital practices Case team level/Directorate level performance and/or data quality review system<br>*Calculate as total number of times directorates conducted performance and data quality review / Expected number of directorate level performance and data quality review*<br>*Expected number of directorate level performance and data quality review: [Number of directorates* months]*
9		Directorates have a program performance monitoring chart<br>*Calculate as total number of directorates that have a performance monitoring chart / total number of directorates in the hospital*

10	The hospital has displayed information in the form of table, chart, etc. based on selected indicators in the health facility compound<br>A. Information (Performance related) is displayed in the health facility compound<br>B. Information (Performance related) is not displayed in the health facility compound
11	The hospital has developed and disseminated analytic(brochure and/or newsletter and/or leaflet and/or report and/or presentation etc) in the quarter<br>A. YES B. NO
12	The hospital held performance review meeting with the community quarterly?<br>A. YES B. NO
13	The hospital conducted review meeting in the quarter<br>A. The health facility conducted review meeting (any type: HIS/other program based) three times in the quarter<br>B. The health facility held review meeting (any type: HIS/other program based) twice in the quarter<br>C. The health facility held review meeting (any type: HIS/other program based) once in the quarter<br>D. The health facility did not held performance review meeting in the last six months
14	Presence of any change in performance, quality, equity as a result of use of data for action<br>A. YES<br>B. NO

Supplement to: Worku AG, Midekssa WD, Tilahun HA, et al. The impact of health information system interventions on maternal and child health service utilizations in Ethiopia: quasi-experimental study. *Glob Health Sci Pract*. 2024;12(6):2400145. https://doi.org/10.9745/GHSP-D-24-00145

Woreda Health Office IR Checklist

**Section A: HIS Structure and Resource (30%)**		
**1**	**The woreda health office has put in place the inputs needed to strengthen HIS**	
	1.1	The woreda health office has a well-organized monitoring and evaluation/planning unit or sub-unit<br>A. The woreda health office have adequate staff for planning, HMIS or M & E Unit as per the structure? A. YES B. NO<br>B. Dedicated office/Desk for planning/HMIS unit? A. YES B. NO<br>C. Presence of dedicated computer for HMIS/M&E unit? A. YES B. NO
	1.2	At least six currently updated manuals which facilitate the implementation of HIS are in place within the Woreda Health Office (accessibility should be indicated, either hard or soft copy)<br>A. HMIS procedure/data recording and reporting? A. YES B. NO (0.5)<br>B. HMIS Indicator Reference Guide? A. YES B. NO (0.5)<br>C. All HMIS disease classification booklets (NCoD)? A. YES B. NO (0.5)<br>D. Data quality Manual? A. YES B. NO (0.5)<br>E. Data use Manual? A. YES B. NO (0.5)<br>F. CHIS manual? A. YES B. NO (0.5)
**2**	**Does the woreda health office has budget for any of the below activities**	
	A	Supportive Supervision? A. YES B. NO
	B	Review meeting? A. YES B. NO
**3**	**The Woreda Health Office has a system for timely supportive supervision with fixed schedule**	
	3.1	Previous action plan reviewed before conducting next supportive supervision<br>A. YES<br>B. NO
	3.2	The Woreda Health Office conducted supportive supervision in the last quarter.
	3.3	Was the Supportive supervision done using checklist that address HIS components?<br>A. Yes the check list addressed major components of HIS<br>B. Yes the check list addressed partially HIS C. Checklist was used but it does not address HIS at all D. Checklist was not used at all
	3.4	Written supportive supervision report/feedback provided to the health facilities (observing written document is re- quired)
	3.5	Action plan with responsible person and time table prepared to facilitate follow-up?<br>A. YES<br>B. NO

Supplement to: Worku AG, Midekssa WD, Tilahun HA, et al. The impact of health information system interventions on maternal and child health service utilizations in Ethiopia: quasi-experimental study. *Glob Health Sci Pract*. 2024;12(6):2400145. https://doi.org/10.9745/GHSP-D-24-00145

**4**	**Capacity building and mentoring to strengthen the HIS in the past three months**	
	4.1	Woredal health office capacity for mentorship for HIS<br>A. There is trained mentors in the woreda<br>B. There is no trained mentors in the woreda
	4.2	The Woreda Health Office conducted mentoring at least once in the past three months-<br>A. YES<br>B. NO
	4.3	Action plan with responsible person and time table prepared to facilitate follow-up of gaps identified?<br>A. YES<br>B. NO
	4.4	Does the woreda conducted HIS capacity need assessment in the past six months?<br>A. YES, Observed<br>B. NOT Conducted
	4.5	Does the WorHO addressed the HIS capacity gap based on the findings?<br>A. YES, all are addressed<br>B. YES, Partially addressed<br>C. NOT addressed
**5**	**Digitization of HIS**	
	5.1	DHIS2 functionality during the past three months?
		A. YES functional, online<br>B. YES functional, offline<br>C. DHIS2 not functional at all
		WorHO practice backup storage of data monthly from DHIS2? A. YES B. NO
		WorHO staff have access to DHIS2? A. YES B. NO
		WorHO ensured the entry of denominator, baseline and target in DHIS2? A. YES B. NO
	5.2	Access to eCHIS dashboard by WorHO office head and woreda HMIS focal person<br>A. YES<br>B. NO
	5.3	The WoHO has a functional internet connectivity during the past three months?<br>A. YES<br>B. NO
	5.4	The WorHO regularly update master facility list<br>A. YES<br>B. NO
**6**	**Governance and leadership**	
	6.1	Does WorHO establish stakeholder or partner forum?<br>A. YES<br>B. NO
	6.2	Does WorHO level established stakeholder or partner forum meet regularly and monitor overall HIS performance<br>A. YES<br>B. NO

Supplement to: Worku AG, Midekssa WD, Tilahun HA, et al. The impact of health information system interventions on maternal and child health service utilizations in Ethiopia: quasi-experimental study. *Glob Health Sci Pract*. 2024;12(6):2400145. https://doi.org/10.9745/GHSP-D-24-00145

**Section B: Data Quality (30%)**		
**1**	**The woreda health office conducted RDQA**	
	1.1	The woreda health office had conducted verification component of RDQA in the last three months?
	1.2	In the last three months, to what extent the woreda has met the data accuracy target?<br>Calculate as total number of indicators whose data verification is b/n 0.9 and 1.1 / Total number of indicators for which data verification was done
	1.3	Data quality gaps improvement action plan was prepared based on data verification findings?<br>A. YES<br>B. NO
**2**	**Report Completeness**	
	2.1	The woreda Health Office keeps a log book that helps to track reporting completeness?<br>A. YES<br>B. NO
	2.2	Completeness of woreda administrative reports
	2.3	Completeness of reports (representative) from the Primary health care unit (Reports sent from the health center & health post)
**3**	**Report timeliness**	
	3.1	Timeliness of woreda administrative reports
	3.2	Health facilities in the woreda has submitted report according to the national schedule
4	WorHO conducted report completeness, timeliness and consistency analysis on monthly bases<br>A. YES<br>B. NO	
5	WorHO provide written feedback on report completeness, timeliness and consistency analysis monthly to health facilities.	
**Section C: Data Use (40%)**		
1	The Woreda based health sector (WBHS) plan:<br>A. The woreda has annual WBHS plan which is cascaded to health facilities and WorHO case team<br>B. The woreda has annual WBHS plan and cascaded the plan to either health facilities or WorHO case team<br>C. The woreda has WBHS plan but not cascaded to health facilities and WorHO case team D The woreda doesn't have a WBHS plan at all	
2	Performance management team(PMT) is in place and established according to national standard<br>A. PMT is in place and the members are put together based on the national standard<br>B. PMT is in place but the members are not put together based on the national standard<br>C. PMT is not established at all	
3	PMT is convening on a monthly basis with (50%+) members	
4	PMT is chaired by the head of the Woreda Health Office or delegates as per the national standard	
**5**	**PMT is reviewing key performance indicators**	

Supplement to: Worku AG, Midekssa WD, Tilahun HA, et al. The impact of health information system interventions on maternal and child health service utilizations in Ethiopia: quasi-experimental study. *Glob Health Sci Pract*. 2024;12(6):2400145. https://doi.org/10.9745/GHSP-D-24-00145

	5.1	The WorHO is tracking key coverage, quality and equity indicators
		5.1.1 The WorHO tracked key HMIS indicators (Plan versus Performance for key indicators)<br>Calculate as total number of months where plan versus performance for key indicators is done/total number of months in the period
		5.1.2 Evidence of analysis by any form of disaggregation (Age, sex, urban/rural etc)?<br>A. YES<br>B. NO
	5.2	Performance gaps are identified by comparing achievement against target (At least once in the quarter)?<br>A. Yes<br>B. No
	5.3	Root cause analysis is done for low performing key indicators, at least once in the review period for priority indicator<br>A. Root cause is identified for all low performing key priority indicators<br>B. Root cause is identified for only some low performing indicators<br>C. Root cause is not identified for all the low performing indicators
	5.4	Action plan is prepared for the identified priority problems/challenges with roles and responsibilities, resources and timeline<br>A. Action plan is prepared for all the identified priority problems/challenges<br>B. Action plan is prepared for some of the identified priority problems<br>C. Action plan is not prepared at all
	5.5	The action plan is being implemented (previous action plan reviewed before convening the subsequent PMT meeting and what has been done and not done is reviewed)<br>A. There is documented evidence for actions taken<br>B. No action is taken
	5.6	PMT action plan/meeting minutes/feedback were circulated to case teams: (written minutes with formal letter, can be made using social media platform or in person by hardcopy)
6	Updated DHIS2 dashboard for the woreda's selected key indicators<br>A. The woreda has an updated DHIS-2 dashboard for selected key indicators<br>B. The woreda has a DHIS-2 dashboard for selected key indicators but it is not updated<br>C. The woreda has never created a DHIS-2 dashboard for its key indicators	
7	Written feedback was given to lower level supervisory unit on the performance of Key indicators in the past three month	
8	The Woreda Health Office has presented or disseminated analytic report<br>A. Every Quarter<br>B. bi-annually<br>C. Annually<br>D. None	
9	Woreda health office case team practiced team level performance and data quality review	
10	The Woreda Health Office has displayed information in the form of table, chart, etc. based on selected indicators in the office compound using local language<br>A. Updated Information is displayed in the WoHO compound in local language<br>B. Updated Information is displayed in the Woreda Health Office compound in English<br>C. No information was displayed in the Woreda Health Office compound	
11	The Woreda Health Office held performance review meeting with community in the last quarter?<br>A. YES<br>B. NO	
12	The Woreda Health Office held performance review meeting with health facilities in the last quarter?<br>A. YES<br>B. NO	
13	Presence of any change in performance, quality, equity as a result of use of data for action<br>A. Yes<br>B. No	
