# Supplementary material for: The Impact of Health Information System Interventions on Maternal and Child Health Service Utilizations in Ethiopia: A Quasi-Experimental Study
Source: Glob Health Sci Pract. 2024 Dec 20;12(6):2400145. doi: 10.9745/GHSP-D-24-00145 (PMC11666088; doi:10.9745/GHSP-D-24-00145)
Supplement: GHSP-D-24-00145-Supplement4.pdf [file GHSP-D-24-00145-Supplement4.pdf]

Supplement to: Worku AG, Midekssa WD, Tilahun HA, et al. The impact of health information system interventions on maternal and child health service utilizations in Ethiopia: quasi-experimental study. *Glob Health Sci Pract*. 2024;12(6):2400145. https://doi.org/10.9745/GHSP-D-24-00145

Supplement 4. Additional tables

Health facilities reporting completeness (representativeness) at the woreda (district) level

	Baseline (2020)				Endline (2022)				Changes (%)		
	**Control**		**Intervention**		**Control**		**Intervention**				
Indicator	**N**	**Freq(%)**	**N**	**Freq(%)**	**N**	**Freq(%)**	**N**	**Freq(%)**	**Control**	**Interv**	**DID**
Service report	*198*	*198 (100)*	*264*	*232 (88)*	159	156(98)	147	147 (100)	-2	2	+4
IPD report	*129*	*84 (65)*	*111*	*48 (43)*	93	93 (100)	93	79(85)	35	42	+7
OPD report	*198*	*198 (100)*	*264*	*228 (86)*	159	156(98)	147	147(100)	-2	14	+16
Quarter report	*66*	*66 (100)*	*88*	*74 (84)*	53	52(98)	49	49(100)	-2	16	+18
Average score		*91.3*		*75.3*		98.5		96,3	7	21	+14

Report timeliness by heath facilities

	Baseline (2020)				Endline (2022)				Changes (%)		
	**Control**		**Intervention**		**Control**		**Intervention**				
Indicator	**n**	**Freq(%)**	**n**	**Freq(%)**	**N**	**Freq(%)**	**n**	**Freq(%)**	**control**	**Interv**	**DID**
Service report	*90*	*81(90)*	*126*	*75(60)*	159	96(60)	147	134(91)	-30	31	+61
IPD report	*66*	*26(39)*	*21*	*12(57)*	93	60(65)	93	44(47)	26	-10	-36
OPD report	*90*	*81(90)*	*126*	*73(58)*	159	96(60)	147	134(91)	-30	33	+63
Quarter report	*30*	*29(97)*	*42*	*22(52)*	53	32(60)	49	41(84)	-37	32	+67
Average score		*79*		*56.8*		61.3		78.3	-17.7	21.5	+39.2

Supplement to: Worku AG, Mideksa WD, Tilahun HA, et al. The impact of health information system interventions on maternal and child health service utilizations in Ethiopia: quasi-experimental study. *Glob Health Sci Pract*. 2024;12(6):2400145. https://doi.org/10.9745/GHSP-D-24-00145

Completeness of data elements in source documents

	Baseline (2020)				Endline (2022)				Changes (%)		
Indicator	**Control HF**		**Intervention HF**		**Control HF**		**Intervention HF**				
	**n**	**Freq(%)**	**n**	**Freq(%)**	**n**	**Freq(%)**	**n**	**Freq(%)**	**control**	**Interv**	**DID**
SBA	*37*	*27(73)*	*48*	*32(67)*	*38*	*35(92)*	*42*	*33(79)*	*19*	*12*	*-5*
Penta	*32*	*24(75)*	*41*	*35(85)*	*36*	*25(69)*	*41*	*34(83)*	*-6*	*-2*	*+4*
FP	*35*	*21(60)*	*47*	*37(79)*	*38*	*31(82)*	*41*	*39(95)*	*22*	*16*	*-6*
HIV	*37*	*20(54)*	*49*	*30(61)*	*37*	*31(84)*	*40*	*30(75)*	*30*	*14*	*-16*
Malaria	*34*	*24(71)*	*46*	*28(61)*	*35*	*26(74)*	*40*	*25(63)*	*3*	*2*	*-1*
Pneumonia	*37*	*26(70)*	*48*	*35(73)*	*38*	*23(61)*	*42*	*37(88)*	*-9*	*15*	*+24*
TB (quarter)	*34*	*30(88)*	*49*	*40(82)*	*36*	*34(89)*	*41*	*35(85)*	*1*	*3*	*+2*

Supplement to: Worku AG, Midekssa WD, Tilahun HA, et al. The impact of health information system interventions on maternal and child health service utilizations in Ethiopia: quasi-experimental study. *Glob Health Sci Pract*. 2024;12(6):2400145. https://doi.org/10.9745/GHSP-D-24-00145

Background characteristics of households and mothers

	Baseline			Endline		
HH charters tics	Control	Intervention	Both	Control	Intervention	Both
Owen house	75	80	78	72	79	76
Have Agri land	56	65	61	57	64	61
Have bank account	57	67	63	68	81	75
Natural floor/dung	76	78	77	75	73	74
Cement or brick floor	24	22	23	25	27	26
Thatch/leaf roof	19	21	20	17	12	14
Corrugated iron or Finished roof	81	79	80	83	88	86
Have pipe water access	62	66	64	72	72	72
Toilet Available	78	74	76	75	82	79
Access to electricity	52	49	51	57	61	60
**Total HH (n)**	**1276**	**1740**	**3016**	**1334**	**1743**	**3077**
**Distance from HF in km (mean)**	**3.79**	**3.54**	**3.63**	**4.05**	**3.5**	**3.74**
Mothers age (%)						
15-19 year	7	5	6	4	4	4
20-24 year	22	23	23	23	21	22
25-29 year	31	30	31	36	34	35
30-34 year	19	22	21	21	22	21
35-39 year	18	16	17	13	17	16
40-44 year	3	3	3	3	2	2
45-54 year	0.5	1	1	1	0.2	0.4
Married or partnered	94	94	94	95	94	94
Mothers religion						
Orthodox	39	45	43	45	46	45

Supplement to: Worku AG, Midekssa WD, Tilahun HA, et al. The impact of health information system interventions on maternal and child health service utilizations in Ethiopia: quasi-experimental study. *Glob Health Sci Pract*. 2024;12(6):2400145. https://doi.org/10.9745/GHSP-D-24-00145

	Baseline			Endline		
HH charters tics	Control	Intervention	Both	Control	Intervention	Both
Muslim	41	42	42	34	35	35
Protestant	17	12	14	20	18	19
Others religion	3	1	1	2	1	1
Mothers educational status						
No education	35	31	33	37	28	32
Elementary (1-8)	44	47	46	42	46	44
Secondary & above	20	22	21	21	27	24
**Occupation of mother**						
Employed	5	5	5	6	8	7
Farmer	26	30	29	20	25	23
Merchant	7	6	6	8	10	9
Unemployed	52	52	52	66	57	61
Others	9	7	8			
**Total mothers (n)**	**1276**	**1740**	**3016**	**1334**	**1743**	**3077**
Partners educational status						
No education	27	24	25	27	19	22
Elementary (1-8)	42	43	43	41	46	44
Secondary	20	22	21	20	21	20
Above secondary	11	11	11	12	14	13
Occupation of partner						
Employed	18	17	18	29	23	26
Farmer	49	55	52	49	56	53
Merchant	6	5	5	18	16	17
Unemployed	2	2	2	3	5	4
Others	25	21	23			
**Total partners (n)**	**1195**	**1637**	**2832**	**1263**	**1641**	**2904**
